# Questions regarding medication-related issues raised by legislators in the Indian parliament, 2017–2024

**DOI:** 10.1186/s12889-025-24278-8

**Published:** 2025-09-24

**Authors:** Preethi J. Shenoy, Rajeshwari Shastry, Ashwin Kamath

**Affiliations:** Department of Pharmacology, Kasturba Medical College Mangalore, Manipal Academy of Higher Education, Manipal, India

**Keywords:** Legislation, Vaccination, Drug regulation, COVID-19, Anticancer agents, Health infrastructure

## Abstract

**Background:**

Legislators’ questions in parliament serve as a crucial oversight mechanism, providing insights into prevalent health problems. This study analyzes questions asked by Indian members of parliament (MPs) regarding medications, including generic medicines and drug pricing, directed to the Health and Family Welfare Ministry from January 2017 to February 2024.

**Methods:**

Health-related questions raised by Indian MPs were extracted from the digital repository of the parliament. Medication-related questions were identified by keyword search using R statistical software (v4.1.2; R Core Team 2021) and manually confirmed. Each question/set of questions asked were converted to one sentence summaries using Microsoft Copilot (2025), and categorized into themes. Drugs, diseases, gender, and age groups addressed in each question were identified.

**Results:**

A total of 1121 medication-related questions were asked, constituting 19.42% of all health-related questions. Themes included cancer treatment, drug regulation, general health issues, health infrastructure, infectious diseases, maternal and child health, non-communicable diseases, and vaccination. COVID-19-related questions were prominent, followed by antimicrobials and haematinics. Post-COVID years saw increased questions on cancer chemotherapy and health infrastructure. Health delivery and drug regulation questions were consistently raised.

**Conclusions:**

Our study revealed an active dialog between legislators and the health ministry to address the population’s medication needs. The findings demonstrate that legislators are responsive to the changing disease patterns and challenges in treatment delivery. While our study examined medication-related questions in general, future research can focus on questions about specific drug groups or health issues to better understand the legislative influence on healthcare.

**Supplementary information:**

The online version contains supplementary material available at 10.1186/s12889-025-24278-8.

## Introduction

In a democratic country, the role of legislators is pivotal to ensuring the robustness of health preparedness and treatment facilities [1]. The continuous evaluation and enhancement of health services falls within the purview of legislators who play a central role in shaping policies and allocating resources for the betterment of the nation’s health infrastructure. Legislators act as intermediaries between the government and the public, actively engaging with their constituencies to understand healthcare challenges. Through community engagement and advocacy, legislators champion policies that address specific health concerns, ensuring that the health system is attuned to the diverse needs of the population [2]. Their active participation is crucial for achieving Sustainable Development Goal 3 of good health and well-being. For instance, Scotland’s legislation to ban smoking in public places significantly reduced hospital admissions for acute coronary syndrome [3]. Similarly, Australia’s National HPV Vaccination Programme substantially decreased human papillomavirus-related disease burden. However, health legislation can sometimes have unintended negative impacts or result in inequitable benefit distribution [4, 5]. Efforts in the United States to balance restricting nonmedical opioid use with ensuring availability for medical needs have been blamed for the growing opioid epidemic [6].

Question Hour in the Indian parliament serves as a crucial oversight mechanism, allowing Members of Parliament (MPs) to question the government on its governance strategies and policies, including medical treatment and health services [2]. MPs can raise urgent issues and inquire about the effectiveness of ongoing health programs and demand assessments, thus contributing to evidence-based policymaking in the health domain. Through questioning, MPs represent the concerns of their constituencies, bringing grassroots issues to the forefront and influencing health policies that directly impact local communities. The iterative nature of Question Hour ensures that health-related discussions are ongoing, fostering a dynamic process for continuous improvement and adaptation in the healthcare sector [7].

The Coronavirus disease 2019 (COVID-19) pandemic drew public and media attention to government actions worldwide, including India. Government actions during this health emergency, such as accelerating approval and implementation processes, highlighted its role in protecting national health [8]. For instance, a study on the Pradhan Mantri Jan Arogya Yojana (PMJAY) in 2018 analyzed legislative questions to gauge parliamentary discussion [9]. Similarly, an analysis of questions on diabetes mellitus in the Indian parliament showed a trend toward specific, solution-oriented inquiries, despite the relatively low number of questions [10].

However, apart from discussions on specific health issues/policies/programs initiated by the government, limited literature provides a comprehensive view of legislators’ questions regarding medication-related issues [10]. The government plays an important role in new drug approvals, quality testing of medicines, development of standard treatment guidelines, and ensuring equitable and rational drug use. Analyzing medication-related questions can reveal any discordance between legislators’ priorities and population needs. This study aims to determine the medication-related issues raised by MPs from 2017 to 2024, covering the pre- and post-COVID years, and to identify common diseases, drugs, and related regulatory and policy issues discussed in the Indian parliament.

## Method

This retrospective, descriptive analysis of questions by Indian MPs in parliament from 2017 to 2024, starting from the 16th term of the lower house of the parliament, House of People (Lok Sabha), XI session (start date 31 January 2017) to the 17th term of the lower house of the parliament, XV session (end date 10 February 2024). We extracted the questions from the digital repository of the proceedings of the House of People on 14th May 2025 [2]. The study protocol was approved by the Institutional Ethics Committee.

The questions concerning the Health and Family Welfare ministry were downloaded as Microsoft Excel files. The questions of interest were those related to drugs, which included vaccines and biological agents, as well as inquiries regarding the availability of drugs, price, prescribing, quality, regulatory aspects, etc. The investigators collaboratively generated a list of keywords likely to appear in questions related to medications. Keywords were used to identify relevant questions from the downloaded files using R statistical software (v4.1.2; R Core Team 2021) (Additional file 1). Nutraceuticals/nutritional supplements were included in the keyword search to avoid missing drug-related questions. Keywords were searched in both question and answer fields to ensure comprehensive identification. Filtered questions were manually reviewed to confirm that they were medication-related. Three investigators screened different sets of files. To ensure uniformity, 100 questions were initially reviewed independently, and differences were resolved through discussion, ensuring consistent assessments. The drug-related questions thus shortlisted were categorized based on whether the question pertained to (a) drug regulation or not; (b) disease prevention or treatment or both; (c) specific gender or not; (d) pregnancy and/or lactation; (e) children or adults or elderly or a combination of these; (f) specific disease; and (g) specific drug/drug group. Also noted was whether the question was starred or unstarred. Age group categorization was based on the description in the question/answer, unless specifically mentioned. Questions addressing multiple age groups were categorized as ‘All’, and those addressing multiple diseases or drugs were categorized as ‘Others’. Regarding the type of question asked, starred questions, identified in the database, require oral answers from the concerned minister and allow supplementary questions. Unstarred questions receive written responses without supplementary questions. Most questions were unstarred [2]. To determine the prevalent causes of mortality and morbidity during the study period, we extracted mortality and disability-adjusted life years (DALYs) data from the World Health Organization global health estimates for India [11], with the latest data available for 2021 [12].

The data are presented descriptively as numbers and percentages, as appropriate. The questions asked by legislators often have multiple parts, addressing the treatment of multiple diseases or related issues. To summarize this information, each question(s) was converted into a single sentence summary using Microsoft Copilot [Microsoft Copilot. (2025). Generated using Microsoft Copilot. Retrieved from https://copilot.microsoft.com.] for uniformity. Summaries of parliamentary questions were generated using GPT-4, a transformer-based large language model (OpenAI, 2023), accessed via the Microsoft Copilot platform. The model was employed to condense the content of each question into a concise summary while preserving its specificity and thematic relevance. To guide the summarization process, we initially provided Copilot with individual questions along with investigator-written summaries. This iterative approach was repeated until the model produced summaries that were deemed satisfactory by the investigators. The summaries thus generated were categorized into appropriate themes. To determine the reliability of the summaries generated and theme assignment, 10% (130 questions) of randomly selected summaries were reviewed and rated independently by two investigators as accurate or inaccurate. The inter-rater reliability was measured using Cohen’s kappa. A perfect agreement was obtained for the summaries generated (κ = 1, *p* < 0.01); a substantial agreement was obtained for the theme categorization (κ = 0.76 [95% CI, 0.49–1], *p* < 0.01). R software was used to generate the random number list and measure Cohen’s kappa.

To analyze word usage patterns across themes and years, a word frequency analysis was conducted using R (Additional file 2). Text data were first tokenized into individual words after removing short tokens and standard as well as domain-specific stopwords. All words were converted to lowercase and lemmatized to their base forms using the textstem package. A custom synonym mapping was applied to normalize semantically similar terms (e.g., “medicines”, “drugs”, and “medicine” were all mapped to “drug”). For each normalized term, the most frequent original word was selected as its representative. The final word frequencies were exported to an Excel file for further analysis and creating heatmaps to identify prominent terms and thematic clusters. The graphs and figures were created using BioRender [BioRender.com].

## Results

**Fig. 1:**
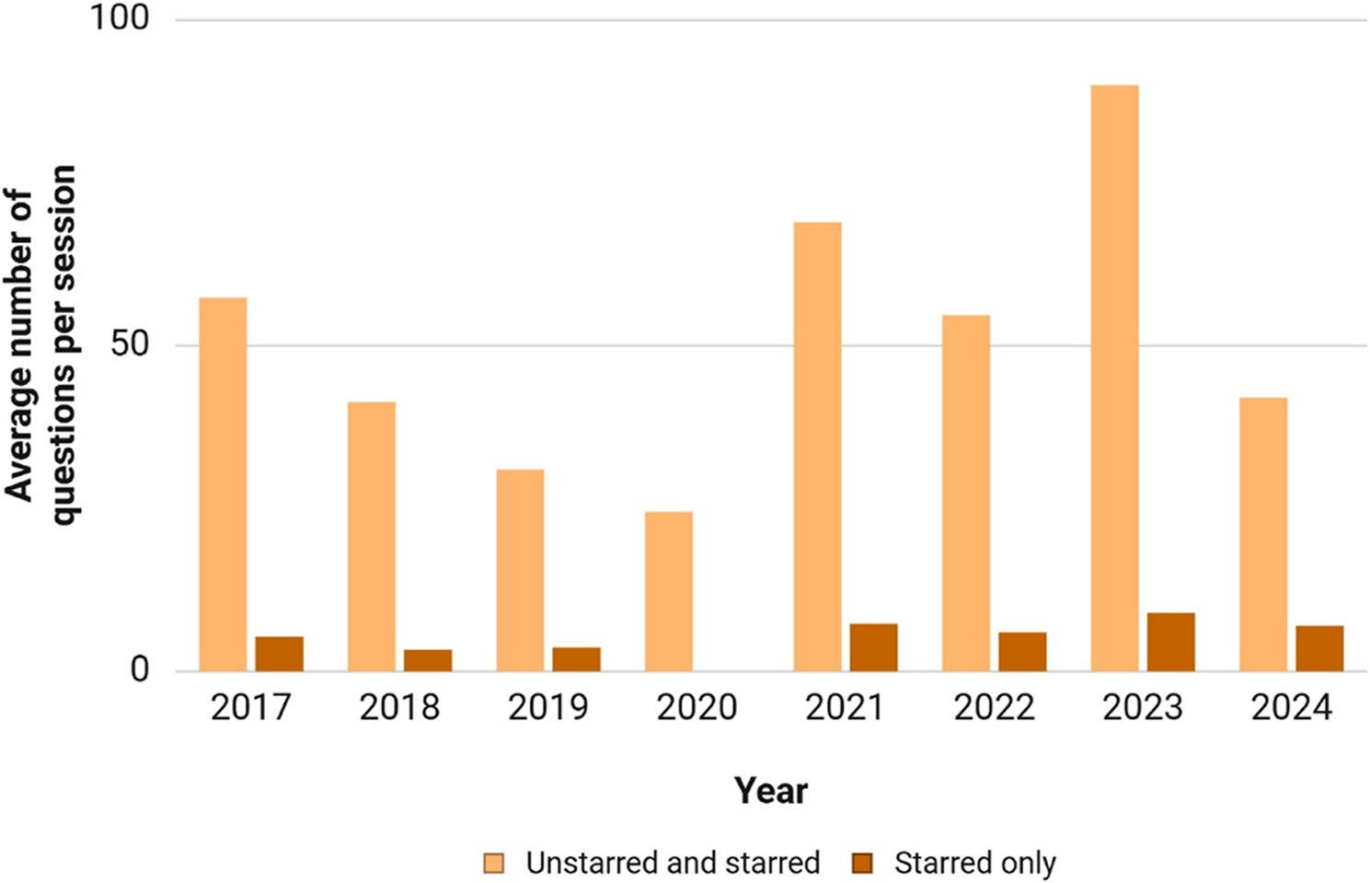
Average number of questions about medication-related issues raised by Indian legislators in the parliament per session, 2017–2024. The last parliamentary session in each year may have extended to January of the next year, but the questions have been considered based on the year the last session started. Only the first session in 2024 was completed at the time of data extraction

From January 2017 to February 2024, 5772 questions were directed to the Health Ministry by Indian MPs; of these, 19.42% (1121/5772) were medication-related questions. The year-wise distribution of the average number of questions per parliamentary session is shown in Fig. 1. Three parliamentary sessions are held each year; however, 2020 had only two sessions, and only one session of 2024 was completed at the time of data extraction. Of the 1121 questions, 9.9% (111/1121) were starred questions. 18.3% (205/1121) pertained to drug regulation; 49.9% (559/1121) questions were regarding the treatment of a specific disease. 7% (79/1121) of questions specifically addressed health issues in females, and only 0.1% (1/1121) addressed that in males; majority of the questions pertained to both genders. Of the 1121 questions, 32.1% (360/1121) were regarding drugs for disease prevention, 19.8% (222/1121) were about treatment, and the rest encompassed both. 5.7% (64/1121) of questions were regarding treatment during pregnancy/lactation. 10.4% (117/1121) questions pertained to children, 7.4% (83/1121) to adults, 0.1% (1/1121) to elderly, and the rest pertained to two or more of the age categories.

Regarding drug therapy for specific diseases, 20.2% (226/1121) were regarding COVID-19 infection, 4.1% (46/1121) regarding various types of cancers, 3.5% (39/1121) about anemia, 3.1% (35/1121) about tuberculosis, and 2.1% (23/1121) regarding diabetes mellitus. Furthermore, 50% (561/1121) were regarding treatment for two or more disease conditions or a general health-related/governance issue pertaining to drug use. From the pharmacotherapy perspective, 26.2% (294/1121) of questions pertained to vaccines, with 37.4% (110/1121) of these being regarding COVID vaccine. 10.4% (117/1121) of questions were on antimicrobials, with 26.5% (31/1121) of these regarding antitubercular drugs. Questions on hematinics constituted 3.1% (35/1121). 50.2% (563/1121) pertained to two or more drugs or other treatment modalities.

Each question(s) was summarized into a concise statement and categorized into nine themes. The themes identified were cancer treatment and research, drug regulation and use, general health issues, health infrastructure, infectious disease and their treatment, maternal and child health, mental health, non-communicable diseases, and vaccines and immunization. The number of questions pertaining to each theme is shown in Table 1.

**Table 1:** Number of questions about medication-related issues Raised by the Indian legislators in the parliament, 2017–2024, based on identified themes 225 questions were assigned more than one theme

Theme	Number of questions
Cancer treatment and research	71
Drug regulation and use	352
General health issues	231
Health infrastructure	155
Infectious diseases and their treatment	99
Maternal and child health	133
Mental health	22
Non-communicable diseases	32
Vaccines and immunization	251

### Starred questions

One-hundred and eleven starred questions were asked during the study period (Fig. 1). 6.3% (7/111) of questions specifically pertained to females, while the rest included both genders. 10.8% (12/111) questions were specifically about medications for diseases in children, 8.1% (9/111) in adults, and the rest included various age groups. 31.5% (35/111) were regarding disease prevention, 15.3% (17/111) regarding treatment, and the rest covered both aspects. 50.5% (56/111) of questions were regarding a specific disease, and 16.2% (18/111) were regarding drug regulation. 25.2% (28/111) of the questions were regarding vaccines, with more than half being about COVID-19 vaccines. This was followed by hematinics, 5.4% (6/111), and anticancer drugs, 4.5% (5/111).

**Fig. 2:**
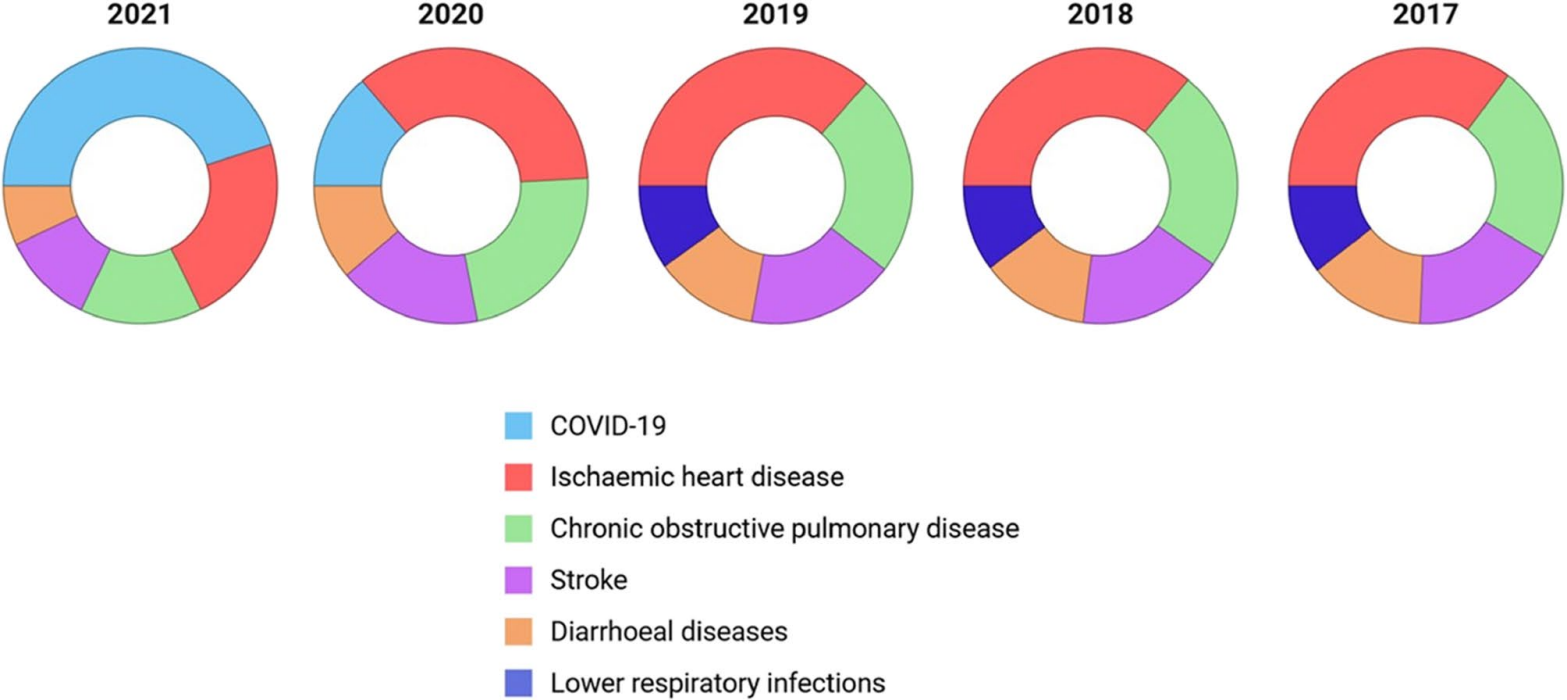
Top five causes of death in India from 2017 to 2021 based on World Health Organization global health estimate data [11]

**Fig. 3:**
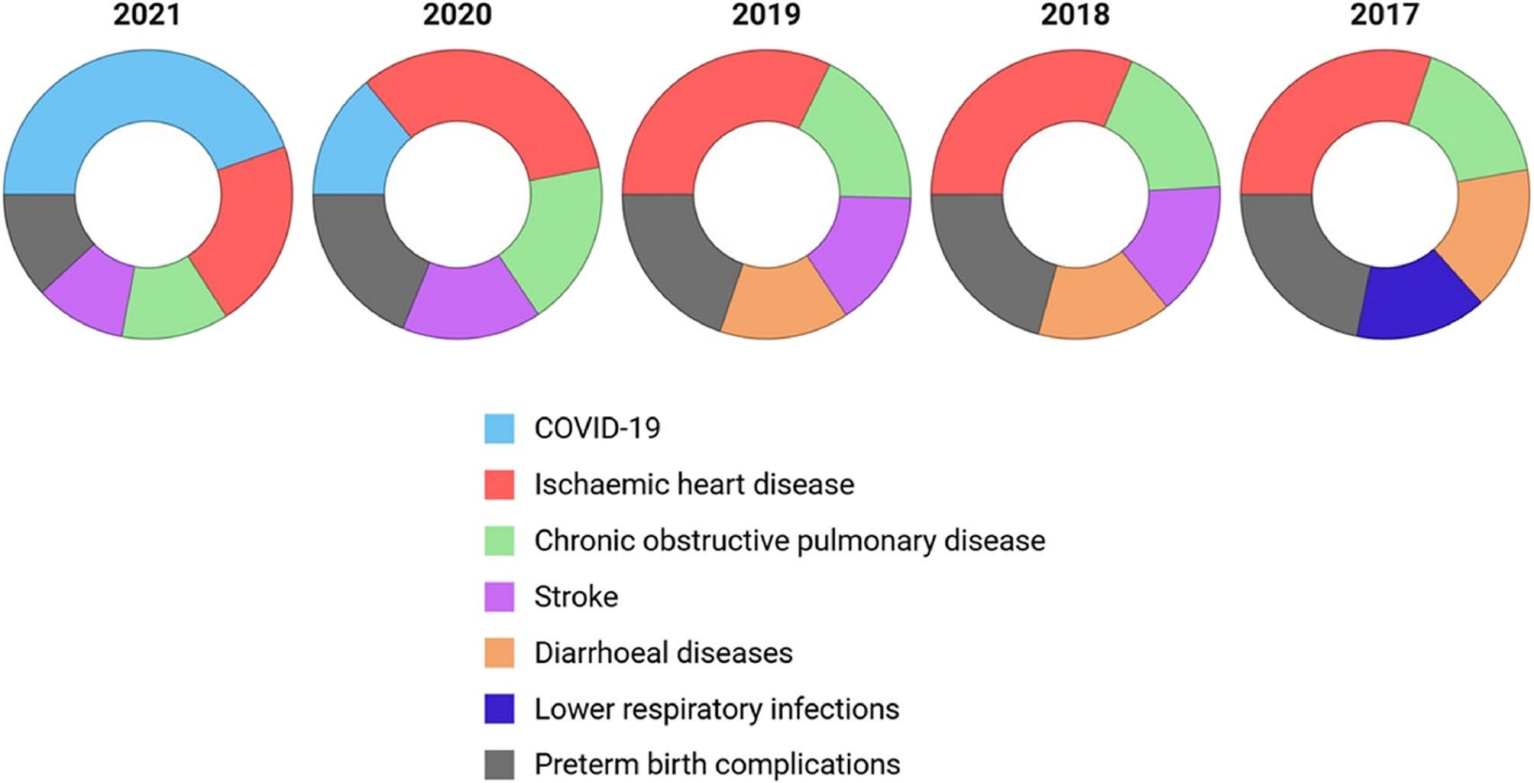
Top five causes of disability adjusted life years (DALYs) in India from 2017 to 2021 based on World Health Organization global health estimate data [11]

As per the World Health Organization global health estimates, ischemic heart disease, chronic obstructive pulmonary disease, stroke, and diarrheal diseases are among the top 5 leading causes of death in India from 2017 to 2021. Deaths due to lower respiratory tract infections were replaced with COVID-19 deaths (Fig. 2). In terms of DALYs, besides the aforementioned conditions, diarrheal diseases also contributed to morbidity burden in the latter part of the study period (Fig. 3).

**Fig. 4:**
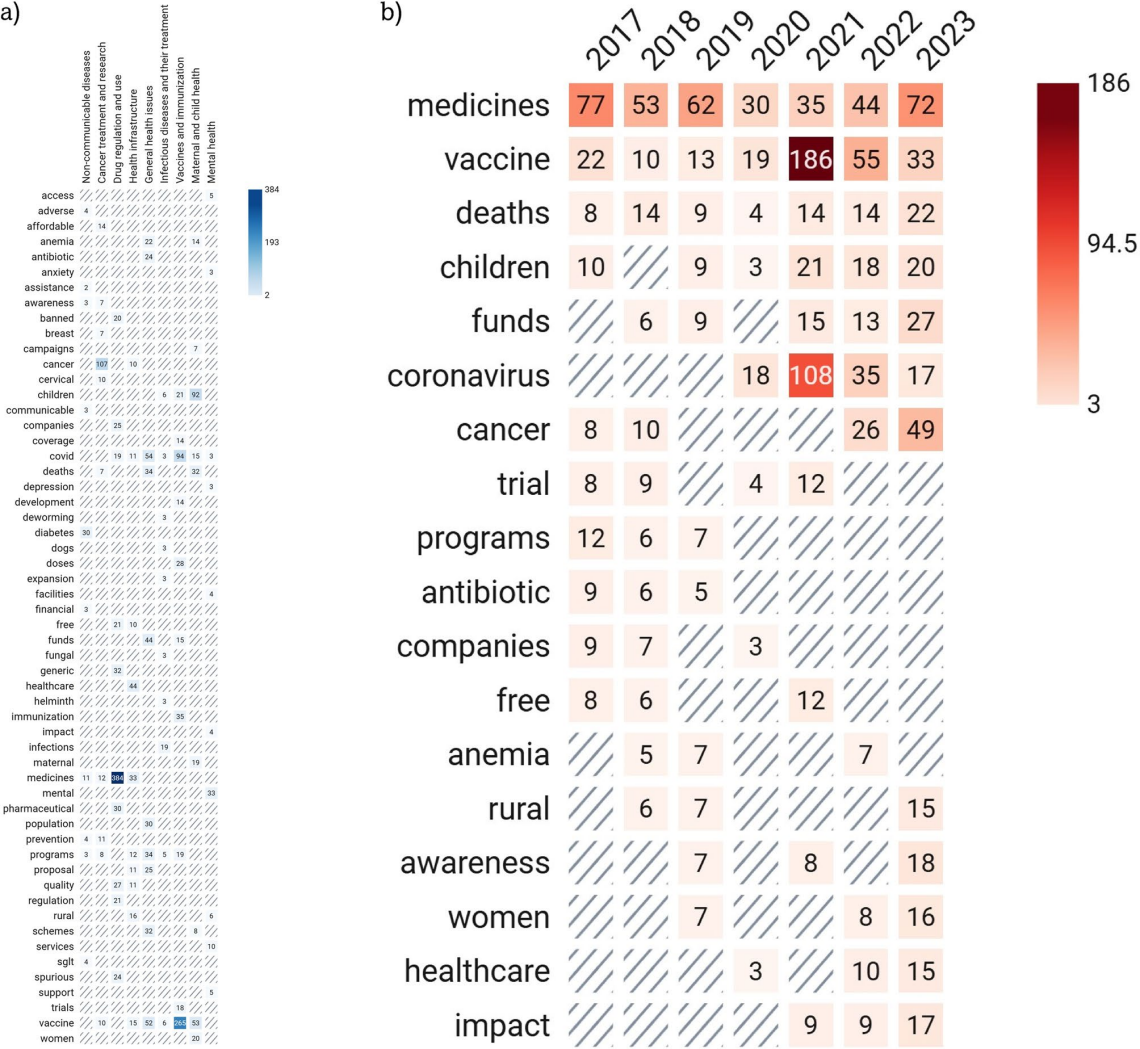
Heatmap of common keywords appearing in questions about medication-related issues raised by Indian legislators in the parliament, 2017–2024*, based on **a**) theme and **b**) year. The top 20 words in each theme or year were retrieved. Nonspecific words (e.g. rate, phase) were deleted. The rest have been presented in the heatmap of theme; for years, only those words that appeared in ≥ 3 years have been presented. The last parliamentary session in each year may have extended to January of the next year, but the questions have been considered based on the year the last session started. Only the first session in 2024 was completed at the time of data extraction

Figure 4 presents heatmaps of some of the keywords identified in the questions asked during the study period. The various questions asked during each year are presented in a summary form in Additional file 3.

## Discussion

Most legislative questions focused on drug regulation and use, followed by vaccines/immunization, general health issues, and health infrastructure. Despite ischemic heart disease, chronic obstructive pulmonary disease, and stroke being leading causes of morbidity and mortality, few questions have addressed their treatment directly. However, general health inquiries often included concerns about the pricing and availability of related medications. To the best of our knowledge, this is the first systematic analysis of such questions by Indian legislators. The questions reflected prevailing health concerns and aspects of drug use. Notably, there was a sharp decline in 2020 due to disruptions in parliamentary sessions caused by the COVID-19 pandemic (Fig. 1).

Many questions focused on drug regulation and disease prevention, addressing the entire population by age and gender. Health issues in children and women were consistently raised, highlighting their importance to legislators. Questions about specific diseases such as COVID-19, cancer, anemia, tuberculosis, and diabetes mellitus were prominent (Fig. 4), with COVID-19 inquiries peaking from 2020 to 2022 [13]. Post-COVID, cancer and mental health questions increased. The pandemic significantly impacted access to cancer chemotherapy, especially in rural areas, prompting government efforts to expand cancer care programs [14]. Questions about specific drugs aligned with prevalent diseases. Ischemic heart disease, chronic obstructive pulmonary disease, and stroke were the top contributors to mortality and DALYs (Figs. 2 and 3), indicating a potential discordance with legislators’ perceived health priorities. Non-communicable diseases were addressed in general inquiries about medicine availability in rural and publicly funded health setups (Additional file 3). Frequent questions on anemia reflect its impact on susceptibility to diarrheal and respiratory illnesses, birth complications, and long-term maternal and child health, particularly among lower socioeconomic groups. Government efforts to reduce noncommunicable diseases focus on common risk factors and early diagnosis screening, although grassroots implementation remains challenging [15]. Inquiries about vaccination were constant throughout the study period, with a significant increase during the COVID-19 pandemic. This aligns with extensive debates on vaccine development, procurement, availability, eligibility, efficacy, and safety [16, 17]. Vaccination for other preventable diseases was also consistently raised before and after the COVID period (Fig. 4) [18, 19].

Deliberations on many of the above-mentioned issues have occurred alongside measures to mitigate the problems identified or policy/regulatory changes. For example, the COVID pandemic prompted several regulatory measures to ensure that potentially therapeutic drugs were available for clinical use via the accelerated approval pathway [20]. Moreover, in order to be prepared for such pandemics in future, changes were brought about in the medical curriculum by incorporating a pandemic module that focuses on the process of drug approval, clinical trials, and adverse event reporting [21]. Cancer has been another major issue garnering legislators’ attention given the increasing burden. A major issue in cancer care in India is access to anticancer medications and availability of trained medical personnel to administer them [22]. The former is being handled by providing select anticancer treatment packages free of cost, a national health insurance scheme to reduce out-of-pocket expenditure, and capacity building of available healthcare set-ups for early diagnosis and treatment of cancer [14, 23].

Regarding the themes identified, questions on general health issues and drug regulation comprised over 50% of the inquiries (Table 1). General health questions included the availability of generic medicines and treatment in rural areas, whereas drug regulation questions covered quality testing, drug approval, clinical trials, medicine imports, and availability of scheduled drugs without prescription. This aligns with the health ministry’s role in ensuring safe and equitable distribution of medicines [24, 25]. The other key themes were vaccination and health infrastructure. Besides COVID vaccination, questions about vaccine-preventable diseases like hepatitis, mumps, measles, rubella, and rotavirus diarrhea were consistently raised. Health infrastructure questions are crucial because effective treatment delivery depends on adequate infrastructure. The Indian government has made efforts to provide free or economical treatment for various diseases [26, 27], and inquiries regarding their availability and quality help ensure that program goals are met.

Maternal and child health has been a significant focus for legislators. Despite government efforts to ensure adequate nutrition for children, adolescents, and women of reproductive age, and to curb communicable diseases, preventable childhood and maternal illnesses remain a significant burden due to the large population [28, 29]. Cancer is also gaining increasing attention, with legislators raising questions about the disease burden and the availability of anticancer drugs, particularly in rural and underprivileged areas [30, 31].

Legislative debates on health issues are influenced by evidence [32], public opinion [33], and political ideology [34]. Democratic governance positively impacts public health [35], as elected representatives respond to constituency demands, making health issues a political agenda [36]. In contrast, nondemocratic setups, like China, may lack public pressure, contributing to inadequacies in handling public health crises, such as during the COVID pandemic [37]. However, democratic governance alone does not ensure maximum health reforms; various internal and external drivers influence policymaking and implementation [38, 39]. The success of health systems does not always correlate with the type of democratic setup or economic conditions [40, 41]. The extent of evidence-based decision-making by legislators varies between countries. The World Health Organization Inter-Parliamentary Union assemblies aim to bridge the science-policy gap by ensuring that policymakers consider contemporary research evidence [1]. Institutionalization of mandatory annual health briefings for MPs, focusing on disease burden, drug regulation, and treatment access, using data from WHO, ICMR, and the National Health Mission, can aid legislators in evidence-based decision-making, ensuring proper resource allocation and policy formulation [42]. These briefings could be facilitated by the Ministry of Health and Family Welfare in collaboration with public health experts. Considering the views of all stakeholders, including doctors, public health experts, patient advocacy groups, and non-governmental organizations, can lead to a more comprehensive and acceptable health policy [43]. The formation of a Parliamentary Standing Committee on Medicines and Health Technologies could ensure sustained oversight on drug regulation, pricing, and availability. Legislators could also be mandated to undergo orientation sessions on rational drug use and essential medicines policy, especially when newly elected. Our study used word frequency analysis to describe patterns across themes and years. Probabilistic topic modeling is another approach that has been used in various areas of research, such as understanding social elements of decision-making based on study of meeting transcripts of a US FDA medical device panel [44] and content analysis of government documents to understand policy responses to ecological disturbances [45]. Word frequency analysis was selected over probabilistic topic modeling methods, such as Latent Dirichlet Allocation (LDA), due to its interpretability, transparency, and alignment with the study’s objectives. The primary goal was to identify and visualize dominant terms within parliamentary summaries to validate predefined thematic categories and support manual classification. Word frequency analysis offers a direct, reproducible approach that is well-suited for structured datasets and policy-oriented texts, where themes are often explicit. In contrast, LDA, while powerful for uncovering latent topics, introduces complexity in interpretation and requires subjective labeling of inferred topics [46].

Our study has limitations. The question files were manually reviewed by the authors to identify drug-related questions, with some assessed in duplicate to ensure uniformity. However, given the variety of questions, some assessments may not have been uniform, but any inconsistencies are unlikely to affect the overall findings. Oral parliamentary debates and state-level questions were not included, potentially missing some legislators’ views. Questions addressing multiple drugs/diseases/age groups were categorized as ‘Others’ or ‘All,’ reducing the granularity of the findings, but compensated by heatmaps and summaries (Additional file 3). Summaries were generated using AI and manually checked for accuracy. Session lengths varied, affecting the number of questions asked, with 2020 impacted by COVID restrictions and other sessions influenced by the political environment. The multi-party system and differing political ideologies may influence the nature of questions, which were not considered in this study.

## Conclusion

This study highlights active dialog between Indian legislators and the health ministry to address the population’s medication needs. Most questions focused on general health delivery, drug regulation, and health infrastructure, indicating efforts to address medication delivery to the needy. Questions on specific drug issues, such as COVID treatment, vaccination, cancer chemotherapy, maternal and child health, and generic medicines, demonstrate legislators’ responsiveness, in terms of the content and purpose of the concerns raised, to changing disease patterns and treatment challenges. Future research can focus on questions about specific drug groups or health issues to better understand legislative influence on healthcare. Statistical associations between legislative focus and health outcomes, using longitudinal data and appropriate modeling techniques to assess potential policy impact, can be explored.

## Supplementary Information

Supplementary Material 1.

Supplementary Material 2.

Supplementary Material 3.

## Data Availability

The data associated with this article are presented in the manuscript and supplementary material. The data were obtained from the website of Parliament of India, which can be accessed at https://sansad.in/ls.

## Abbreviations

COVID: Coronavirus disease
DALY: Disability-adjusted life year
LDA: Latent Dirichlet Allocation
MPs: Members of parliament
PMJAY: Pradhan Mantri Jan Arogya Yojana
