# Supplementary material for: Questions regarding medication-related issues raised by legislators in the Indian parliament, 2017–2024
Source: BMC Public Health. 2025 Sep 24;25:3119. doi: 10.1186/s12889-025-24278-8 (PMC12462045; doi:10.1186/s12889-025-24278-8)
Supplement: Supplementary file 1 — Supplementary Material 1. [file 12889_2025_24278_MOESM1_ESM.pdf]

Questions regarding medication-related issues raised by legislators in the Indian parliament, 2017–2024

Preethi J Shenoy[a], Rajeshwari Shastry[a], Ashwin Kamath[a]*

[a]Department of Pharmacology, Kasturba Medical College Mangalore, Manipal Academy of Higher Education, Manipal, India

*Corresponding Author: ashwin.kamath@manipal.edu

Additional file 1. List of keywords to identify the drug-related questions raised by the Indian legislators in the parliament

Keywords	("tablet", "tablets", "drug", "drugs", "antibiotic", "antibiotics", "generic", "medicine", "medicines", "medicinal", "pharmaceutical", "pharmaceuticals", "nutraceutical", "nutraceuticals", "supplement", "supplements", "deworming", "ors", "vaccine", "vaccines", "dose", "spurious", "vial", "vials", "syrup", "polypharmacy", "injection", "injections", "adverse event", "adverse events", "adverse drug reaction", "adverse drug reactions", "adverse effect", "adverse effects", "side effect", "side effects", "steroid", "steroids", "implant", "implants", "medical device", "medical devices", "nlem")
