# Supplementary material for: Questions regarding medication-related issues raised by legislators in the Indian parliament, 2017–2024
Source: BMC Public Health. 2025 Sep 24;25:3119. doi: 10.1186/s12889-025-24278-8 (PMC12462045; doi:10.1186/s12889-025-24278-8)
Supplement: Supplementary file 3 — Supplementary Material 3. [file 12889_2025_24278_MOESM3_ESM.pdf]

Questions regarding medication-related issues raised by legislators in the Indian parliament, 2017–2024

Preethi J Shenoy[a], Rajeshwari Shastry[a], Ashwin Kamath[a]*

[a]Department of Pharmacology, Kasturba Medical College Mangalore, Manipal Academy of Higher Education, Manipal, India

*Corresponding Author: ashwin.kamath@manipal.edu

Additional file 3. Theme and year-wise summaries of questions asked by Indian legislators in the parliament.

Theme: Cancer treatment and research			
**Lok Sabha session**	**Session start date**	**Session end date**	**Summaries of questions asked**
16LSXI	31-01-2017	12-04-2017	Cancer cases, shortage of oncologists, and government measures for affordable treatment.
16LSXI	31-01-2017	12-04-2017	Government plans for early detection and prevention of cancer.
16LSXI	31-01-2017	12-04-2017	WHO suggestions for cancer prevention and government plans for new cancer care centers.
16LSXI	31-01-2017	12-04-2017	Increase in terminal diseases like cancer and government measures for affordable treatment.
16LSXIII	15-12-2017	05-01-2018	Availability and promotion of cancer vaccines.
16LSXIII	15-12-2017	05-01-2018	Introduction of HPV vaccine under the universal immunization program to prevent cervical cancer.
16LSXIII	15-12-2017	05-01-2018	Cancer treatment and medicines out of reach for many patients, and government measures for affordable treatment.
16LSXIV	29-01-2018	06-04-2018	Decision against introducing cervical cancer vaccine in public health programs.
16LSXIV	29-01-2018	06-04-2018	Cancer patient statistics, treatment, and government steps for affordable care.
16LSXIV	29-01-2018	06-04-2018	Introduction of immunotherapy for cancer treatment and its availability across states.
16LSXV	18-07-2018	10-08-2018	Fake Anti-Cancer Drugs (FACD) rackets, government actions, and ongoing investigations.
16LSXV	18-07-2018	10-08-2018	Deal between India and China to reduce tariffs on Indian medicines, including anti-cancer drugs.
16LSXV	18-07-2018	10-08-2018	Rise in cancer cases and deaths, government schemes for prevention and treatment, and awareness programs.
16LSXVI	11-12-2018	08-01-2019	High cost of cancer medicines in India, government response, accessibility issues, and steps to make cancer drugs affordable and treatment free in hospitals.
16LSXVI	11-12-2018	08-01-2019	Observance of National Cancer Awareness Day, inclusion of cancer treatment under PMJAY, and affordable treatment steps.
17LSII	18-11-2019	13-12-2019	Steps to make central drug manufacturing PSUs capable of formulating cancer treatment medicines, and price comparison.
17LSIX	18-07-2022	08-08-2022	Steps to enable central PSUs to manufacture cancer treatment drugs, price comparison, and affordable treatment measures.
17LSV	29-01-2021	25-03-2021	Cervical cancer cases, proposal to include HPV vaccine, and awareness plans.
17LSVI	19-07-2021	12-08-2021	Study on cost of illness and treatment of oral cancer, steps to disseminate information, and awareness measures.
17LSVI	19-07-2021	12-08-2021	Number of cancer patients, adequacy of cancer care facilities, early detection programs, and free treatment for poor patients.

17LSVII	29-11-2021	22-12-2021	Quality enhancement of biogenerics for Acute Lymphoblastic Leukemia, and initiatives for quality-check.
17LSVII	29-11-2021	22-12-2021	Cervical cancer cases and deaths, impact of HPV vaccine, inclusion in Universal Immunization Program, and compulsory smear tests for school girls.
17LSVII	29-11-2021	22-12-2021	Cancer cases and deaths, reasons for spread, and action plan to control cancer.
17LSVIII	31-01-2022	07-04-2022	Cancer cases and deaths, treatment facilities in Leh and Kargil, and action plan to control cancer.
17LSVIII	31-01-2022	07-04-2022	Upward trend in cancer, diabetes, and HIV cases, and remedial actions taken.
17LSVIII	31-01-2022	07-04-2022	Prevalence of cancer in specific areas, increase in cancer cases, and steps to diagnose and treat cancer at affordable cost.
17LSVIII	31-01-2022	07-04-2022	Impact of COVID-19 on cancer services, steps to identify and treat cancer patients, and access to free treatment.
17LSVIII	31-01-2022	07-04-2022	Details of dedicated oncology departments in district hospitals, proposal for new oncology department in Idukki, and help for children with cancer.
17LSVIII	31-01-2022	07-04-2022	Increase in cancer incidence, common types of cancer, and steps taken to address the issue.
17LSVIII	31-01-2022	07-04-2022	Facilities provided to cancer patients under CGHS, maximum amount covered, and number of beneficiaries.
17LSVIII	31-01-2022	07-04-2022	Impact of COVID-19 on reporting of cancer and HIV cases, anticipated increase in reporting, and measures taken.
17LSX	07-12-2022	23-12-2022	Details of cancer patients, deaths, treatment facilities, and steps to make cancer treatment affordable.
17LSX	07-12-2022	23-12-2022	Use of modern technology for cancer treatment, number of cancer patients reported, and steps to provide affordable cancer treatment.
17LSX	07-12-2022	23-12-2022	India's high cancer patient count, projected rise in cancer cases, and support for free cancer treatment.
17LSX	07-12-2022	23-12-2022	Proposal to manufacture cervical cancer vaccine, and steps taken in this regard.
17LSX	07-12-2022	23-12-2022	Impact of cancer treatment costs on poverty, per capita public expenditure on cancer care, and steps to increase hospital capacity.
17LSX	07-12-2022	23-12-2022	Progress under National Programme for Prevention and Control of Cancer, Diabetes, Cardiovascular diseases and Stroke (NPPCDCS), and shortage of healthcare staff.
17LSXI	31-01-2023	06-04-2023	India's high cancer patient count, projected rise in cancer cases, and support for free cancer treatment.
17LSXI	31-01-2023	06-04-2023	Steps to boost cancer care and research in Andhra Pradesh, and availability of affordable medicines for cancer patients.
17LSXI	31-01-2023	06-04-2023	Treatment and operations for cancer patients, funds sanctioned, and data on cancer patients treated.
17LSXI	31-01-2023	06-04-2023	Establishment of affordable cancer care facilities, proton beam therapy units, and financial assistance for cancer patients in Tamil Nadu.
17LSXI	31-01-2023	06-04-2023	Development and launch of new cervical cancer vaccine, approval status, and production details.
17LSXI	31-01-2023	06-04-2023	Mass database through genome testing for predicting serious diseases, and measures for prevention of cancer and kidney disease.
17LSXI	31-01-2023	06-04-2023	Rise in breast cancer cases, mortality rate, and support for early detection and treatment of breast cancer.
17LSXI	31-01-2023	06-04-2023	Market price and demand of Ribociclib for breast cancer treatment, supply and demand, and availability under National Cancer Control Programme.
17LSXI	31-01-2023	06-04-2023	Cervical cancer cases detected, penetration of HPV vaccine, and efforts to vaccinate women.
17LSXI	31-01-2023	06-04-2023	High cancer patient count in India, financial burden on families, and special schemes for cancer prevention and treatment.
17LSXI	31-01-2023	06-04-2023	Observation of World Cancer Day, steps to achieve aims, and corrective measures for cancer treatment.

17LSXI	31-01-2023	06-04-2023	Data on women suffering from cervical cancer, awareness programs, and rollout of cervical cancer vaccines.
17LSXI	31-01-2023	06-04-2023	Increase in breast cancer cases, overall cancer cases, and steps to check the rise in cancer cases.
17LSXI	31-01-2023	06-04-2023	Approximate number of cancer patients, increase in cancer cases in Rajasthan, and steps for prevention and treatment of cancer.
17LSXI	31-01-2023	06-04-2023	Breast cancer statistics, projected increase in cases, and government response.
17LSXII	20-07-2023	11-08-2023	Addressing rising cancer cases in the North-East, collaboration opportunities, and risk factors for nasopharyngeal cancer.
17LSXII	20-07-2023	11-08-2023	Number of cancer patients, new operational guidelines for palliative care, and impact on quality of life.
17LSXII	20-07-2023	11-08-2023	Burden of outpatient treatment for cancer patients, steps to reduce out-of-pocket expenses, and impact of Ayushman Bharat PM-JAY.
17LSXII	20-07-2023	11-08-2023	Campaigns for cancer prevention, promotion of research and development, and early detection initiatives.
17LSXII	20-07-2023	11-08-2023	Assessment of cancer patient numbers, increase in cases in Rajasthan, and proposal for new cancer hospitals.
17LSXII	20-07-2023	11-08-2023	Increase in cancer cases and deaths, research conducted, and steps for prevention and regular check-ups.
17LSXII	20-07-2023	11-08-2023	Awareness campaigns for early detection and control of cancers affecting women, and details of initiatives.
17LSXII	20-07-2023	11-08-2023	High burden of cancer cases in the North-East, reasons for prevalence, and steps for prevention and early detection.
17LSXII	20-07-2023	11-08-2023	High cancer patient count in India, financial support for cancer treatment, and special schemes for early detection.
17LSXII	20-07-2023	11-08-2023	Increase in cancer cases among children, steps for awareness, prevention, and treatment.
17LSXII	20-07-2023	11-08-2023	Cancer cases and deaths, causes, and treatment facilities in Hathras district, Uttar Pradesh.
17LSXIV	04-12-2023	22-12-2023	Measures to accelerate cancer screening and treatment, role of ASHAs in promoting home-based cancer screening.
17LSXIV	04-12-2023	22-12-2023	Increase in cancer cases, reasons identified, and steps to control common types of cancer.
17LSXIV	04-12-2023	22-12-2023	Plan to tackle the increasing burden of cancer, allocation of resources, and steps to make treatment affordable.
17LSXIV	04-12-2023	22-12-2023	Projected increase in cancer cases, data on prevalence, and new initiatives to reduce cases.
17LSXV	31-01-2024	10-02-2024	Increase in cancer cases, measures to provide free medicines, and proposal for a cancer hospital in Bhagalpur.
17LSXV	31-01-2024	10-02-2024	Plan to tackle the increasing burden of cancer, allocation of resources, and steps to make treatment affordable.
17LSXV	31-01-2024	10-02-2024	Recommendation for Human Papillomavirus (HPV) vaccination, action taken, and measures to reduce cervical cancer cases.
17LSXV	31-01-2024	10-02-2024	Breast cancer statistics, mortality rate, support and subsidy for treatment, and proposal to reduce GST on breast cancer drugs.
Theme: Drug regulation and use			
**Lok Sabha session**	**Session start date**	**Session end date**	**Summaries of questions asked**
16LSXI	31-01-2017	12-04-2017	Government survey on the quality of medicines in hospitals, its findings, recommendations, and the government's response.
16LSXI	31-01-2017	12-04-2017	Government control over pharmaceutical companies, measures to ensure access to life-saving drugs, and outcomes of these measures.
16LSXI	31-01-2017	12-04-2017	Health impacts of frequent allopathic medicine use, promotion of AYUSH system, and government schemes for alternative medicine.
16LSXI	31-01-2017	12-04-2017	Proposal to amend the Drug and Cosmetics Act for mandatory barcoding of pharma products.

16LSXI	31-01-2017	12-04-2017	NITI Aayog's proposed changes to pharmaceutical and medical research approval processes, objectives, and implementation challenges.
16LSXI	31-01-2017	12-04-2017	Government's response to the nexus between corporate hospitals, pharma companies, and doctors, and plans for regulating the private medical sector.
16LSXI	31-01-2017	12-04-2017	Increase in synthetic medicine use, its adverse effects, and regulatory measures.
16LSXI	31-01-2017	12-04-2017	Hospitals equipped for drug addiction treatment and rehabilitation, and plans to expand these facilities.
16LSXI	31-01-2017	12-04-2017	Government exploration of genomics for precision medicine in non-communicable diseases.
16LSXI	31-01-2017	12-04-2017	Policy to promote bio-tech medicines, their adverse effects, and impact on generic medicines.
16LSXI	31-01-2017	12-04-2017	Issues with HIV drug production due to non-payment, and corrective steps.
16LSXI	31-01-2017	12-04-2017	Free medicines distribution under the National Health Mission, especially in remote areas.
16LSXI	31-01-2017	12-04-2017	Sale of prescription drugs without prescriptions and corrective measures.
16LSXI	31-01-2017	12-04-2017	Market monopoly by pharma companies, need for regulatory mechanisms, and government steps.
16LSXI	31-01-2017	12-04-2017	Overcharging for imported medicines and government measures to prevent it.
16LSXI	31-01-2017	12-04-2017	Guidelines for online sale of medicines and regulatory measures for e-pharmacies.
16LSXI	31-01-2017	12-04-2017	WHO identification of drug-resistant bacteria and government protection measures.
16LSXI	31-01-2017	12-04-2017	Financial compensation for clinical trial-related injuries or deaths and related guidelines.
16LSXI	31-01-2017	12-04-2017	Approved anti-diabetes drugs in the Indian market.
16LSXI	31-01-2017	12-04-2017	Joint inspection of pharma manufacturing units by the EU and related details.
16LSXI	31-01-2017	12-04-2017	Effluents from pharmaceutical industries contributing to superbugs and government regulations.
16LSXI	31-01-2017	12-04-2017	Government consideration of online sale of medicines and related monitoring mechanisms.
16LSXI	31-01-2017	12-04-2017	Ban on 344 fixed-dose combination medicines and government response.
16LSXI	31-01-2017	12-04-2017	Adverse Drug Reaction monitoring centers and government measures for drug safety.
16LSXI	31-01-2017	12-04-2017	Free medicines for BPL patients and complaints about inferior medicines.
16LSXI	31-01-2017	12-04-2017	Drug-resistant bacteria studies and government actions to curb antibiotic overuse.
16LSXI	31-01-2017	12-04-2017	Shortage of free medicines in government hospitals and details of distributed medicines.
16LSXI	31-01-2017	12-04-2017	Monitoring expired stocks of medicines and punitive actions taken.
16LSXI	31-01-2017	12-04-2017	Government preparedness for Ebola and development of drugs and vaccines.
16LSXI	31-01-2017	12-04-2017	Nexus between doctors and pharma companies affecting generic medicine prescriptions.
16LSXI	31-01-2017	12-04-2017	Inadequate drug testing laboratories and government measures to curb spurious medicines.
16LSXI	31-01-2017	12-04-2017	MGR Medical University for R&D of new medicines.
16LSXI	31-01-2017	12-04-2017	Drug resistance in newborns and government measures to address antibiotic resistance.

16LSXI	31-01-2017	12-04-2017	Shortage of HIV treatment drugs and government measures to ensure availability.
16LSXI	31-01-2017	12-04-2017	Free treatment for HIV/AIDS patients and WHO recommendations for drug access.
16LSXI	31-01-2017	12-04-2017	Clinical trials using animals in academic institutions and related regulations.
16LSXI	31-01-2017	12-04-2017	Measures to prevent over-the-counter sales of Schedule H and H1 drugs.
16LSXI	31-01-2017	12-04-2017	Problems faced by CGHS beneficiaries due to non-availability or poor quality of medicines.
16LSXI	31-01-2017	12-04-2017	Status of National Rural Health Mission and issues with essential drugs and equipment.
16LSXI	31-01-2017	12-04-2017	Excessive metal content in medicines and government monitoring measures.
16LSXI	31-01-2017	12-04-2017	Development of a new anti-malaria drug and its clinical trials.
16LSXI	31-01-2017	12-04-2017	Reports on spurious drugs and government actions against erring companies.
16LSXII	17-07-2017	11-08-2017	Import of essential drugs and government plans to minimize imports.
16LSXII	17-07-2017	11-08-2017	Regulation of online sale of drugs and government measures for e-pharmacies.
16LSXII	17-07-2017	11-08-2017	Government steps to make medicines affordable and ensure doctors prescribe generic medicines.
16LSXII	17-07-2017	11-08-2017	India's status as a major manufacturer and exporter of generic drugs.
16LSXII	17-07-2017	11-08-2017	WHO suspension of tuberculosis drugs by Svizera Labs and government response.
16LSXII	17-07-2017	11-08-2017	Mandatory bio-equivalence studies for drugs and measures to ensure quality.
16LSXII	17-07-2017	11-08-2017	Drug addiction-related suicides and government measures for rehabilitation.
16LSXII	17-07-2017	11-08-2017	Growth in nuclear medicine and radio pharmaceuticals sector.
16LSXII	17-07-2017	11-08-2017	Policy for supply of anti-venom injections and medicines in rural hospitals.
16LSXII	17-07-2017	11-08-2017	Free and affordable treatment for the poor and complaints about medicine quality.
16LSXII	17-07-2017	11-08-2017	Mandatory registration for import of medicines and details of import regulations.
16LSXII	17-07-2017	11-08-2017	Circulation of spurious, sub-standard, expired, and banned drugs, and government measures to strengthen drug regulation.
16LSXII	17-07-2017	11-08-2017	Availability of cheap medicines, schemes for free distribution, and efforts to break the nexus between pharmaceutical companies and doctors.
16LSXII	17-07-2017	11-08-2017	Stoppage of tetanus toxoid medicine production by medical companies.
16LSXII	17-07-2017	11-08-2017	Proposal to drop the Drugs and Cosmetics (Amendment) Bill and introduce new legislation for emerging health sectors.
16LSXII	17-07-2017	11-08-2017	Scheme for setting up mini drug testing laboratories to check drug quality and spurious drugs.
16LSXII	17-07-2017	11-08-2017	Increasing misleading advertisements of medicines and government actions.
16LSXIII	15-12-2017	05-01-2018	Lack of new drug development by ICMR and steps to strengthen medical research.
16LSXIII	15-12-2017	05-01-2018	Shortage of essential life-saving medicines and government measures to ensure availability.
16LSXIII	15-12-2017	05-01-2018	Increase in free medicine distribution centers for economically weaker sections.

16LSXIII	15-12-2017	05-01-2018	Impact of clinical trials and new drug introduction, and details of trials conducted.
16LSXIII	15-12-2017	05-01-2018	Steps to improve availability of essential medicines and assessment by experts.
16LSXIII	15-12-2017	05-01-2018	Sale and marketing of spurious, substandard, and expired drugs, and government actions.
16LSXIII	15-12-2017	05-01-2018	Mandatory stability tests in the pharmaceutical sector and guidelines for drug effectiveness.
16LSXIII	15-12-2017	05-01-2018	Issuance of No Objection Certificates (NOC) for importing medicines and related delays.
16LSXIII	15-12-2017	05-01-2018	Fears of drug resistance due to easy access to H1N1 medicines and government advisories.
16LSXIII	15-12-2017	05-01-2018	Cancer treatment and medicines out of reach for many patients, and government measures for affordable treatment.
16LSXIII	15-12-2017	05-01-2018	Steps to prevent over-the-counter sale of Schedule H and H1 drugs.
16LSXIII	15-12-2017	05-01-2018	Refusal of drug manufacturers to produce child doses of HIV drugs and government instructions.
16LSXIII	15-12-2017	05-01-2018	Draft notification for pharma companies to highlight generic names of drugs and related legal framework.
16LSXIV	29-01-2018	06-04-2018	Rationing of new tuberculosis medicines and availability issues.
16LSXIV	29-01-2018	06-04-2018	Rise in misleading drug advertisements and government actions.
16LSXIV	29-01-2018	06-04-2018	Distribution delays of medicines in CGHS Wellness Centres and guidelines.
16LSXIV	29-01-2018	06-04-2018	Circulation of spurious drugs in government hospitals and open markets, and government actions.
16LSXIV	29-01-2018	06-04-2018	Observations and warnings from USFDA to Indian pharma companies and government steps.
16LSXIV	29-01-2018	06-04-2018	Shortage of life-saving medical equipment and medicines in central government hospitals.
16LSXIV	29-01-2018	06-04-2018	Introduction and availability of Bedaquiline for Drug Resistant-TB.
16LSXIV	29-01-2018	06-04-2018	Transparent procurement of medicines and government measures.
16LSXIV	29-01-2018	06-04-2018	Availability of generic medical stores and guidelines for prescribing generic drugs.
16LSXIV	29-01-2018	06-04-2018	Mandatory highlighting of generic drug names on packaging.
16LSXIV	29-01-2018	06-04-2018	Production and sale of unregulated antibiotics by multinational companies and government actions.
16LSXIV	29-01-2018	06-04-2018	Uniform implementation of Drugs and Cosmetic Act provisions and National Pharmaceutical Policy (NPP), 2017.
16LSXIV	29-01-2018	06-04-2018	Availability of free medicines for various diseases in rural areas.
16LSXIV	29-01-2018	06-04-2018	Waiving clinical trials for select essential drugs approved in developed markets.
16LSXIV	29-01-2018	06-04-2018	Rise in vitiligo cases, treatment facilities, and clinical trials.
16LSXIV	29-01-2018	06-04-2018	India's status as a major manufacturer and exporter of generic medicines.
16LSXIV	29-01-2018	06-04-2018	Amendment in Drugs and Cosmetics Act for mandatory barcoding of pharmaceutical products.
16LSXIV	29-01-2018	06-04-2018	Import of Active Pharmaceutical Ingredients (APIs), ban on imports from some Chinese firms, and steps for indigenous production.
16LSXIV	29-01-2018	06-04-2018	Free supply of essential and life-saving drugs in public health care centers and monitoring mechanisms.

16LSXIV	29-01-2018	06-04-2018	Unavailability of essential generic medicines in AIIMS Delhi and corrective steps.
16LSXIV	29-01-2018	06-04-2018	Proposal to increase fees for site registration, product registration, and clinical trials, and opposition from pharma companies.
16LSXIV	29-01-2018	06-04-2018	Complaints about spurious medicines in government hospitals and actions taken.
16LSXIV	29-01-2018	06-04-2018	Establishment of AMRIT pharmacies, their objectives, and benefits to patients.
16LSXIV	29-01-2018	06-04-2018	Monitoring mechanisms for drug quality and actions taken for poor quality drugs.
16LSXV	18-07-2018	10-08-2018	Publicizing information about drugs that failed quality tests and list of such medicines.
16LSXV	18-07-2018	10-08-2018	Survey on the quality of medicines in government hospitals and steps to improve quality.
16LSXV	18-07-2018	10-08-2018	Operational Tuberculosis Centers, review committees, and plans for cash incentives and drug availability.
16LSXV	18-07-2018	10-08-2018	Proposal to establish a National Medicine Bank and involvement of pharma companies under CSR.
16LSXV	18-07-2018	10-08-2018	Rules governing clinical trials, WHO's concerns, stakeholder meetings, and transparency in trials.
16LSXV	18-07-2018	10-08-2018	Fake Anti-Cancer Drugs (FACD) rackets, government actions, and ongoing investigations.
16LSXV	18-07-2018	10-08-2018	Increase in clinical trials since 2013, guidelines, and steps for strict trials.
16LSXV	18-07-2018	10-08-2018	Insufficient supply of free medicines in government health institutions and steps to address the issue.
16LSXV	18-07-2018	10-08-2018	India's high antibiotic consumption, warnings about fluoroquinolone antibiotics, and corrective measures.
16LSXV	18-07-2018	10-08-2018	Restructuring of the National List of Essential Medicines (NLEM) to include medical devices and disposables.
16LSXV	18-07-2018	10-08-2018	Purchase of short-expiry medicines in CGHS and corrective actions.
16LSXV	18-07-2018	10-08-2018	Deal between India and China to reduce tariffs on Indian medicines, including anti-cancer drugs.
16LSXV	18-07-2018	10-08-2018	New drug Carbetocin as a replacement for oxytocin during childbirth and government plans.
16LSXV	18-07-2018	10-08-2018	Mandatory prescription of generic medicines, complaints, and steps to assure quality.
16LSXV	18-07-2018	10-08-2018	High fluoride content in toothpaste, health risks, and restrictions by the Drugs Technical Advisory Board (DTAB).
16LSXV	18-07-2018	10-08-2018	Circulation of spurious medicines, cases detected, and government mechanisms to crack down on fake drugs.
16LSXV	18-07-2018	10-08-2018	Withdrawal of permission for private production of oxytocin, misuse in dairy and vegetable industry, and punitive measures.
16LSXV	18-07-2018	10-08-2018	Drugs banned by DCGI and FDA, illegal sale of banned drugs, and government actions.
16LSXVI	11-12-2018	08-01-2019	India's lag in medicine research and development, plans to increase research pace, and funds allocated.
16LSXVI	11-12-2018	08-01-2019	Unavailability of life-saving drugs in government hospitals and CGHS dispensaries, and corrective steps.
16LSXVI	11-12-2018	08-01-2019	New list of essential diagnostics by ICMR, objectives, and price fixation.
16LSXVI	11-12-2018	08-01-2019	Deaths due to lack of life-saving oxygen and medicines, and government actions.
16LSXVI	11-12-2018	08-01-2019	Drugs banned by DCGI and FDA, illegal sale, and government measures to curb it.
16LSXVI	11-12-2018	08-01-2019	Proposal for an SMS-based service to inform patients about affordable medicine alternatives.
16LSXVI	11-12-2018	08-01-2019	Policy on antibiotic use, steps to tackle antibiotic resistance, and awareness events for medical practitioners.

16LSXVI	11-12-2018	08-01-2019	Survey on the quality of medicines in government hospitals, reasons for poor quality, and actions taken.
16LSXVI	11-12-2018	08-01-2019	Proposal to ban or regulate online pharmacies and formulation of stringent rules.
16LSXVI	11-12-2018	08-01-2019	Steps to check allurement of doctors by pharmaceutical companies and promotion of generic medicines.
16LSXVI	11-12-2018	08-01-2019	Unethical marketing practices by pharmaceutical companies, Uniform Code for Pharmaceutical Marketing Practices, and government actions.
16LSXVI	11-12-2018	08-01-2019	High cost of cancer medicines in India, government response, accessibility issues, and steps to make cancer drugs affordable and treatment free in hospitals.
16LSXVI	11-12-2018	08-01-2019	Medical services and drugs imported, policy measures to reduce import dependence, and Make in India provisions.
16LSXVI	11-12-2018	08-01-2019	Studies on the impact of clinical trials and new drugs, and approvals for trials in Karnataka.
16LSXVI	11-12-2018	08-01-2019	Reports of medicines withdrawn abroad being imported and distributed in India, and steps to check such imports.
16LSXVI	11-12-2018	08-01-2019	Adverse drug reactions associated with new anti-diabetes medicines (SGLT-2 inhibitors) and government actions.
16LSXVI	11-12-2018	08-01-2019	Machinery to detect and punish dealers of fake and spurious medicines, and actions taken.
16LSXVI	11-12-2018	08-01-2019	Companies involved in developing new medicines and medical devices, funds allocated, and PPP model plans.
16LSXVII	31-01-2019	13-02-2019	Increase in Drug Resistant Tuberculosis cases, Revised National TB Control Programme, and review plans.
16LSXVII	31-01-2019	13-02-2019	Guidelines for online sale of medicines, draft amendments, and complaints received.
16LSXVII	31-01-2019	13-02-2019	New medicine for snake bite treatment, number of victims, and efforts to reduce treatment costs.
16LSXVII	31-01-2019	13-02-2019	Steps to curb over-the-counter sale of Schedule H1 drugs and antimicrobial resistance.
16LSXVII	31-01-2019	13-02-2019	Scams in procurement of drugs under NRHM in Maharashtra and punitive actions taken.
16LSXVII	31-01-2019	13-02-2019	Rise in Multi-Drug Resistant TB cases, steps to combat antibiotic resistance, and awareness measures.
16LSXVII	31-01-2019	13-02-2019	Views on excessive antibiotic use, rise in drug resistance, and guidelines to check misuse.
16LSXVII	31-01-2019	13-02-2019	Mechanism to ensure expired medicines are kept away from hospitals and monitoring details.
17LSI	17-06-2019	06-08-2019	Availability of free medicines for various diseases in rural areas and details of medicines provided.
17LSI	17-06-2019	06-08-2019	Introduction of Comprehensive Drug Monitoring System in Andhra Pradesh to link Aadhaar with doctors and pharmacies, and potential replication in other states.
17LSI	17-06-2019	06-08-2019	Leading drug companies exploiting loopholes in the Drugs and Cosmetics Act, raids conducted, and corrective steps.
17LSI	17-06-2019	06-08-2019	Complaints about excess metal content in medicines, actions taken, and proposed policy initiatives.
17LSI	17-06-2019	06-08-2019	Research and development spending on generic medicines, quality assurance steps, and issues with branded medicines.
17LSI	17-06-2019	06-08-2019	Online and offline drug sales, growth of online sales, and plans to formalize online drug sales.
17LSI	17-06-2019	06-08-2019	India's high antibiotic consumption, rising antibiotic resistance, and government measures to regulate antibiotic use.
17LSI	17-06-2019	06-08-2019	Ill-effects of the medicine Analgin, reasons for not banning it, and plans to ban other harmful medicines.
17LSI	17-06-2019	06-08-2019	Increasing number of diabetic patients, data on CGHS patients, and availability of medicines in dispensaries.
17LSI	17-06-2019	06-08-2019	Shortage of essential life-saving medicines in hospitals, especially in rural areas, and corrective steps.

17LSI	17-06-2019	06-08-2019	Adverse drug reactions associated with new anti-diabetes medicines (SGLT-2 inhibitors), and government actions.
17LSI	17-06-2019	06-08-2019	Promotion of biologic medicines for non-communicable diseases, and separate fund allocation.
17LSI	17-06-2019	06-08-2019	Safeguards under Drugs and Cosmetic Rules for challenging government analyst reports, and retesting provisions.
17LSI	17-06-2019	06-08-2019	Free medicines for diseases in rural Madhya Pradesh, details of medicines provided, number of tests conducted, and district-wise swine flu deaths in the last two years.
17LSI	17-06-2019	06-08-2019	Non-payment of dues leading to refusal of child doses of HIV drugs, and government actions.
17LSI	17-06-2019	06-08-2019	Stringent guidelines for drug distribution practices, approval status, and consumer benefits.
17LSI	17-06-2019	06-08-2019	India's status as a major market for fake and counterfeit drugs, public health implications, and government steps.
17LSI	17-06-2019	06-08-2019	Survey on the quality of medicines in government hospitals, recommendations, and government response.
17LSI	17-06-2019	06-08-2019	Guidelines for online sale of medicines, amendments to the Drugs and Cosmetics Act, and steps to address prescription validity.
17LSI	17-06-2019	06-08-2019	Acute shortage of medicines in CGHS dispensaries, irregularities in purchase and supply, and complaints from beneficiaries.
17LSII	18-11-2019	13-12-2019	Shortage of doctors in PHCs, artificial shortage of medicines, and steps to eliminate the nexus between hospital staff and suppliers.
17LSII	18-11-2019	13-12-2019	Implementation status of National Rural Health Mission, non-availability of essential medicines, and corrective measures.
17LSII	18-11-2019	13-12-2019	Clinical trials of injectable male contraceptive by ICMR, features, regulatory approval, and awareness steps.
17LSII	18-11-2019	13-12-2019	Review of CDSCO functioning, complaints about spurious medicines, and actions taken.
17LSII	18-11-2019	13-12-2019	Stocking of anti-TB drugs in private clinics, free drug provision targets, and strategies for achieving them.
17LSII	18-11-2019	13-12-2019	Steps to make central drug manufacturing PSUs capable of formulating cancer treatment medicines, and price comparison.
17LSII	18-11-2019	13-12-2019	Report on failure of drug samples, actions against companies, and steps to check spurious drugs.
17LSII	18-11-2019	13-12-2019	Sale of medicines by unqualified online portals, financial troubles for registered chemists, and regulatory actions.
17LSII	18-11-2019	13-12-2019	Medical services and medicines imported, policy measures to reduce import dependency, and Make in India provisions.
17LSII	18-11-2019	13-12-2019	Investigation into sub-standard medicines in government hospitals, survey details, and actions taken.
17LSII	18-11-2019	13-12-2019	Proposal for bar-code system to curtail fake drugs, and implementation timeline.
17LSII	18-11-2019	13-12-2019	Drug abuse among youth, directions to Drug Controller, and preventive measures.
17LSII	18-11-2019	13-12-2019	Misleading advertisements by medicine companies, parameters for sale, and actions taken.
17LSII	18-11-2019	13-12-2019	Suspension of manufacturing licenses for unqualified Lipid/Liposomal Amphotericin B, and compliance status.
17LSII	18-11-2019	13-12-2019	Opening of more generic medical stores, promotion of generic medicines, and amendments to the Drugs and Cosmetics Act.
17LSII	18-11-2019	13-12-2019	Use of short-expiry medicines in generic medicines, cases registered, and corrective measures.
17LSII	18-11-2019	13-12-2019	Improvement of emergency medicines, inclusion in medical curriculum, and training for emergency first-aid.
17LSII	18-11-2019	13-12-2019	Counterfeit drugs market in India, efforts to eradicate counterfeit drugs, and details of actions taken.
17LSII	18-11-2019	13-12-2019	Scheme for strengthening drug regulatory system, funds allocated for Assam, and manpower status.

17LSII	18-11-2019	13-12-2019	Increase in lifestyle-related ailments, spending on drugs, and corrective measures.
17LSII	18-11-2019	13-12-2019	Adverse drug reactions associated with new anti-diabetes medicines (SGLT-2 inhibitors), and government actions.
17LSIII	31-01-2020	23-03-2020	Comprehensive mechanism to stop manufacturing and marketing of banned drugs, and actions taken.
17LSIII	31-01-2020	23-03-2020	India's global health security score, research on Coronavirus, and steps to ensure medicine supply.
17LSIII	31-01-2020	23-03-2020	Affordable drugs for BRICS countries, MoU for regulatory cooperation, and consensus on draft MoU.
17LSIII	31-01-2020	23-03-2020	Steps to prevent companies distributing counterfeit medicines from participating in public procurement.
17LSIII	31-01-2020	23-03-2020	Amendment to the Drugs and Magic Remedies (Objectionable Advertisements) Act, and proposed penalties.
17LSIII	31-01-2020	23-03-2020	Research on diseases affecting the poor, and plans for new medicines.
17LSIII	31-01-2020	23-03-2020	Proposal for a list of Over the Counter (OTC) medicines, and criteria for switching from prescription drugs.
17LSIII	31-01-2020	23-03-2020	Rules to regulate medical ethics, instances of misconduct, and steps to ensure prescription of generic medicines.
17LSIII	31-01-2020	23-03-2020	Ban on anti-diabetic drug Pioglitazone, reasons, and studies on its risks.
17LSIII	31-01-2020	23-03-2020	Medicines failing quality tests, actions against companies, and steps to ensure quality standards.
17LSIII	31-01-2020	23-03-2020	Assistance to China for Coronavirus, identification of preventive medicine, and guidelines on imports.
17LSIII	31-01-2020	23-03-2020	Measures to create new rules under the Drugs and Cosmetics Amendment Rules, 2018.
17LSIII	31-01-2020	23-03-2020	Impact of online sale of medicines, complaints received, and steps to clamp down on prohibited drugs.
17LSIII	31-01-2020	23-03-2020	Review of CDSCO functioning, complaints about spurious medicines, and measures to stop circulation of fake medicines.
17LSIII	31-01-2020	23-03-2020	Current status of online sale of medicines, complaints received, and guidelines for e-pharmacies.
17LSIII	31-01-2020	23-03-2020	Rules and guidelines for regulating advertisements of medicines, healthcare, food, and beverages, and actions taken against misleading advertisements.
17LSIII	31-01-2020	23-03-2020	Price rise of paracetamol and antibiotics due to Coronavirus outbreak, and steps to control prices.
17LSIII	31-01-2020	23-03-2020	Spurious medicines sold openly, cases reported, actions taken, and systems for checking medicine quality.
17LSIII	31-01-2020	23-03-2020	Nexus between drug companies and medical practitioners, steps to determine drug prices, and plans to cap trade margins.
17LSIII	31-01-2020	23-03-2020	Drugs under strict prescription norms, cases of selling restricted drugs without prescription, and actions taken.
17LSIV	14-09-2020	23-09-2020	Use of drugs under clinical trials for treating COVID-19 and other diseases, and details of permitted drugs.
17LSIV	14-09-2020	23-09-2020	Adverse drug reactions associated with new anti-diabetes medicines (SGLT-2 inhibitors), and government actions.
17LSIV	14-09-2020	23-09-2020	COVID-19 cases, measures taken to fight the pandemic, and details of tests, hospital beds, and drugs used.
17LSIX	18-07-2022	08-08-2022	Consumption of medicines and prescription drugs, per capita expenditure, and data on out-of-pocket expenditure.
17LSIX	18-07-2022	08-08-2022	Achievements of the National Action Plan 2017 for regulating drug use, and compliance with Schedule H1.
17LSIX	18-07-2022	08-08-2022	Approval of Corbevax as a COVID-19 booster dose, new class of artificial peptides, and antiviral drug development.
17LSIX	18-07-2022	08-08-2022	Government industries/PSUs manufacturing medicines, and percentage of imported medical equipment.

17LSIX	18-07-2022	08-08-2022	Threat of antimicrobial resistance, awareness steps, and rational use of antibiotics.
17LSIX	18-07-2022	08-08-2022	IT raids on Dolo-650 manufacturer, unethical deals, and actions taken to check such practices.
17LSIX	18-07-2022	08-08-2022	Financial assistance for HIV-AIDS patients, compensation for medicines and hospital expenses, and single window service delivery.
17LSIX	18-07-2022	08-08-2022	Shortage of Antiretroviral Therapy (ART) drugs, number of ART centers, and difficulties faced by state governments.
17LSIX	18-07-2022	08-08-2022	Spread of fake/spurious drugs, network of manufacturing and sale, and actions taken to check the issue.
17LSIX	18-07-2022	08-08-2022	Approval stages for COVID-19 treatment drugs, export plans, and collaborations for R&D.
17LSIX	18-07-2022	08-08-2022	Steps to enable central PSUs to manufacture cancer treatment drugs, price comparison, and affordable treatment measures.
17LSIX	18-07-2022	08-08-2022	Price list of drug formulations, introduction of QR codes, and online certification system for drug manufacturers.
17LSV	29-01-2021	25-03-2021	Import of medicines, provision for drug inspectors, and details of inspections.
17LSV	29-01-2021	25-03-2021	Fixed Dose Combinations (FDCs) in the market, regulatory framework, and measures to fix prices.
17LSV	29-01-2021	25-03-2021	Thalassemia patients, schemes for assistance, and regulation of drug prices.
17LSV	29-01-2021	25-03-2021	Sale of unauthorized COVID medicines, and steps to check spurious medicines.
17LSV	29-01-2021	25-03-2021	Objectives and features of CDSCO, and details of drugs approved in the last three years.
17LSV	29-01-2021	25-03-2021	Prescription of banned medicines, reasons, and steps to curb the practice.
17LSV	29-01-2021	25-03-2021	Action against false advertisements of COVID-19 prophylactic medicines.
17LSV	29-01-2021	25-03-2021	Medicines for chronic diseases in CGHS hospitals, and plans to open more CGHS Wellness Centres.
17LSV	29-01-2021	25-03-2021	Scheme for providing free medicines to poor sections of society, arrangements with states/UTs, and quality assurance of drugs.
17LSV	29-01-2021	25-03-2021	Permission for sale of Covishield vaccine in the open market, affordability for vulnerable populations, and inclusion in the list of essential medicines.
17LSV	29-01-2021	25-03-2021	Adverse drug reactions associated with SGLT-2 inhibitors, reports from states, and awareness steps for diabetic patients.
17LSV	29-01-2021	25-03-2021	Proposal to set up call centers for prescribing generic medicines, and measures to ensure doctors prescribe generic medicines.
17LSVI	19-07-2021	12-08-2021	Illegal manufacturing and marketing of opioid tablets, cases reported, and actions taken.
17LSVI	19-07-2021	12-08-2021	Arrangements for Remdesivir injections and other medicines, steps to curb laxity in COVID-19 behavior, and cases of Delta variant.
17LSVI	19-07-2021	12-08-2021	Provision of free essential drugs in hospitals, supply of generic drugs in Bihar, and measures to check over-prescription.
17LSVI	19-07-2021	12-08-2021	Inquiry against Drugs Controller General of India for failure to make COVID-19 medicines available, and actions taken.
17LSVI	19-07-2021	12-08-2021	Actions against companies manipulating COVID-19 injection production, audit proposals, and details of actions taken.
17LSVI	19-07-2021	12-08-2021	Exchange of spurious medicines, impact on COVID-19 recovery, and actions against those selling fake medicines.
17LSVI	19-07-2021	12-08-2021	Administration of Covaxin before phase 3 data publication, issues with trial rules, and use in clinical trial mode.
17LSVI	19-07-2021	12-08-2021	Policy to control modified COVID-19 variants, and steps to enhance vaccination rate.
17LSVI	19-07-2021	12-08-2021	Adverse drug reactions associated with SGLT-2 inhibitors, reports from Karnataka, and awareness steps.

17LSVI	19-07-2021	12-08-2021	Record of deaths due to lack of medicines and medical equipment, inquiry on such deaths, and actions against hoarding.
17LSVI	19-07-2021	12-08-2021	Overcharging by private hospitals, sale of life-saving drugs at high prices, and actions taken.
17LSVI	19-07-2021	12-08-2021	Discussions with pharma companies for indemnity against vaccine side effects, demands from companies, and stance on domestic manufacturers.
17LSVI	19-07-2021	12-08-2021	Shortage of oxygen, ventilators, and critical care medicines during COVID-19 surge, deaths due to shortages, and measures taken.
17LSVI	19-07-2021	12-08-2021	Cases of manufacturing and marketing banned/unapproved drugs, actions taken, and proposed mechanisms to stop such practices.
17LSVI	19-07-2021	12-08-2021	Prescription of generic drugs by doctors, compliance, and actions taken on complaints.
17LSVII	29-11-2021	22-12-2021	High cost of spinal muscular atrophy drugs, taxes levied, and steps to control prices.
17LSVII	29-11-2021	22-12-2021	Sale of spurious medicines during COVID-19 pandemic, actions taken, and steps to stop spurious drugs.
17LSVII	29-11-2021	22-12-2021	Resumption of export of COVID-19 treatment medicines, steps taken, and details thereof.
17LSVII	29-11-2021	22-12-2021	Delay in COVID-19 inoculation drive due to lack of advance agreements with foreign vaccine makers, and rollout of oral anti-COVID-19 drugs.
17LSVII	29-11-2021	22-12-2021	Weekly review of life-saving drugs availability, proposal for National Pharmaceutical Stockpile, and stockpiling in Tamil Nadu.
17LSVII	29-11-2021	22-12-2021	Production capacity of Covishield and Covaxin, approval of other manufacturers, and import permissions.
17LSVII	29-11-2021	22-12-2021	Complaints about fake medicines in government hospitals, and action taken.
17LSVIII	31-01-2022	07-04-2022	Misleading pharmaceutical advertisements, norms for drug sellers, and compliance measures.
17LSVIII	31-01-2022	07-04-2022	Reuse of expired medicines, health threats, and measures to curb the practice.
17LSVIII	31-01-2022	07-04-2022	Delay in publishing National Health Accounts Estimates, pharmaceutical expenditures, and regulation of drug prices.
17LSVIII	31-01-2022	07-04-2022	Lag in research and development of medicine, need for increased pace, and agreements with other countries.
17LSVIII	31-01-2022	07-04-2022	Prescription of generic medicines by doctors, and steps to ensure compliance.
17LSVIII	31-01-2022	07-04-2022	Rules for prescription and sale of antibiotics, excessive use during COVID-19, and steps to control it.
17LSVIII	31-01-2022	07-04-2022	Sale of spurious drugs during COVID-19, actions taken, and proposed stringent laws.
17LSVIII	31-01-2022	07-04-2022	Development of a generic version of Remdesivir, approval for compulsory licenses, export details, and actions to meet domestic demand.
17LSVIII	31-01-2022	07-04-2022	Funds allocated for Research & Development in drug discovery and manufacturing, and list of Indian drugs approved by FDA, USA, and EU.
17LSVIII	31-01-2022	07-04-2022	Steps to combat manufacturing of faulty and adulterated drugs, improving manpower in drug regulation, and data on faulty drugs.
17LSVIII	31-01-2022	07-04-2022	Administration of Covaxin in the 15-18 age group, approval status, and steps to get approval from other countries.
17LSVIII	31-01-2022	07-04-2022	Assessment of side-effects of medicines, sale of banned medicines in India, and details of such medicines.
17LSVIII	31-01-2022	07-04-2022	Black marketing of free medicines supplied to government hospitals, cases reported, and actions taken.
17LSVIII	31-01-2022	07-04-2022	Adverse drug reactions associated with SGLT-2 inhibitors, reports from Tamil Nadu, and awareness steps.
17LSVIII	31-01-2022	07-04-2022	Cases of fraud in COVID-19 vaccination and issuance of certificates, investigation of claims, and measures to prevent data tampering.

17LSX	07-12-2022	23-12-2022	Increase in Drug-resistant TB cases during COVID-19, steps taken to deal with the issue, and details of initiatives.
17LSX	07-12-2022	23-12-2022	Deaths of children after consuming contaminated cough syrup, steps to ensure quality of pharmaceutical products, and actions against offenders.
17LSX	07-12-2022	23-12-2022	Directive to prescribe generic medicines, promotion of generic medicines, and consumer awareness policy.
17LSX	07-12-2022	23-12-2022	Sale of unscientific combinations of medicines, regulatory framework, and response to the issue.
17LSX	07-12-2022	23-12-2022	Investigation of contaminated medicines reported by WHO, pharmaceutical company violations, and actions taken.
17LSX	07-12-2022	23-12-2022	Assessment of side-effects of medicines, sale of banned medicines in India, and details of such medicines.
17LSX	07-12-2022	23-12-2022	Rise in dog bite incidents, stringent guidelines to curb stray dog menace, and National Action Plan for Dog Mediated Rabies Elimination.
17LSX	07-12-2022	23-12-2022	Research on treatment for rare genetic disorders, promotion of domestic manufacturing of medicines, and outcomes achieved.
17LSX	07-12-2022	23-12-2022	Increase in drug addiction cases, reasons, and steps taken to check the increase.
17LSX	07-12-2022	23-12-2022	Number of Primary Health Centres (PHCs) in rural areas, shortage of medicines, and steps to improve PHC functioning.
17LSX	07-12-2022	23-12-2022	Increase in drug prices, medicine accessibility in rural India, and availability of branded drugs under AMRIT pharmacies.
17LSX	07-12-2022	23-12-2022	Criteria for fixing prices of essential drugs, impact on drug prices, and establishment of Jan Aushadhi Kendras.
17LSXI	31-01-2023	06-04-2023	Rise in lifestyle and rare genetic diseases, awareness measures, and steps to reduce the cost of medicines.
17LSXI	31-01-2023	06-04-2023	Failure of 67 medicine batches in random drug sample tests, measures to control substandard medicines, and penal actions taken.
17LSXI	31-01-2023	06-04-2023	Increase in drug prices, medicine accessibility in rural India, and issues with AMRIT pharmacies and pharmacist-to-patient ratio.
17LSXI	31-01-2023	06-04-2023	Adverse events and deaths due to contaminated injection Propofol, steps taken, and variation in drug testing reports.
17LSXI	31-01-2023	06-04-2023	Collaboration with states to improve drug regulatory system, details of incidents in Uzbekistan and Gambia, and steps taken.
17LSXI	31-01-2023	06-04-2023	Steps to boost cancer care and research in Andhra Pradesh, and availability of affordable medicines for cancer patients.
17LSXI	31-01-2023	06-04-2023	Price and import of injection for Spinal Muscular Atrophy (SMA), plans for domestic manufacturing, and GST waiver proposal.
17LSXI	31-01-2023	06-04-2023	Inappropriate use of antibiotics, measures to regulate antibiotic use, and introduction of advanced diagnostic tools.
17LSXI	31-01-2023	06-04-2023	Medicines found harmful to health, and steps to prevent incidents similar to those in Gambia and Uzbekistan.
17LSXI	31-01-2023	06-04-2023	Better drug treatment for severe scrub typhus, primary risk to agricultural laborers, and effectiveness of combination antibiotic therapy.
17LSXI	31-01-2023	06-04-2023	Progress under National Health Policy 2017, quality of medicines, infrastructure development, and linking AYUSH systems to ASHA network.
17LSXI	31-01-2023	06-04-2023	New rules for registration of patented pharmaceutical products, enforcement mechanism, and issues with incomplete data reporting.
17LSXI	31-01-2023	06-04-2023	Online sale of medicines, companies involved, and measures to control quality of online medicines.
17LSXI	31-01-2023	06-04-2023	WHO alerts on Indian products, inspections undertaken, and results of inspections.
17LSXI	31-01-2023	06-04-2023	Action against drug manufacturers involved in recent controversies, centralization of drug regulation, and harmonization with international standards.
17LSXI	31-01-2023	06-04-2023	Support for kidney patients on dialysis, dialysis facilities at PHCs, and affordable pricing of medicines for kidney-transplant patients.

17LSXI	31-01-2023	06-04-2023	Directive to prescribe generic medicines, promotion of generic medicines, and policy for consumers and medical stores.
17LSXI	31-01-2023	06-04-2023	Instances of drugs failing quality-control tests, and record-keeping by inspectors and State Drug Regulatory Authorities (SDRAs).
17LSXI	31-01-2023	06-04-2023	Availability of antibiotics over the counter, ICMR advisory on antibiotic use, and steps to prevent antimicrobial resistance.
17LSXI	31-01-2023	06-04-2023	Non-compliance of medical stores with drug control norms, and corrective steps taken to address the issue.
17LSXI	31-01-2023	06-04-2023	Misuse of steroids in products, absence of regulatory mechanisms, and steps to ban OTC sale of such products.
17LSXI	31-01-2023	06-04-2023	Free Drugs and Diagnostics Service initiative, study on efficacy, and measures to increase outpatient count in Health and Wellness Centres.
17LSXI	31-01-2023	06-04-2023	Sale of spurious drugs, availability of fake drugs for serious diseases, and steps to ban spurious drugs.
17LSXI	31-01-2023	06-04-2023	Steps to prevent misuse of online medical apps, seizure of fake medicinal drugs, and deaths due to fake drugs.
17LSXI	31-01-2023	06-04-2023	Deaths due to snake bites, steps to reduce mortality rate, and production capacity of anti-venom medicines.
17LSXI	31-01-2023	06-04-2023	Achievements under the National Action Plan 2017 for regulating drug use, and compliance with Schedule H1 drugs.
17LSXI	31-01-2023	06-04-2023	Exclusion/inclusion of drugs in the National List of Essential Medicines (NLEM), and awareness regarding Antimicrobial Resistance (AMR).
17LSXII	20-07-2023	11-08-2023	Regulation of essential drug prices, budget allocation for the Department of Health Research, and plans for stricter price control.
17LSXII	20-07-2023	11-08-2023	Contaminated cough syrup found in Marshall Islands and Micronesia, and concerns about drug-resistant bacteria linked to eye drops.
17LSXII	20-07-2023	11-08-2023	Provision of free medicines in government hospitals, complaints about quality, and actions taken.
17LSXII	20-07-2023	11-08-2023	Proposal to set up Pharmacovigilance Programme of India (PvPI) centers, number of centers, and identified locations.
17LSXII	20-07-2023	11-08-2023	Promotion of quality generic medicines, inclusion of more medicines in price control, and steps to provide affordable medicines.
17LSXII	20-07-2023	11-08-2023	Issues with CGHS Wellness Centres, guidelines for prescribing medicines, and steps for monitoring and wellbeing of CGHS cardholders.
17LSXII	20-07-2023	11-08-2023	Plans to reduce prices of antibiotics and medicines for chronic diseases, and regulation of specialist practices.
17LSXII	20-07-2023	11-08-2023	Misleading advertisements of drug companies, criteria for drug sellers, and steps for compliance.
17LSXII	20-07-2023	11-08-2023	Ban on certain combination drugs, list of banned drugs, and reasons for the ban.
17LSXII	20-07-2023	11-08-2023	Availability of generic medicines in government hospitals, directives for prescribing generic medicines, and steps to ensure quality.
17LSXII	20-07-2023	11-08-2023	Survey on spurious medicines, policy for regulation, and action taken to address the issue.
17LSXII	20-07-2023	11-08-2023	Awareness of Monkey-Pox infection, number of cases detected, and availability of drugs.
17LSXII	20-07-2023	11-08-2023	Increase in dengue fever cases in Kerala, report from state government, and issues with over-the-counter medicines.
17LSXII	20-07-2023	11-08-2023	Issues with prescribing generic medicines in government hospitals, advisory issued, and survey proposal.
17LSXII	20-07-2023	11-08-2023	Plan to increase access to generic medicines, steps taken, and measures to ensure quality and safety.
17LSXII	20-07-2023	11-08-2023	Sale of medicines without prescription, and steps to regulate over-the-counter sale.
17LSXII	20-07-2023	11-08-2023	Actions to control illegitimate drugs in the market, and impact of deaths in Africa on Indian medicine exports.

17LSXII	20-07-2023	11-08-2023	Reports of toxins in cough syrups exported to Uzbekistan and Gambia, investigation into manufacturing standards, and global alert proposal.
17LSXII	20-07-2023	11-08-2023	Harm caused by Indian medicines globally, steps taken to address the issue, and details of manufacturers.
17LSXII	20-07-2023	11-08-2023	Deaths due to unregulated use of Indian medicines, steps taken to investigate, and details of actions.
17LSXII	20-07-2023	11-08-2023	Prevalence of polypharmacy among the elderly, measures to create awareness, and studies on its impact.
17LSXII	20-07-2023	11-08-2023	Sale of spurious medicines, cases unearthed, deaths reported, and monitoring mechanisms.
17LSXII	20-07-2023	11-08-2023	Anaemia cases among women, measures taken, and medicines provided under Anaemia Mukt Bharat in Andhra Pradesh.
17LSXII	20-07-2023	11-08-2023	Rise in sales of Dolo tablets during COVID-19, guidelines for consumption, and regulation of drug promotion.
17LSXII	20-07-2023	11-08-2023	Rising prices of life-saving medicines, steps to control prices, and details of measures taken.
17LSXII	20-07-2023	11-08-2023	Amendment of rules to stop adulterated drugs, steps to control sub-standard drug production.
17LSXIV	04-12-2023	22-12-2023	Rising number of Tuberculosis (TB) cases, challenges in procuring TB medicines, and support for state institutions.
17LSXIV	04-12-2023	22-12-2023	Issues in drug testing laboratories, steps to increase laboratories, and proposal for a Central Drug Testing Laboratory in Andhra Pradesh.
17LSXIV	04-12-2023	22-12-2023	Timeline for clinical trial approvals, steps to provide predictable regulations, and comparison with advanced economies.
17LSXIV	04-12-2023	22-12-2023	Provision of free insulin vials and other medicines at AIIMS, Delhi, and proposal to extend to other central government hospitals.
17LSXIV	04-12-2023	22-12-2023	Streamlining supply of medicines at CGHS Wellness Centres, issues with local chemists, and steps taken in North East.
17LSXIV	04-12-2023	22-12-2023	Progress of Ayushman Bharat scheme, availability of essential medicines, and budget for vector-borne diseases.
17LSXIV	04-12-2023	22-12-2023	Shortage of tuberculosis drugs, interruptions in supply, and permissions for local purchases.
17LSXIV	04-12-2023	22-12-2023	Stockout of key MDR-TB drugs (Linezolid, Clofazimine, Cycloserine) in Kerala, delays in receiving these drugs, interruptions in supply, and last-minute permissions for local purchases by the Central TB Division.
17LSXIV	04-12-2023	22-12-2023	Registered cases of spurious medicinal products, steps to maintain quality standards, and expenditure on Pradhan Mantri Swasthya Suraksha Yojana.
17LSXIV	04-12-2023	22-12-2023	Timeline for processing applications at CDSCO, issues faced by companies, and steps to resolve them.
17LSXV	31-01-2024	10-02-2024	Ban on unapproved Fixed Dose Combinations (FDCs) of antibiotics, prosecution of drug manufacturers, and details of actions taken.
17LSXV	31-01-2024	10-02-2024	Increase in cancer cases, measures to provide free medicines, and proposal for a cancer hospital in Bhagalpur.
17LSXV	31-01-2024	10-02-2024	High number of persons with disabilities related to leprosy, expansion of telemedicine initiative, and supply of medicines.
17LSXV	31-01-2024	10-02-2024	Promotion of AMRIT pharmacies in tribal areas, availability of modern and AYUSH medicines, and skilled health professionals.
17LSXV	31-01-2024	10-02-2024	Breast cancer statistics, mortality rate, support and subsidy for treatment, and proposal to reduce GST on breast cancer drugs.
17LSXV	31-01-2024	10-02-2024	Review of essential/generic medicines under CGHS, and reasons for outdated prescribed medicines.
17LSXV	31-01-2024	10-02-2024	Warning labels for common cold drug fixed dose combinations, and decision by the Subject Expert Committee.
17LSXV	31-01-2024	10-02-2024	Measures to ensure effective implementation of WHO standards for pharmaceuticals, and monitoring mechanism for supply chains.
17LSXV	31-01-2024	10-02-2024	Amendment of rules for manufacturers and suppliers to curb spurious medicines, and steps to control production.

17LSXV	31-01-2024	10-02-2024	Proposal for a cadre-based structure of officers for pharmaceutical issues, and reasons for not establishing such a cadre.
17LSXV	31-01-2024	10-02-2024	Revised rules under Schedule M of the Drugs and Cosmetics Rules, 1945, and deadlines for WHO Good Manufacturing Practices certification.
17LSXV	31-01-2024	10-02-2024	Harm caused by eye ailments related medicines, investigation initiated, and action taken.
Theme: General health issues			
**Lok Sabha session**	**Session start date**	**Session end date**	**Summaries of questions asked**
16LSXI	31-01-2017	12-04-2017	Government initiatives and policies for health sector development, spending on health schemes, and involvement of NGOs in health programs.
16LSXI	31-01-2017	12-04-2017	Free medical treatment for senior citizens in developed countries and government plans for India.
16LSXI	31-01-2017	12-04-2017	Approval and impact assessment of Depo-Provera contraceptive, and regulation of contraceptive pills.
16LSXI	31-01-2017	12-04-2017	Health infrastructure disparity between rural and urban areas, and government steps to address it.
16LSXI	31-01-2017	12-04-2017	Budget allocation and expenditure for family planning under the National Health Mission.
16LSXI	31-01-2017	12-04-2017	Details of Deendayal Amrit Yojana and its implementation.
16LSXI	31-01-2017	12-04-2017	Phase-II and Phase-III of Mission Indradhanush, funds allocation, and vaccination achievements.
16LSXI	31-01-2017	12-04-2017	National Deworming Day details and government steps to achieve its objectives.
16LSXI	31-01-2017	12-04-2017	Collaboration with Australia for dengue control using bacteria and related details.
16LSXI	31-01-2017	12-04-2017	Vector-borne diseases statistics, government measures, and financial assistance.
16LSXI	31-01-2017	12-04-2017	Strengthening laboratory testing of meat products and measures to check antibiotic use.
16LSXI	31-01-2017	12-04-2017	Government preparedness for epidemics and measures for early detection and treatment.
16LSXI	31-01-2017	12-04-2017	Re-registration norms for medical practitioners and government steps to enhance skills.
16LSXI	31-01-2017	12-04-2017	Preventive measures for communicable diseases and WHO suggestions for R&D during epidemics.
16LSXI	31-01-2017	12-04-2017	BRICS collaboration for regulatory approvals in health emergencies.
16LSXI	31-01-2017	12-04-2017	Increase in antibiotic resistance and government measures to regulate antibiotic use.
16LSXI	31-01-2017	12-04-2017	Setting up of Medical Technology Assessment Board for regulating medical devices.
16LSXI	31-01-2017	12-04-2017	Health program funding in Kerala and details of fund utilization.
16LSXII	17-07-2017	11-08-2017	Antibiotic use in poultry causing resistance in humans and government regulatory measures.
16LSXII	17-07-2017	11-08-2017	Reappearance of measles and government steps to address it.
16LSXII	17-07-2017	11-08-2017	Hepatitis patient statistics and government measures for prevention and treatment.
16LSXII	17-07-2017	11-08-2017	Monitoring activities under Jansankhya Sthirata Kosh and population control program outcomes.
16LSXII	17-07-2017	11-08-2017	High percentage of youth with anemia, government steps to reduce iron deficiency.
16LSXII	17-07-2017	11-08-2017	Complaints of uneasiness from deworming tablets, investigations, and future preventive actions.

16LSXII	17-07-2017	11-08-2017	Antara Programme (injectable contraceptive) incentives for ASHA workers and details of beneficiaries.
16LSXII	17-07-2017	11-08-2017	Vector-borne diseases and H1N1 cases and deaths, government measures, and proposed national programs.
16LSXII	17-07-2017	11-08-2017	Introduction of injectable contraceptives in the National Family Planning Programme and concerns about adverse effects.
16LSXII	17-07-2017	11-08-2017	Indiscriminate use of old injectable antibiotics, lack of new antibiotic research, and government measures.
16LSXIII	15-12-2017	05-01-2018	Approval and features of the National Health Policy, 2017, and steps to increase public health expenditure.
16LSXIII	15-12-2017	05-01-2018	Waterborne diseases, reasons, and government corrective measures.
16LSXIII	15-12-2017	05-01-2018	Program for controlling/eradicating Filariasis and progress made.
16LSXIII	15-12-2017	05-01-2018	Rise in vector-borne diseases, government measures, and surveillance mechanisms.
16LSXIII	15-12-2017	05-01-2018	Swine flu cases and deaths, government preparedness, and major schemes.
16LSXIII	15-12-2017	05-01-2018	Malnutrition as a major cause of death and government steps to address it.
16LSXIII	15-12-2017	05-01-2018	National Nutrition Strategy and initiatives to prevent malnutrition.
16LSXIV	29-01-2018	06-04-2018	Anemia and mineral deficiencies in rural and tribal areas, and government schemes.
16LSXIV	29-01-2018	06-04-2018	Illegal use of Oxytocin in milch cattle and government considerations for a ban.
16LSXIV	29-01-2018	06-04-2018	Status of National Rural Health Mission (NRHM) and corrective measures.
16LSXIV	29-01-2018	06-04-2018	Production and sale of unregulated antibiotics by multinational companies and government actions.
16LSXIV	29-01-2018	06-04-2018	Scheme for nutritional support and skill training for out-of-school girls.
16LSXIV	29-01-2018	06-04-2018	Research in medical marijuana and related projects.
16LSXIV	29-01-2018	06-04-2018	Completion and achievements of Mission Indradhanush Phase-II and plans for Phase-III.
16LSXV	18-07-2018	10-08-2018	Threat of bird flu, lab tests for meat products, and steps to prevent health problems from antibiotics in food.
16LSXV	18-07-2018	10-08-2018	Central assistance for endosulfan victims in Kasaragod District and financial support details.
16LSXV	18-07-2018	10-08-2018	Steps to eliminate Lymphatic Filariasis (LF), strategies, and awareness measures.
16LSXV	18-07-2018	10-08-2018	Restrictions on the sale of oxytocin, guidelines, and monitoring task force.
16LSXV	18-07-2018	10-08-2018	Double burden of undernutrition and obesity, affected groups, and government plans.
16LSXV	18-07-2018	10-08-2018	Population control schemes, expenditure, and World Bank funds.
16LSXV	18-07-2018	10-08-2018	Vitamin D deficiency among Indian women, its impact, and government proposals.
16LSXV	18-07-2018	10-08-2018	Consideration of a ban on E-cigarettes or Electronic Nicotine Delivery Systems (ENDS) and related recommendations.
16LSXV	18-07-2018	10-08-2018	Rise in diseases caused by arsenic pollution, government steps, and foreign assistance.
16LSXVI	11-12-2018	08-01-2019	Prevalence of communicable diseases, government initiatives, and funds allocated.
16LSXVI	11-12-2018	08-01-2019	Addictiveness and health risks of E-cigarettes, government advisory, and state responses.
16LSXVI	11-12-2018	08-01-2019	Objectives and features of the AMRIT scheme, number of stores, and price reduction.

16LSXVI	11-12-2018	08-01-2019	High mortality rate due to superbugs, government steps to contain deaths, and public awareness.
17LSI	17-06-2019	06-08-2019	Failure to achieve National Family Welfare Programme objectives, current family planning policies, and funds allocated.
17LSI	17-06-2019	06-08-2019	Availability and cost of treatment for Morquio A syndrome, and government plans for financial assistance.
17LSI	17-06-2019	06-08-2019	India's projected population growth, actions on National Population Policy, and steps to control population.
17LSI	17-06-2019	06-08-2019	Cases and deaths due to vector-borne diseases, national program details, and funds allocated.
17LSII	18-11-2019	13-12-2019	Status of Jansankhya Sthirata Kosh, utilization of funds, and steps to stabilize population.
17LSII	18-11-2019	13-12-2019	Health issues due to anemia and mineral deficiencies, schemes implemented, and corrective measures.
17LSII	18-11-2019	13-12-2019	Family planning programs, expenditure on advertisements, and progress in reducing birthrate.
17LSII	18-11-2019	13-12-2019	Progress under Anaemia Mukt Bharat, prevalence among adolescent girls, and steps taken.
17LSII	18-11-2019	13-12-2019	Deaths due to snake bites, reasons for high numbers, and steps to ensure availability of anti-venom.
17LSII	18-11-2019	13-12-2019	Population control schemes, action plans, and potential law for population control.
17LSII	18-11-2019	13-12-2019	Allocations and expenditures for family planning activities, and supply of contraceptives to unmarried adolescents.
17LSIII	31-01-2020	23-03-2020	Committee for monitoring Japanese Encephalitis, shortcomings found, and measures taken.
17LSIII	31-01-2020	23-03-2020	Increase in snake bite cases, measures taken, and national task force for research on snake bites.
17LSIII	31-01-2020	23-03-2020	High burden of snake bite deaths and injuries, steps to address issues, and national strategy for snake bite envenoming.
17LSIII	31-01-2020	23-03-2020	Family Planning 2020 commitments, progress on adolescent birth rate, and plans to reduce it further.
17LSIII	31-01-2020	23-03-2020	Steps to check increasing population, state-wise details, and reasons for not taking action.
17LSIX	18-07-2022	08-08-2022	Initiatives under National Health Mission, complaints of irregularities, and actions taken.
17LSIX	18-07-2022	08-08-2022	Review of National Nutritional Anaemia Prophylaxis Programme, and details of the review.
17LSV	29-01-2021	25-03-2021	Increase in muscular dystrophy cases, treatment availability, and details of patients seeking treatment abroad.
17LSV	29-01-2021	25-03-2021	TB cases reported, impact of COVID-19 on TB patients, and measures to ensure treatment.
17LSVI	19-07-2021	12-08-2021	Financial resources required for vaccinating youth and middle-aged population, issues with universal vaccination, and corrective steps.
17LSVI	19-07-2021	12-08-2021	Proposal from Chhattisgarh for managing the third wave of COVID-19, and assistance provided.
17LSVI	19-07-2021	12-08-2021	Illegal manufacturing and marketing of opioid tablets, cases reported, and actions taken.
17LSVI	19-07-2021	12-08-2021	Evidence of airborne transmission of coronavirus, public health measures, and assessment of the third wave.
17LSVI	19-07-2021	12-08-2021	Increase in asthma and respiratory diseases, reasons, and steps for treatment and awareness.
17LSVI	19-07-2021	12-08-2021	Number of COVID-19 patients, proactive actions for protection, and state-wise details.
17LSVI	19-07-2021	12-08-2021	Precautionary steps for global pandemics, assessment of COVID-19 handling, and collaborative policy with states.
17LSVI	19-07-2021	12-08-2021	States still affected by the second wave of COVID-19, steps taken, and vaccination status.

17LSVI	19-07-2021	12-08-2021	Funds requested and allocated for battling the pandemic, and expenditure share borne by the Centre.
17LSVI	19-07-2021	12-08-2021	Measures for prevention of Hepatitis, availability of treatment, and state-wise details.
17LSVI	19-07-2021	12-08-2021	Deaths due to King Cobra bites, facilities for developing antivenom, and compensation for victims' families.
17LSVI	19-07-2021	12-08-2021	Proposal for Population Control Bill, actions by state governments, and Union Government's stance.
17LSVI	19-07-2021	12-08-2021	Population growth rate, projections for India becoming the most populous country, and steps for population control.
17LSVI	19-07-2021	12-08-2021	Disease eradication programs, targets and funds allocated, and role of states and NGOs.
17LSVI	19-07-2021	12-08-2021	India's burden of neglected tropical diseases, steps to reduce the burden, and public-private partnerships.
17LSVI	19-07-2021	12-08-2021	Deaths due to COVID-19, lack of oxygen supply, and compensation for victims.
17LSVI	19-07-2021	12-08-2021	Rising antimicrobial resistance, steps taken, and One Health concept.
17LSVI	19-07-2021	12-08-2021	Special benefits for health workers, details of schemes rolled out, and beneficiaries of these schemes.
17LSVI	19-07-2021	12-08-2021	Measures for prevention of Hepatitis B, availability of effective treatment, and state-wise details.
17LSVI	19-07-2021	12-08-2021	Deaths due to COVID-19, severity of the second wave, and assistance provided to states.
17LSVI	19-07-2021	12-08-2021	Steps to minimize congenital disabilities, reasons for high incidence in rural areas, and extension of Rubella vaccination.
17LSVI	19-07-2021	12-08-2021	Health crises due to population increase, steps to control population, and impact of illegal residents on health resources.
17LSVI	19-07-2021	12-08-2021	National Health Policy for Rare Diseases, suggestions received, recognized rare diseases, and assistance for low-income groups.
17LSVI	19-07-2021	12-08-2021	Implementation of National Health Mission (NHM), funds allocated and utilized, and evaluation of proper fund utilization.
17LSVI	19-07-2021	12-08-2021	Arrival of new COVID-19 variants, potential third wave, and government preparedness.
17LSVII	29-11-2021	22-12-2021	High prevalence of anemia, estimated affected population, and implementation of Anemia Mukt Bharat.
17LSVII	29-11-2021	22-12-2021	Measures for protection from Hepatitis B, available treatments, and details thereof.
17LSVII	29-11-2021	22-12-2021	Family planning initiatives, programs launched, and expenditure incurred.
17LSVII	29-11-2021	22-12-2021	Promotion of research and development for epilepsy treatment, and details thereof.
17LSVII	29-11-2021	22-12-2021	Reduction of tuberculosis incidence and death rate, impact of COVID-19, and strategies to reduce TB morbidity and mortality.
17LSVII	29-11-2021	22-12-2021	Shortage of life-saving iron chelation injection for thalassemia patients, and steps taken to address it.
17LSVII	29-11-2021	22-12-2021	Research and funds utilized by ICMR on pandemic diseases, medicinal formulations developed, and financial benefits derived.
17LSVII	29-11-2021	22-12-2021	Population growth rate, projections, and steps taken to control population growth.
17LSVII	29-11-2021	22-12-2021	Cases of Black Fungus (Mucormycosis), steps taken to tackle it, measures for controlling dengue, and special packages for treatment.
17LSVII	29-11-2021	22-12-2021	Achievement of COVID-19 vaccination targets, cumulative coverage, surge in cases post-festival season, and preventive steps taken.
17LSVII	29-11-2021	22-12-2021	Family welfare schemes in tribal areas, details for Madhya Pradesh, and new initiatives for tribal population.
17LSVII	29-11-2021	22-12-2021	Vaccination of people with disabilities, priority notification, and details thereof.

17LSVIII	31-01-2022	07-04-2022	Suspected deaths due to oxygen shortage, inquiries conducted, and measures to ensure sufficient oxygen supply.
17LSVIII	31-01-2022	07-04-2022	Changes in biomarkers for anemia determination, revised goals under AMB, and funds allocated.
17LSVIII	31-01-2022	07-04-2022	Launch of Intensified Mission Indradhanush 4.0, coverage, challenges faced, and steps taken.
17LSVIII	31-01-2022	07-04-2022	Indiscriminate use of antibiotics in poultry industry, corrective measures, and safety standards.
17LSVIII	31-01-2022	07-04-2022	Kidney failure cases, availability of treatment, and steps to improve dialysis facilities.
17LSVIII	31-01-2022	07-04-2022	Strengthening VPD surveillance system, initiatives taken, and reasons for not strengthening it.
17LSVIII	31-01-2022	07-04-2022	Operational Health and Wellness Centres, establishment in aspirational districts, and provisions for mountainous districts.
17LSVIII	31-01-2022	07-04-2022	Increase in anemia among women, reasons, and steps taken to address it.
17LSVIII	31-01-2022	07-04-2022	Proposal to waive off the seven days mandatory quarantine for expats from low-risk countries.
17LSVIII	31-01-2022	07-04-2022	Genome sequencing for Omicron variant detection, official data representation, booster doses, and steps taken.
17LSVIII	31-01-2022	07-04-2022	Assessment of medical debts accrued due to COVID-19, policy measures to address rising medical debt, and reducing out-of-pocket medical expenditure.
17LSVIII	31-01-2022	07-04-2022	Study on COVID-19 deaths among vaccinated and unvaccinated persons, number of COVID-19 and Omicron patients, and lockdown proposals.
17LSVIII	31-01-2022	07-04-2022	Preparations to deal with the current wave of COVID-19, measures for future waves, and Standard Operating Procedures for health emergencies.
17LSVIII	31-01-2022	07-04-2022	Pathogenicity of Omicron variant, genomic variations monitoring by INSACOG, and details of Omicron cases and booster shots.
17LSVIII	31-01-2022	07-04-2022	rural areas, Infant Mortality Rate (IMR), and steps to bridge the rural-urban gap.
17LSVIII	31-01-2022	07-04-2022	Investigation or research on fluorosis, prevalence of the disease, and steps to curb its incidence.
17LSVIII	31-01-2022	07-04-2022	Major health projects and special programs in Haryana, costs, and zones requiring specific programs for chronic diseases.
17LSVIII	31-01-2022	07-04-2022	Antimicrobial Resistance (AMR) crisis, data on deaths due to AMR, and steps to contain AMR.
17LSVIII	31-01-2022	07-04-2022	Proposal for free menstrual leave for working women, free menstrual pads for students, and health schemes for women.
17LSVIII	31-01-2022	07-04-2022	Tribal Health Indicators in National Family Health Surveys, targeted interventions, and Mobile Medical Units in Himachal Pradesh.
17LSVIII	31-01-2022	07-04-2022	Guidelines for regulating stem cell research and therapy, funds allocated, and availability of molecular diagnostic facilities.
17LSVIII	31-01-2022	07-04-2022	Increase in kidney, heart, and poverty-borne diseases, steps to provide timely medical facilities, and prevent the spread of diseases.
17LSX	07-12-2022	23-12-2022	Population growth rate in urban and rural areas, action taken to check population growth, and new family planning program.
17LSX	07-12-2022	23-12-2022	Reported cases of measles, high-level teams to address the issue, and measures to eliminate measles by 2023.
17LSX	07-12-2022	23-12-2022	Rise in dog bite incidents, stringent guidelines to curb stray dog menace, and National Action Plan for Dog Mediated Rabies Elimination.
17LSX	07-12-2022	23-12-2022	India's projected population growth, new strategy for controlling population, and implementation of family planning policy.
17LSXI	31-01-2023	06-04-2023	Proposal to cover Autism and Cerebral Palsy under CGHS, and details of the proposal.
17LSXI	31-01-2023	06-04-2023	Increase in anemia among women, measures taken, and efficacy of Anemia Mukt Bharat in Odisha.

17LSXI	31-01-2023	06-04-2023	Estimate of people suffering from rare diseases, reliance on crowdfunding for financial support, and approved treatment institutes.
17LSXI	31-01-2023	06-04-2023	Steps to address cultural barriers related to reproductive health of women, and details of initiatives taken.
17LSXI	31-01-2023	06-04-2023	Funds allocated and utilized for Rashtriya Kishor Swasthya Karyakram (RKSK), and number of adolescents benefited.
17LSXI	31-01-2023	06-04-2023	Scheme for treatment of diseases related to heart, kidney, and bone for elderly persons, and details of the scheme.
17LSXI	31-01-2023	06-04-2023	Increase in asthma and respiratory diseases, reasons, and steps to provide medical treatment and awareness.
17LSXI	31-01-2023	06-04-2023	Data on critical and fast-spreading diseases, research conducted, and details of preventive measures.
17LSXI	31-01-2023	06-04-2023	Number of dementia cases reported, projected increase in dementia cases, and steps to address risk factors.
17LSXI	31-01-2023	06-04-2023	Facility-based newborn care units, role of ASHA workers, and decrease in infant mortality rate.
17LSXI	31-01-2023	06-04-2023	Number of deaths due to heart attack, causes and remedies, and steps to create awareness about silent heart attacks.
17LSXI	31-01-2023	06-04-2023	India's population growth, steps to control population explosion, and reasons for not implementing measures.
17LSXI	31-01-2023	06-04-2023	Unmet need for family planning among young women, steps to provide better access to reproductive health services, and family planning programs.
17LSXI	31-01-2023	06-04-2023	Proposal for national crowdfunding portal for rare diseases, financial assistance provided, and crowdfunding under CSR.
17LSXI	31-01-2023	06-04-2023	Use of Molecular Iodine in disease prevention, proposal to develop its production, and details of its effectiveness.
17LSXI	31-01-2023	06-04-2023	CGHS facilities for employees of central autonomous bodies, reasons for not providing facilities, and financial constraints.
17LSXI	31-01-2023	06-04-2023	Implementation of Pradhan Mantri Surakshit Matritva Abhiyan in Uttar Pradesh, guidelines issued, and number of beneficiaries.
17LSXI	31-01-2023	06-04-2023	Proposal for Universal Health Coverage, steps taken, and funds spent.
17LSXII	20-07-2023	11-08-2023	Major health projects and special programs launched, costs, and status of projects in aspirational districts.
17LSXII	20-07-2023	11-08-2023	Measles cases and deaths, vaccination coverage, and campaigns conducted.
17LSXII	20-07-2023	11-08-2023	Mobile Medical Units in Ernakulam, facilities provided, expenses, and future development plans.
17LSXII	20-07-2023	11-08-2023	Tuberculosis (TB) cases and deaths, progress under Nikshay Mitra scheme, and steps to make TB elimination facilities available in remote areas.
17LSXII	20-07-2023	11-08-2023	Performance evaluation of National AIDS Control Programme (NACP) Phase-IV, targets achieved, and funds allocated.
17LSXII	20-07-2023	11-08-2023	Allocation and utilization of funds under Navjaat Shishu Suraksha Karyakram, steps for early detection of diseases, and number of beneficiaries.
17LSXII	20-07-2023	11-08-2023	Steps for population control, targets set, and reasons for not implementing measures.
17LSXII	20-07-2023	11-08-2023	Rise in dog bite incidents, guidelines to curb stray dog menace, and National Action Plan for Dog Mediated Rabies Elimination.
17LSXII	20-07-2023	11-08-2023	Proposal for a National Mission to tackle thalassemia, country-wide screening, and targets for prevention.
17LSXII	20-07-2023	11-08-2023	Achievements under National Leprosy Eradication Programme, challenges faced, and strategic roadmap for zero cases by 2030.
17LSXII	20-07-2023	11-08-2023	Number of Newborn Stabilisation Units (NBSUs) set up, role of ASHA workers, and decline in Infant Mortality Rate (IMR).
17LSXII	20-07-2023	11-08-2023	Steps for the treatment of Hepatitis B, and details of initiatives.
17LSXII	20-07-2023	11-08-2023	Funds allocated for new Family Planning Centres, targets set, and supply of contraceptives to unmarried adolescents.

17LSXII	20-07-2023	11-08-2023	High out-of-pocket (OOPE) spending on health, impact of Ayushman Bharat scheme, and plans to include outpatient treatment.
17LSXII	20-07-2023	11-08-2023	Union Health Budget as a percentage of GDP, proposal for affordable Universal Health Coverage, and details of the plan.
17LSXII	20-07-2023	11-08-2023	Impact of anemia on SC/ST/OBC communities, measures taken, and reasons for anemia.
17LSXII	20-07-2023	11-08-2023	Progress under Anaemia Mukt Bharat Programme, funds allocated, and steps to eliminate anemia.
17LSXII	20-07-2023	11-08-2023	Deadline for eradication of Tuberculosis (TB), survey at block levels, and steps for molecular testing.
17LSXII	20-07-2023	11-08-2023	Funds released for COVID-19 management under NHM, allocation for Tamil Nadu, and request for additional doses.
17LSXII	20-07-2023	11-08-2023	Participation in clinical trials for rare diseases, initiatives taken, and details of the plan.
17LSXII	20-07-2023	11-08-2023	Major health programs implemented, provision for Mobile Medical Units (MMUs), and district-wise details of MMUs.
17LSXII	20-07-2023	11-08-2023	Alternative approach to combat TB, study undertaken, and details of the plan.
17LSXII	20-07-2023	11-08-2023	Impact of socioeconomic inequalities on health outcomes, accessibility of services, and out-of-pocket expenditures.
17LSXII	20-07-2023	11-08-2023	Prevalence of Sickle Cell Disease (SCD), most affected states, and data on the disease.
17LSXII	20-07-2023	11-08-2023	Issues with CGHS services, doctor-to-population ratio, and steps to clear pending bills.
17LSXII	20-07-2023	11-08-2023	Status of Surakshit Matritva Aashwasan (SUMAN) scheme and Janani Shishu Suraksha Karyakram (JSSK) in Uttar Pradesh, and financial support provided.
17LSXII	20-07-2023	11-08-2023	Number of people affected by Hemophilia, measures for affordable treatment, and representation in government jobs and sports.
17LSXII	20-07-2023	11-08-2023	Steps for malaria treatment in tribal areas, deaths due to anemia, and funds allocated for North-Eastern States.
17LSXII	20-07-2023	11-08-2023	Review of Accredited Social Health Activists (ASHAs) contribution to health awareness, and outcomes achieved.
17LSXII	20-07-2023	11-08-2023	Increase in cardiac deaths post-COVID-19, reasons, and schemes to prevent the disease.
17LSXII	20-07-2023	11-08-2023	High preterm birth rate in India, reasons, and steps to improve neonatal infrastructure under Newborn Action Plan.
17LSXII	20-07-2023	11-08-2023	Reports of toxins in cough syrups exported to Uzbekistan and Gambia, investigation into manufacturing standards, and global alert proposal.
17LSXII	20-07-2023	11-08-2023	Health expenditure as a percentage of GDP, steps to reduce out-of-pocket expenditure, and status of AIIMS, Kozhikode.
17LSXII	20-07-2023	11-08-2023	Progress in combating Sickle Cell Anemia using CRISPR CAS-9 therapy, budget allocation, and steps to eradicate the disease.
17LSXII	20-07-2023	11-08-2023	Family planning awareness programs, funds allocated, and progress in reducing birth rate.
17LSXII	20-07-2023	11-08-2023	Funds allocated under Ayushman Bharat Scheme, number of patients benefited, and registration details.
17LSXII	20-07-2023	11-08-2023	Benefits of central health schemes for expectant women and infants, and monitoring procedures.
17LSXII	20-07-2023	11-08-2023	Proposal for an integrated health policy, integration of AYUSH systems, and response to 'Heal by India' and 'Heal in India' initiatives.
17LSXIV	04-12-2023	22-12-2023	Specialized screening centers for early detection of life-threatening diseases, and plans for timely diagnosis.
17LSXIV	04-12-2023	22-12-2023	International recognition in the fight against Tuberculosis, and steps taken over the last nine years.
17LSXIV	04-12-2023	22-12-2023	Health schemes implemented in Bihar, West Bengal, Maharashtra, Haryana, and Jharkhand, and funds allocated.
17LSXIV	04-12-2023	22-12-2023	Assistance provided to state governments for health services, shortage of funds for diagnostic facilities, and proposal for free health services.

17LSXIV	04-12-2023	22-12-2023	Coverage of diagnostic tests and doctor consultation expenses under Ayushman Bharat Yojana, and measures to ensure timely services.
17LSXIV	04-12-2023	22-12-2023	High proportion of worldwide rabies deaths from India, reasons identified, and steps to reduce cases.
17LSXIV	04-12-2023	22-12-2023	Monkeypox cases reported, steps to tackle the outbreak, and awareness measures.
17LSXIV	04-12-2023	22-12-2023	Timeline for clinical trial approvals, steps to provide predictable regulations, and comparison with advanced economies.
17LSXIV	04-12-2023	22-12-2023	Monitoring of H9N2 virus cases, steps to strengthen health infrastructure, and proposal for a monitoring unit.
17LSXIV	04-12-2023	22-12-2023	Preparation against future pandemics, financial assistance provided to states, and details of disease outbreaks.
17LSXIV	04-12-2023	22-12-2023	States awarded for best practices in health sector, schemes for women under National Health Mission, and initiatives for sickle cell disease.
17LSXIV	04-12-2023	22-12-2023	Global recovery in Tuberculosis diagnosis, challenges in implementing TB elimination programs, and progress towards elimination by 2025.
17LSXIV	04-12-2023	22-12-2023	Emphasis on pandemic preparedness in G20 New Delhi Leader s Declaration, and steps to strengthen global health architecture.
17LSXIV	04-12-2023	22-12-2023	India's high tuberculosis burden, target date for elimination, and survey on financial impact of TB.
17LSXIV	04-12-2023	22-12-2023	Implementation of National Policy for Rare Diseases, steps to ensure benefits reach patients, and details for Maharashtra.
17LSXIV	04-12-2023	22-12-2023	Health expenditure as a percentage of GDP, steps to reduce out-of-pocket expenditure, and plans to increase health spending.
17LSXIV	04-12-2023	22-12-2023	Sanjivani Clinics for free primary health facilities, proposal to open clinics in all districts, and details for Satna parliamentary constituency.
17LSXIV	04-12-2023	22-12-2023	Health indicators for Scheduled Tribes (STs) in NFHS-5, measures to enhance health conditions, and impact of wealth inequality.
17LSXIV	04-12-2023	22-12-2023	Central schemes in Madhya Pradesh, amount spent, and ongoing health projects in Bhind and Datia districts.
17LSXIV	04-12-2023	22-12-2023	Benefits of Janani Shishu Suraksha Karyakaram for SC and ST women, awareness programs, and review of implementation.
17LSXIV	04-12-2023	22-12-2023	Registered cases of spurious medicinal products, steps to maintain quality standards, and expenditure on Pradhan Mantri Swasthya Suraksha Yojana.
17LSXIV	04-12-2023	22-12-2023	Impact of socioeconomic inequalities on health outcomes, accessibility of services, and out-of-pocket expenditures.
17LSXIV	04-12-2023	22-12-2023	Impact of social connection on depression, schemes for medical illnesses, and corrective steps taken.
17LSXIV	04-12-2023	22-12-2023	Timeline for processing applications at CDSCO, issues faced by companies, and steps to resolve them.
17LSXIV	04-12-2023	22-12-2023	Processing applications at CDSCO, interaction with stakeholders, and steps to resolve issues.
17LSXV	31-01-2024	10-02-2024	Features and objectives of Kilkari Scheme and Mission Utkarsh, funds allocated, and works done in Jharkhand.
17LSXV	31-01-2024	10-02-2024	Status of Central Lactation Management Centres (CLMCs), funds allocated, and analysis of implementation.
17LSXV	31-01-2024	10-02-2024	Benchmark set by youth in medical research, proposal for a separate research cadre, and steps for prevention of Hepatitis B.
17LSXV	31-01-2024	10-02-2024	Coverage of National Rural Health Mission (NRHM), steps to improve access and strengthen public health systems, and challenges faced by states.
17LSXV	31-01-2024	10-02-2024	Plans for National Population Policy and Family Planning Policy, financial incentives for states, and population growth estimates.
17LSXV	31-01-2024	10-02-2024	Schemes for Tuberculosis treatment in tribal communities, prevalence of HIV among TB patients, and funds allocated for treatment.
17LSXV	31-01-2024	10-02-2024	Access to nutritious food, reasons for non-reduction of anemia, and steps to combat increased food costs.

17LSXV	31-01-2024	10-02-2024	Functional Ayushman Bharat Health and Wellness Centres, challenges in providing health facilities in rural areas, and steps taken.
17LSXV	31-01-2024	10-02-2024	Increase in cases of new COVID-19 variant J.N.1, measures to check the spread, and provision of medical facilities.
17LSXV	31-01-2024	10-02-2024	International recognition in the fight against Tuberculosis, and steps taken over the past nine years.
17LSXV	31-01-2024	10-02-2024	Financial assistance provided to Odisha for various schemes, number of beneficiaries, and COVID-19 vaccinations.
17LSXV	31-01-2024	10-02-2024	Community health insurance schemes to reduce out-of-pocket expenditure, and measures taken.

Theme: Health infrastructure			
**Lok Sabha session**	**Session start date**	**Session end date**	**Summaries of questions asked**
16LSXI	31-01-2017	12-04-2017	Government survey on the quality of medicines in hospitals, its findings, recommendations, and the government's response.
16LSXI	31-01-2017	12-04-2017	Differences in immunization programs between government and private hospitals, amendments to the Universal Immunization Programme, and efforts to cover all children.
16LSXI	31-01-2017	12-04-2017	Government's response to the nexus between corporate hospitals, pharma companies, and doctors, and plans for regulating the private medical sector.
16LSXI	31-01-2017	12-04-2017	Government initiatives and policies for health sector development, spending on health schemes, and involvement of NGOs in health programs.
16LSXI	31-01-2017	12-04-2017	Measures to check malnutrition among BPL and tribal populations, and the functioning of healthcare centers in these areas.
16LSXI	31-01-2017	12-04-2017	Hospitals equipped for drug addiction treatment and rehabilitation, and plans to expand these facilities.
16LSXI	31-01-2017	12-04-2017	Per capita health expenditure in India compared to developed countries, and steps to provide affordable healthcare.
16LSXI	31-01-2017	12-04-2017	Health infrastructure disparity between rural and urban areas, and government steps to address it.
16LSXI	31-01-2017	12-04-2017	Shortage of free medicines in government hospitals and details of distributed medicines.
16LSXI	31-01-2017	12-04-2017	High injection administration in government hospitals and steps to educate against it.
16LSXI	31-01-2017	12-04-2017	Upgradation of rural health centers under the India Newborn Action Plan.
16LSXI	31-01-2017	12-04-2017	Setting up of Medical Technology Assessment Board for regulating medical devices.
16LSXII	17-07-2017	11-08-2017	Policy for supply of anti-venom injections and medicines in rural hospitals.
16LSXII	17-07-2017	11-08-2017	Shortage of Inactivated Polio Vaccine (IPV) in government and private hospitals, reasons, and corrective measures.
16LSXIII	15-12-2017	05-01-2018	Focus on medical research for tropical diseases, imbalance in health facilities, and steps to improve rural healthcare.
16LSXIII	15-12-2017	05-01-2018	Policy for providing snake antivenom injections in primary health centers and rural hospitals.
16LSXIII	15-12-2017	05-01-2018	Upgradation of Primary Health Centers (PHCs) and Community Health Centers (CHCs) to cater to patient needs 24x7.
16LSXIII	15-12-2017	05-01-2018	Availability of essential vaccines in government hospitals and steps to include them.
16LSXIV	29-01-2018	06-04-2018	Circulation of spurious drugs in government hospitals and open markets, and government actions.
16LSXIV	29-01-2018	06-04-2018	Shortage of life-saving medical equipment and medicines in central government hospitals.
16LSXIV	29-01-2018	06-04-2018	Complaints about spurious medicines in government hospitals and actions taken.

16LSXV	18-07-2018	10-08-2018	Survey on the quality of medicines in government hospitals and steps to improve quality.
16LSXV	18-07-2018	10-08-2018	Policy for providing anti-venom injections in primary health centers and rural hospitals.
16LSXVI	11-12-2018	08-01-2019	Health issues during adolescence, government programs for proper healthcare.
16LSXVI	11-12-2018	08-01-2019	Unavailability of life-saving drugs in government hospitals and CGHS dispensaries, and corrective steps.
16LSXVI	11-12-2018	08-01-2019	Free diagnostic facilities for poor patients at AIIMS and implementation plans.
16LSXVI	11-12-2018	08-01-2019	Policy for providing anti-snake venom injections in primary health centers and rural hospitals.
16LSXVI	11-12-2018	08-01-2019	Survey on the quality of medicines in government hospitals, reasons for poor quality, and actions taken.
16LSXVI	11-12-2018	08-01-2019	High cost of cancer medicines in India, government response, accessibility issues, and steps to make cancer drugs affordable and treatment free in hospitals.
16LSXVII	31-01-2019	13-02-2019	Health services for vulnerable groups, rapid access to anti-retroviral therapy, and impact of decreased international funding.
16LSXVII	31-01-2019	13-02-2019	Mechanism to ensure expired medicines are kept away from hospitals and monitoring details.
17LSI	17-06-2019	06-08-2019	Inadequate facilities for diagnosis and treatment during seasonal disease outbreaks, especially in rural areas.
17LSI	17-06-2019	06-08-2019	Shortage of essential life-saving medicines in hospitals, especially in rural areas, and corrective steps.
17LSI	17-06-2019	06-08-2019	Survey on the quality of medicines in government hospitals, recommendations, and government response.
17LSII	18-11-2019	13-12-2019	Shortage of doctors in PHCs, artificial shortage of medicines, and steps to eliminate the nexus between hospital staff and suppliers.
17LSII	18-11-2019	13-12-2019	Policy for providing anti-venom injections in primary health centers, reported deaths, and remedial measures.
17LSII	18-11-2019	13-12-2019	Investigation into sub-standard medicines in government hospitals, survey details, and actions taken.
17LSIII	31-01-2020	23-03-2020	Awareness program for liver health issues, and role of health centers.
17LSIII	31-01-2020	23-03-2020	Policy for providing anti-venom injections in primary health centers and rural hospitals, reported deaths, and remedial measures.
17LSIII	31-01-2020	23-03-2020	Rules and guidelines for regulating advertisements of medicines, healthcare, food, and beverages, and actions taken against misleading advertisements.
17LSIII	31-01-2020	23-03-2020	WHO's 13 healthcare challenges for 2020, investment in new antibiotics, and awareness programs for healthcare and hygiene.
17LSIV	14-09-2020	23-09-2020	Development of coronavirus vaccine, other measures to control the pandemic, and improvement of district-level hospitals.
17LSIV	14-09-2020	23-09-2020	COVID-19 cases, measures taken to fight the pandemic, and details of tests, hospital beds, and drugs used.
17LSIX	18-07-2022	08-08-2022	Financial assistance for HIV-AIDS patients, compensation for medicines and hospital expenses, and single window service delivery.
17LSIX	18-07-2022	08-08-2022	Increase in COVID-19 cases, reasons for the increase, and steps to enhance health infrastructure.
17LSV	29-01-2021	25-03-2021	Rural healthcare workers as frontline workers, and provision of free COVID-19 vaccine.
17LSV	29-01-2021	25-03-2021	Medicines for chronic diseases in CGHS hospitals, and plans to open more CGHS Wellness Centres.
17LSV	29-01-2021	25-03-2021	Rise in Acute Encephalitis Syndrome (AES) cases, hospital infrastructure, and availability of vaccination.
17LSV	29-01-2021	25-03-2021	COVID-19 vaccination program in Tamil Nadu, adverse effects reported, and steps to bear hospitalization costs.

17LSVI	19-07-2021	12-08-2021	Complaints about private hospitals selling free COVID-19 vaccines, and actions taken.
17LSVI	19-07-2021	12-08-2021	Proportion of individuals vaccinated without CoWIN registration, measures for those without digital access or photo ID.
17LSVI	19-07-2021	12-08-2021	Provision of free essential drugs in hospitals, supply of generic drugs in Bihar, and measures to check over-prescription.
17LSVI	19-07-2021	12-08-2021	Administration of COVID-19 vaccines through private hospitals, price details, and booking of vaccination sites.
17LSVI	19-07-2021	12-08-2021	COVID hospitals in West Bengal, strategy for small villages, and requests for special teams.
17LSVI	19-07-2021	12-08-2021	India COVID-19 Emergency Response and Health System Preparedness Package: Phase-II, progress under Phase-I, and support for Hospital Management Information System (HMIS).
17LSVI	19-07-2021	12-08-2021	Disease eradication programs, targets and funds allocated, and role of states and NGOs.
17LSVI	19-07-2021	12-08-2021	50,000 crore fund for healthcare, and action plan for utilization.
17LSVI	19-07-2021	12-08-2021	Deaths due to COVID-19, lack of oxygen supply, and compensation for victims.
17LSVI	19-07-2021	12-08-2021	Plan of action for fungal infection outbreaks, shortage of Amphotericin-B, and assistance for healthcare systems.
17LSVI	19-07-2021	12-08-2021	Overcharging by private hospitals, sale of life-saving drugs at high prices, and actions taken.
17LSVI	19-07-2021	12-08-2021	Special benefits for health workers, details of schemes rolled out, and beneficiaries of these schemes.
17LSVI	19-07-2021	12-08-2021	Deaths due to COVID-19, severity of the second wave, and assistance provided to states.
17LSVI	19-07-2021	12-08-2021	Implementation of National Health Mission (NHM), funds allocated and utilized, and evaluation of proper fund utilization.
17LSVII	29-11-2021	22-12-2021	Data on vaccines procured by private hospitals, vaccine wastage, and funds spent on procurement.
17LSVII	29-11-2021	22-12-2021	Family planning initiatives, programs launched, and expenditure incurred.
17LSVII	29-11-2021	22-12-2021	Research and funds utilized by ICMR on pandemic diseases, medicinal formulations developed, and financial benefits derived.
17LSVII	29-11-2021	22-12-2021	Complaints about fake medicines in government hospitals, and action taken.
17LSVIII	31-01-2022	07-04-2022	Suspected deaths due to oxygen shortage, inquiries conducted, and measures to ensure sufficient oxygen supply.
17LSVIII	31-01-2022	07-04-2022	Changes in biomarkers for anemia determination, revised goals under AMB, and funds allocated.
17LSVIII	31-01-2022	07-04-2022	Launch of Intensified Mission Indradhanush 4.0, coverage, challenges faced, and steps taken.
17LSVIII	31-01-2022	07-04-2022	Allocation and utilization of funds for COVID-19 vaccination, and need for additional funds.
17LSVIII	31-01-2022	07-04-2022	Operational Health and Wellness Centres, establishment in aspirational districts, and provisions for mountainous districts.
17LSVIII	31-01-2022	07-04-2022	Preventing illnesses, hospitalizations, and deaths by increasing vaccine coverage for pneumonia and related complications.
17LSVIII	31-01-2022	07-04-2022	Efforts to make the health sector of global standards, schemes implemented, policy to reduce medical expenses, and development of hospitals in Bijnour.
17LSVIII	31-01-2022	07-04-2022	Performance assessment of Tandur District Hospital, financial assistance to exemplary hospitals, and fostering innovation in healthcare facilities.
17LSVIII	31-01-2022	07-04-2022	Speeding up the precautionary 3rd dose vaccination process, target for healthcare/frontline workers, and data on deaths after two doses.
17LSVIII	31-01-2022	07-04-2022	Advisory to States/UTs to revive control rooms for Omicron situation, infrastructure facilities, and assistance provided.
17LSVIII	31-01-2022	07-04-2022	Measures to minimize hospitalization and check the proliferation of Omicron variant without disrupting economic activity.

17LSVIII	31-01-2022	07-04-2022	Details and salient features of AB-PMJAY, number of beneficiaries, funds sanctioned and utilized, and awareness steps.
17LSVIII	31-01-2022	07-04-2022	Outreach strategy to promote awareness about COVID-19 vaccination, and expenditure incurred for media outreach programs.
17LSVIII	31-01-2022	07-04-2022	COVID-19 related research by ICMR, success percentage, and amount spent on research.
17LSVIII	31-01-2022	07-04-2022	Equitable, affordable, and quality healthcare under NRHM and NUHM, achievements, and steps to strengthen healthcare systems.
17LSVIII	31-01-2022	07-04-2022	Details of dedicated oncology departments in district hospitals, proposal for new oncology department in Idukki, and help for children with cancer.
17LSVIII	31-01-2022	07-04-2022	Special benefits and incentives for healthcare and frontline workers during COVID-19, and schemes implemented.
17LSVIII	31-01-2022	07-04-2022	Black marketing of free medicines supplied to government hospitals, cases reported, and actions taken.
17LSVIII	31-01-2022	07-04-2022	Major health projects and special programs in Haryana, costs, and zones requiring specific programs for chronic diseases.
17LSVIII	31-01-2022	07-04-2022	Major healthcare programs being implemented, provision of Mobile Medical Units (MMUs), and funds allocated.
17LSVIII	31-01-2022	07-04-2022	Shortage of healthcare professionals and infrastructure during COVID-19, assistance provided, and new posts created.
17LSVIII	31-01-2022	07-04-2022	Operational status of Ayushman Bharat Health and Wellness Centres (HWCs), tele-consultation services, and funds released under ECRP-II.
17LSVIII	31-01-2022	07-04-2022	Shortage of CGHS Wellness Centres in Dwarka, New Delhi, doctor-patient ratio, and steps to augment strength of doctors.
17LSVIII	31-01-2022	07-04-2022	Tribal Health Indicators in National Family Health Surveys, targeted interventions, and Mobile Medical Units in Himachal Pradesh.
17LSVIII	31-01-2022	07-04-2022	Guidelines for regulating stem cell research and therapy, funds allocated, and availability of molecular diagnostic facilities.
17LSVIII	31-01-2022	07-04-2022	Effectiveness of copper alloys against SARS-CoV-2 virus, and proposal to utilize copper in healthcare infrastructure.
17LSVIII	31-01-2022	07-04-2022	Study on extending vaccination center hours, proposal for round-the-clock vaccination, and details of the plan.
17LSVIII	31-01-2022	07-04-2022	Ayushman Bharat - Health and Wellness Centres (AB-HWCs) initiative, achievements, and proposals to upgrade CHCs/PHCs.
17LSVIII	31-01-2022	07-04-2022	Gender disparity in access to healthcare services, steps to improve access for women, and details of initiatives.
17LSVIII	31-01-2022	07-04-2022	Increase in kidney, heart, and poverty-borne diseases, steps to provide timely medical facilities, and prevent the spread of diseases.
17LSX	07-12-2022	23-12-2022	Pradhan Mantri TB Mukt Bharat Abhiyaan for eradicating TB by 2030, survey to identify TB crisis areas, and action plan.
17LSX	07-12-2022	23-12-2022	Health expenditure as a percentage of GDP, guidelines to modernize hospitals, and proposal for free medical treatment for all citizens.
17LSX	07-12-2022	23-12-2022	Impact of cancer treatment costs on poverty, per capita public expenditure on cancer care, and steps to increase hospital capacity.
17LSX	07-12-2022	23-12-2022	Progress under National Programme for Prevention and Control of Cancer, Diabetes, Cardiovascular diseases and Stroke (NPPCDCS), and shortage of healthcare staff.
17LSX	07-12-2022	23-12-2022	High TB patient count, increase in TB cases, and steps under Nikshay Mitras initiative to eliminate TB.
17LSXI	31-01-2023	06-04-2023	Proposal to cover Autism and Cerebral Palsy under CGHS, and details of the proposal.
17LSXI	31-01-2023	06-04-2023	Vaccination programs for children in Tamil Nadu, funds allocated, and vacancies in health centers.
17LSXI	31-01-2023	06-04-2023	Technical and financial support to Himachal Pradesh for public healthcare, and details of ambulance services provided.
17LSXI	31-01-2023	06-04-2023	Estimate of people suffering from rare diseases, reliance on crowdfunding for financial support, and approved treatment institutes.

17LSXI	31-01-2023	06-04-2023	Issuance of digital cards under Ayushman Bharat Yojana, survey of incurable diseases, and implementation of healthcare schemes.
17LSXI	31-01-2023	06-04-2023	Salient features of Pradhan Mantri Surakshit Matritva Abhiyan, funds released, and beneficiaries in Jharkhand.
17LSXI	31-01-2023	06-04-2023	National Viral Hepatitis Control Programme, steps to control and treat viral hepatitis, and funds allocated for Andhra Pradesh.
17LSXI	31-01-2023	06-04-2023	Survey to make medical treatment affordable, steps to set up critical care centers, and funds allocated.
17LSXI	31-01-2023	06-04-2023	Funds allocated and utilized for Rashtriya Kishor Swasthya Karyakram (RKSK), and number of adolescents benefited.
17LSXI	31-01-2023	06-04-2023	Salient features of Ayushman Bharat Pradhan Mantri Jan Arogya Yojana, number of beneficiaries, and coverage of diseases.
17LSXI	31-01-2023	06-04-2023	Assessment of healthcare infrastructure in tribal areas of Gujarat, and actions taken to improve infrastructure.
17LSXI	31-01-2023	06-04-2023	Cases of Measles and Rubella, steps to eliminate MR, and funds released for elimination.
17LSXI	31-01-2023	06-04-2023	Timeline for eliminating TB, PM TB Mukt Bharat Abhiyaan, and contributions to the initiative.
17LSXI	31-01-2023	06-04-2023	Works undertaken under National Health Mission (NHM), funds allocated, and proposals for new medical colleges in tribal areas.
17LSXI	31-01-2023	06-04-2023	Improvement in services of government hospitals, modernization of hospitals in rural areas, and details for Dadra & Nagar Haveli and Daman and Diu.
17LSXI	31-01-2023	06-04-2023	Issue of Antimicrobial Resistance (AMR), initiatives to prevent AMR, and surveillance systems in hospitals.
17LSXI	31-01-2023	06-04-2023	WHO funds for health-related development, and roadmap for global action plan for prevention and control of diseases.
17LSXI	31-01-2023	06-04-2023	Steps to ensure availability of medical facilities in remote regions, implementation of Ayushman Bharat Scheme, and issues with the scheme.
17LSXI	31-01-2023	06-04-2023	Complaints regarding CGHS services, need for cashless facility for serving employees, and proposal for separate hospitals for CGHS beneficiaries.
17LSXI	31-01-2023	06-04-2023	Supplementing state efforts in healthcare through centrally sponsored schemes, financial assistance provided, and steps to address bottlenecks.
17LSXI	31-01-2023	06-04-2023	Quality healthcare through equipped ambulances, reasons for lack of medical equipment, and steps to improve public sector ambulances.
17LSXI	31-01-2023	06-04-2023	Issues with treatment rates under Ayushman Bharat Yojana, proposal to increase rates, and measures to resolve problems faced by private hospitals.
17LSXI	31-01-2023	06-04-2023	Increase in chronic diseases, adequacy of healthcare facilities, and steps to improve healthcare.
17LSXII	20-07-2023	11-08-2023	Measures for diagnosing and treating sickle cell disease in tribal areas, awareness campaigns, and healthcare programs for women.
17LSXII	20-07-2023	11-08-2023	Provision of free medicines in government hospitals, complaints about quality, and actions taken.
17LSXII	20-07-2023	11-08-2023	Proposal to establish infertility treatment centers in government hospitals, financial assistance for Maharashtra, and process for grant of assistance.
17LSXII	20-07-2023	11-08-2023	Goals of Central Government Health Scheme (CGHS), pending dues to private hospitals, and steps to clear dues.
17LSXII	20-07-2023	11-08-2023	Deficiencies in healthcare infrastructure in rural and tribal areas, steps to strengthen the system, and plans for new medical colleges.
17LSXII	20-07-2023	11-08-2023	Funds for treatment of critical illnesses, issuance of Ayushman cards, and procedure for treatment in hospitals.
17LSXII	20-07-2023	11-08-2023	Availability of generic medicines in government hospitals, directives for prescribing generic medicines, and steps to ensure quality.
17LSXII	20-07-2023	11-08-2023	Deficiencies in healthcare infrastructure in rural and tribal areas, steps to strengthen the system, and plans for new medical colleges.

17LSXII	20-07-2023	11-08-2023	Audit of Community Health Centres (CHCs), report on negligence in hospitals, and steps to address labor room violence.
17LSXII	20-07-2023	11-08-2023	Assessment of cancer patient numbers, increase in cases in Rajasthan, and proposal for new cancer hospitals.
17LSXII	20-07-2023	11-08-2023	Access to mental healthcare in rural areas, initiatives to address stigma, and high prevalence of depression among rural women.
17LSXII	20-07-2023	11-08-2023	Issues with prescribing generic medicines in government hospitals, advisory issued, and survey proposal.
17LSXII	20-07-2023	11-08-2023	High preterm birth rate in India, reasons, and steps to improve neonatal infrastructure under Newborn Action Plan.
17LSXII	20-07-2023	11-08-2023	Mandate of HLL Lifecare Limited, measures for health check-up and treatment of laborers, and details of healthcare agencies.
17LSXIV	04-12-2023	22-12-2023	Achievements of National Rural Health Mission (NRHM), expenditure on rural healthcare, and health insurance coverage.
17LSXIV	04-12-2023	22-12-2023	Monitoring of H9N2 virus cases, steps to strengthen health infrastructure, and proposal for a monitoring unit.
17LSXIV	04-12-2023	22-12-2023	Hospitals empanelled under Ayushman Bharat-PM Jan Arogya Yojana, funds allocated, and coverage of diseases.
17LSXIV	04-12-2023	22-12-2023	Provision of free insulin vials and other medicines at AIIMS, Delhi, and proposal to extend to other central government hospitals.
17LSXIV	04-12-2023	22-12-2023	Ayushman Bhav campaign for healthcare services, Ayushman Apke Dwar 3.0 program, and status of the campaign.
17LSXIV	04-12-2023	22-12-2023	Proposal to set up medical colleges in each district, super specialty hospitals for mental diseases, and duration of medical courses.
17LSXIV	04-12-2023	22-12-2023	Ayushman Bhava scheme for healthcare accessibility, number of cards issued, and measures for the poor and downtrodden.
17LSXV	31-01-2024	10-02-2024	Increase in cancer cases, measures to provide free medicines, and proposal for a cancer hospital in Bhagalpur.
17LSXV	31-01-2024	10-02-2024	Access to healthcare services for transgender community, support under National AIDS Control Programme-IV, and mental health policies.
17LSXV	31-01-2024	10-02-2024	Steps to reduce healthcare costs, increase supply of doctors, and use of technology in medical education.
17LSXV	31-01-2024	10-02-2024	Reforms in healthcare sector using drones, steps under Pradhan Mantri Swasthya Suraksha Yojana, and accessibility of healthcare in remote areas.
17LSXV	31-01-2024	10-02-2024	Response to surge in COVID-19 cases, vaccination drives, and collaboration with healthcare professionals.
17LSXV	31-01-2024	10-02-2024	Outcome of international cooperation through G-20 in healthcare, systemic approach for resilient healthcare, and negotiations on pandemic prevention.
17LSXV	31-01-2024	10-02-2024	Plans to enhance accessibility and quality of healthcare for pregnant women and young children, and steps to improve maternal and child health outcomes.
17LSXV	31-01-2024	10-02-2024	Best practices in district hospitals, corrective measures taken, and additional Key Performance Indicators (KPIs).
17LSXV	31-01-2024	10-02-2024	Plan expenditure for nutrition of post-delivery women and children, and healthcare for poor old women.
Theme: Infectious diseases and their treatment			
**Lok Sabha session**	**Session start date**	**Session end date**	**Summaries of questions asked**
16LSXI	31-01-2017	12-04-2017	Government awareness and measures regarding rotavirus infection in children, including statistics and eradication plans.
16LSXI	31-01-2017	12-04-2017	India's high TB patient count, available treatments, and new initiatives for TB eradication;
16LSXI	31-01-2017	12-04-2017	WHO report on malaria spending, and corrective steps under the National Framework for Malaria Elimination.
16LSXI	31-01-2017	12-04-2017	Influenza-A H1N1 cases and deaths, preparedness, and government measures.

16LSXI	31-01-2017	12-04-2017	Rise in viral hepatitis infections, public health concerns, and government measures.
16LSXI	31-01-2017	12-04-2017	HIV/AIDS patient statistics, treatment, and government measures for prevention and care.
16LSXI	31-01-2017	12-04-2017	National Deworming Day details and government steps to achieve its objectives.
16LSXI	31-01-2017	12-04-2017	Collaboration with Australia for dengue control using bacteria and related details.
16LSXI	31-01-2017	12-04-2017	Vector-borne diseases statistics, government measures, and financial assistance.
16LSXI	31-01-2017	12-04-2017	Rise in TB and MDR-TB cases, government treatment plans, and success of TB control programs.
16LSXI	31-01-2017	12-04-2017	Rise in hepatitis infections and government measures for prevention and control.
16LSXI	31-01-2017	12-04-2017	Increase in antibiotic resistance and government measures to regulate antibiotic use.
16LSXII	17-07-2017	11-08-2017	Risk of soil-transmitted helminth infections in children and government deworming programs.
16LSXII	17-07-2017	11-08-2017	Antibiotic use in poultry causing resistance in humans and government regulatory measures.
16LSXII	17-07-2017	11-08-2017	Reappearance of measles and government steps to address it.
16LSXII	17-07-2017	11-08-2017	Hepatitis patient statistics and government measures for prevention and treatment.
16LSXII	17-07-2017	11-08-2017	HIV cases due to infected needles and treatment options.
16LSXII	17-07-2017	11-08-2017	Japanese Encephalitis cases, monitoring committee, and government measures.
16LSXII	17-07-2017	11-08-2017	Complaints of uneasiness from deworming tablets, investigations, and future preventive actions.
16LSXII	17-07-2017	11-08-2017	Vector-borne diseases and H1N1 cases and deaths, government measures, and proposed national programs.
16LSXII	17-07-2017	11-08-2017	Kidney failure due to UTI infections and heavy antibiotic use, and government actions.
16LSXII	17-07-2017	11-08-2017	Indiscriminate use of old injectable antibiotics, lack of new antibiotic research, and government measures.
16LSXII	17-07-2017	11-08-2017	Clinical trials involving HIV patients and related details.
16LSXIII	15-12-2017	05-01-2018	Achievements and review of the Revised National TB Control Programme (RNTCP).
16LSXIII	15-12-2017	05-01-2018	Waterborne diseases, reasons, and government corrective measures.
16LSXIII	15-12-2017	05-01-2018	Emerging infectious diseases and government measures in consultation with WHO.
16LSXIII	15-12-2017	05-01-2018	Observance of World AIDS Day, targets to end AIDS epidemic, and measures for prevention and treatment.
16LSXIII	15-12-2017	05-01-2018	National Action Plan on Antimicrobial Resistance (NAP-AMR) and its implementation.
16LSXIII	15-12-2017	05-01-2018	Program for controlling/eradicating Filariasis and progress made.
16LSXIII	15-12-2017	05-01-2018	Rashtriya Krimi Mukti Karyakram (RKMK) for soil-transmitted helminth infections and its expansion.
16LSXIII	15-12-2017	05-01-2018	Prescription audits and measures against antimicrobial resistance.
16LSXIII	15-12-2017	05-01-2018	Rise in vector-borne diseases, government measures, and surveillance mechanisms.
16LSXIII	15-12-2017	05-01-2018	Swine flu cases and deaths, government preparedness, and major schemes.
16LSXIII	15-12-2017	05-01-2018	Rise in fatal lung infections in children and government preventive measures.

16LSXIV	29-01-2018	06-04-2018	Swine flu cases and deaths, government preparedness, and sample collection centers.
16LSXIV	29-01-2018	06-04-2018	National plan for tuberculosis eradication and government measures.
16LSXV	18-07-2018	10-08-2018	Rise in hepatitis infections, government programs for treatment and prevention, and funds allocated.
16LSXV	18-07-2018	10-08-2018	Threat of bird flu, lab tests for meat products, and steps to prevent health problems from antibiotics in food.
16LSXV	18-07-2018	10-08-2018	Decrease in HIV patients, new hot spots, free medical assistance, and international assistance.
16LSXV	18-07-2018	10-08-2018	Steps to eliminate Lymphatic Filariasis (LF), strategies, and awareness measures.
16LSXV	18-07-2018	10-08-2018	Survey on TB and MDR-TB cases, national strategy to end TB by 2025, and funds allocated.
16LSXVI	11-12-2018	08-01-2019	Study on Pre-exposure prophylaxis (PrEP) for HIV/AIDS prevention, plans for subsidization, and availability.
16LSXVI	11-12-2018	08-01-2019	High prevalence of soil-transmitted helminth infections, deworming programs, and expansion plans.
16LSXVI	11-12-2018	08-01-2019	Estimated TB patients, Revised National Tuberculosis Control Programme, and scanning rounds.
16LSXVI	11-12-2018	08-01-2019	Reports of Zika Virus and Swine Flu cases, government steps, and assistance provided to affected states.
16LSXVII	31-01-2019	13-02-2019	Implementation of Supreme Court direction for daily dose treatment of TB patients and WHO recommendations.
16LSXVII	31-01-2019	13-02-2019	Health services for vulnerable groups, rapid access to anti-retroviral therapy, and impact of decreased international funding.
17LSI	17-06-2019	06-08-2019	WHO's Global TB Report, TB cases registered, review of RNTCP, and strategy for TB-free India by 2025.
17LSI	17-06-2019	06-08-2019	Type-II polio virus infection found in immunization injections, probe details, and additional immunization plans.
17LSI	17-06-2019	06-08-2019	Deworming program for children at risk of worm infection, collaboration with WHO, and expansion plans.
17LSI	17-06-2019	06-08-2019	Deaths due to Japanese Encephalitis, facilities provided, and steps to tackle the disease.
17LSI	17-06-2019	06-08-2019	Increase in rabies infections due to stray dogs, state-wise details, and preventive steps.
17LSI	17-06-2019	06-08-2019	Cases and deaths due to vector-borne diseases, national program details, and funds allocated.
17LSII	18-11-2019	13-12-2019	Steps to eliminate Lymphatic Filariasis, vaccination programs, and awareness measures.
17LSIII	31-01-2020	23-03-2020	National Filaria Prevention Programme, coverage, and reports of failure.
17LSIV	14-09-2020	23-09-2020	Government efforts to handle the coronavirus pandemic, commitment to deliver treatment, and details of efforts made.
17LSIV	14-09-2020	23-09-2020	COVID-19 cases, recoveries, and deaths in tribal regions, additional medical relief, and steps to protect tribal people.
17LSIX	18-07-2022	08-08-2022	Susceptibility of vaccines to Omicron sub-variants, risks of infectious diseases, and details of booster doses.
17LSIX	18-07-2022	08-08-2022	Steps for complete eradication of leprosy, supply of blister packs, and details of the National Leprosy Eradication Programme.
17LSIX	18-07-2022	08-08-2022	COVID-19 infection among children, vaccination status, and details of registered and vaccinated children.
17LSIX	18-07-2022	08-08-2022	COVID-19 infections after vaccination, state-wise and vaccine-wise details.
17LSIX	18-07-2022	08-08-2022	Target to eradicate filariasis by 2030, institutions working for treatment, and national program details.
17LSIX	18-07-2022	08-08-2022	Increase in COVID-19 cases, reasons for the increase, and steps to enhance health infrastructure.
17LSV	29-01-2021	25-03-2021	TB cases reported, impact of COVID-19 on TB patients, and measures to ensure treatment.

17LSVI	19-07-2021	12-08-2021	Evidence of airborne transmission of coronavirus, public health measures, and assessment of the third wave.
17LSVI	19-07-2021	12-08-2021	Rise in black fungus cases due to steroid use, state-wise details, and steps to check fungal infection.
17LSVI	19-07-2021	12-08-2021	Infectiousness of new coronavirus strains, reasons for surge in cases, and efficacy of vaccines against new strains.
17LSVI	19-07-2021	12-08-2021	Precautionary steps for global pandemics, assessment of COVID-19 handling, and collaborative policy with states.
17LSVI	19-07-2021	12-08-2021	Measures for prevention of Hepatitis, availability of treatment, and state-wise details.
17LSVI	19-07-2021	12-08-2021	Plan of action for fungal infection outbreaks, shortage of Amphotericin-B, and assistance for healthcare systems.
17LSVI	19-07-2021	12-08-2021	Measures for prevention of Hepatitis B, availability of effective treatment, and state-wise details.
17LSVII	29-11-2021	22-12-2021	Measures for protection from Hepatitis B, available treatments, and details thereof.
17LSVII	29-11-2021	22-12-2021	Reduction of tuberculosis incidence and death rate, impact of COVID-19, and strategies to reduce TB morbidity and mortality.
17LSVII	29-11-2021	22-12-2021	Cases of Black Fungus (Mucormycosis), steps taken to tackle it, measures for controlling dengue, and special packages for treatment.
17LSVII	29-11-2021	22-12-2021	Program to track and contain antimicrobial resistance (AMR), steps to stop over-the-counter antibiotics, deaths due to AMR, and measures to combat fungal infections.
17LSVIII	31-01-2022	07-04-2022	Expenditure on COVID-19 vaccination, rise in dengue cases, and plans for dengue treatment.
17LSVIII	31-01-2022	07-04-2022	Target to eliminate tuberculosis by 2025, impact of COVID-19, and steps to manage TB crisis.
17LSVIII	31-01-2022	07-04-2022	Preparations to deal with the current wave of COVID-19, measures for future waves, and Standard Operating Procedures for health emergencies.
17LSVIII	31-01-2022	07-04-2022	COVID-19 related research by ICMR, success percentage, and amount spent on research.
17LSX	07-12-2022	23-12-2022	Pradhan Mantri TB Mukt Bharat Abhiyaan for eradicating TB by 2030, survey to identify TB crisis areas, and action plan.
17LSX	07-12-2022	23-12-2022	Reported cases of measles, high-level teams to address the issue, and measures to eliminate measles by 2023.
17LSX	07-12-2022	23-12-2022	Recurrence of TB after 40 years, details of the skin TB test developed by ICMR, and focus on early detection and prevention.
17LSX	07-12-2022	23-12-2022	India's high TB patient count, reasons for growing TB and MDR-TB cases, and measures to reduce TB cases.
17LSX	07-12-2022	23-12-2022	High TB patient count, increase in TB cases, and steps under Nikshay Mitras initiative to eliminate TB.
17LSXI	31-01-2023	06-04-2023	Review of rising H3N2 influenza cases, directions to state governments, and steps to check the spread.
17LSXI	31-01-2023	06-04-2023	National Viral Hepatitis Control Programme, steps to control and treat viral hepatitis, and funds allocated for Andhra Pradesh.
17LSXI	31-01-2023	06-04-2023	Cases of H3N2 influenza, preparedness of states, and advisory against misuse of antibiotics.
17LSXI	31-01-2023	06-04-2023	Number of patients suffering from Hepatitis B, treatment availability, and steps to prevent the disease.
17LSXI	31-01-2023	06-04-2023	Cases of Measles and Rubella, steps to eliminate MR, and funds released for elimination.
17LSXI	31-01-2023	06-04-2023	Timeline for eliminating TB, PM TB Mukt Bharat Abhiyaan, and contributions to the initiative.
17LSXII	20-07-2023	11-08-2023	Tuberculosis (TB) cases and deaths, progress under Nikshay Mitra scheme, and steps to make TB elimination facilities available in remote areas.
17LSXII	20-07-2023	11-08-2023	Steps for the treatment of Hepatitis B, and details of initiatives.
17LSXII	20-07-2023	11-08-2023	Awareness of Monkey-Pox infection, number of cases detected, and availability of drugs.

17LSXII	20-07-2023	11-08-2023	Fatalities due to rabies infection from dog bites, steps to improve availability of anti-rabies vaccines, and control of stray dogs.
17LSXIV	04-12-2023	22-12-2023	Monkeypox cases reported, steps to tackle the outbreak, and awareness measures.
17LSXIV	04-12-2023	22-12-2023	Monitoring of H9N2 virus cases, steps to strengthen health infrastructure, and proposal for a monitoring unit.
17LSXIV	04-12-2023	22-12-2023	Global recovery in Tuberculosis diagnosis, challenges in implementing TB elimination programs, and progress towards elimination by 2025.
17LSXV	31-01-2024	10-02-2024	Schemes for Tuberculosis treatment in tribal communities, prevalence of HIV among TB patients, and funds allocated for treatment.
17LSXV	31-01-2024	10-02-2024	Increase in cases of new COVID-19 variant J.N.1, measures to check the spread, and provision of medical facilities.
Theme: Maternal and child health			
**Lok Sabha session**	**Session start date**	**Session end date**	**Summaries of questions asked**
16LSXI	31-01-2017	12-04-2017	Concerns about risks from the measles-rubella vaccine for pregnant women and unborn babies, and government measures to ensure their safety.
16LSXI	31-01-2017	12-04-2017	Differences in immunization programs between government and private hospitals, amendments to the Universal Immunization Programme, and efforts to cover all children.
16LSXI	31-01-2017	12-04-2017	Government awareness and measures regarding rotavirus infection in children, including statistics and eradication plans.
16LSXI	31-01-2017	12-04-2017	Approval and impact assessment of Depo-Provera contraceptive, and regulation of contraceptive pills.
16LSXI	31-01-2017	12-04-2017	High infant and maternal mortality rates, reasons, and government steps to address the issue.
16LSXI	31-01-2017	12-04-2017	Withdrawal of Lopinavir syrup for HIV children and corrective measures.
16LSXI	31-01-2017	12-04-2017	Mechanisms to reduce child undernutrition and promote safe feeding practices.
16LSXI	31-01-2017	12-04-2017	Prevalence of anemia in women and government measures to combat it.
16LSXI	31-01-2017	12-04-2017	Measles as a cause of child mortality and government vaccination campaigns.
16LSXI	31-01-2017	12-04-2017	Increase in reproductive illnesses among women and government measures.
16LSXII	17-07-2017	11-08-2017	Risk of soil-transmitted helminth infections in children and government deworming programs.
16LSXII	17-07-2017	11-08-2017	Health programs to check anemia prevalence in children and women.
16LSXII	17-07-2017	11-08-2017	Risks in Caesarian deliveries and government awareness measures.
16LSXII	17-07-2017	11-08-2017	Antara Programme (injectable contraceptive) incentives for ASHA workers and details of beneficiaries.
16LSXIII	15-12-2017	05-01-2018	Introduction of subdermal implants for long-term contraception.
16LSXIII	15-12-2017	05-01-2018	Refusal of drug manufacturers to produce child doses of HIV drugs and government instructions.
16LSXIII	15-12-2017	05-01-2018	Rise in fatal lung infections in children and government preventive measures.
16LSXIII	15-12-2017	05-01-2018	Primary causes of maternal deaths and steps to reduce them.
16LSXIII	15-12-2017	05-01-2018	Pneumonia deaths in children, economic burden, and government measures.
16LSXIV	29-01-2018	06-04-2018	Use of injectable contraceptives for women and safety measures.

16LSXIV	29-01-2018	06-04-2018	Shortage of Inactivated Polio Vaccine (IPV) and impact on children's immunity.
16LSXIV	29-01-2018	06-04-2018	High prevalence of anemia in women and government measures.
16LSXV	18-07-2018	10-08-2018	High percentage of women suffering from anemia, government steps for prevention, and corrective measures.
16LSXV	18-07-2018	10-08-2018	Vitamin D deficiency among Indian women, its impact, and government proposals.
16LSXV	18-07-2018	10-08-2018	New drug Carbetocin as a replacement for oxytocin during childbirth and government plans.
16LSXV	18-07-2018	10-08-2018	Maternal deaths, primary causes, and government measures to address anemia and reduce deaths.
16LSXV	18-07-2018	10-08-2018	UNICEF report on mortality rates, government estimates, and steps to reduce MMR, IMR, and NMR.
16LSXV	18-07-2018	10-08-2018	Increase in pneumonia deaths among children, government committees, and specific policies.
16LSXV	18-07-2018	10-08-2018	Children not getting vaccinated on time, reasons, and special drives for timely vaccination.
16LSXVI	11-12-2018	08-01-2019	Increase in maternal and infant mortality rates, reasons, and corrective steps.
16LSXVI	11-12-2018	08-01-2019	India Day event, objectives, and steps to improve reproductive and maternal health.
16LSXVI	11-12-2018	08-01-2019	Partners Forum 2018 event, participants, issues discussed, and steps for maternal and child health.
16LSXVI	11-12-2018	08-01-2019	High percentage of anemic women in reproductive age, government measures to reduce anemia.
16LSXVI	11-12-2018	08-01-2019	Elimination of mother-to-child transmission of HIV and Syphilis, ante-natal checkup facilities, and grievance redressal mechanism.
16LSXVI	11-12-2018	08-01-2019	Deaths of children due to pneumonia and diarrhea, and government steps to reduce such deaths.
17LSI	17-06-2019	06-08-2019	High prevalence of anemia among women, inclusion under Mission Indradhanush, and funds allocated.
17LSI	17-06-2019	06-08-2019	Government efforts to reduce infant deaths, special initiatives in Bundelkhand, and state-wise details.
17LSI	17-06-2019	06-08-2019	UN World Food Programme report on stunted growth in Indian children by 2032, and government measures to improve child health.
17LSI	17-06-2019	06-08-2019	Deworming program for children at risk of worm infection, collaboration with WHO, and expansion plans.
17LSI	17-06-2019	06-08-2019	High percentage of anemic women, awareness steps, role of ASHA workers, and proposed honorarium increase.
17LSI	17-06-2019	06-08-2019	Health issues among rural women, and schemes for improving their health.
17LSI	17-06-2019	06-08-2019	National average Infant Mortality Rate (IMR), Child Mortality Rate (CMR), and stillbirths, and steps to lower them.
17LSI	17-06-2019	06-08-2019	Non-payment of dues leading to refusal of child doses of HIV drugs, and government actions.
17LSI	17-06-2019	06-08-2019	Anemia as a significant public health issue, number of affected women, and government steps.
17LSII	18-11-2019	13-12-2019	Deaths of children due to pneumonia and diarrhea, awareness steps, and framework for Global Action Plan.
17LSII	18-11-2019	13-12-2019	Reduction in maternal mortality, access to quality maternity care, and prevalence of anemia among women.
17LSII	18-11-2019	13-12-2019	Increase in diseases like asthma, bronchitis, and pneumonia among children, vaccination details, and awareness steps.
17LSII	18-11-2019	13-12-2019	Prevalence of anemia among women, reasons for unsolved problem, and government initiatives.
17LSII	18-11-2019	13-12-2019	High child mortality due to pneumonia, shortage of specialist doctors, and corrective measures.

17LSII	18-11-2019	13-12-2019	High prevalence of anemia among women and children, schemes to address the issue, and steps to reduce anemia.
17LSII	18-11-2019	13-12-2019	National Nutrition Survey by UNICEF, findings on children's health, and government steps.
17LSIII	31-01-2020	23-03-2020	High prevalence of anemia among pregnant women, and steps taken.
17LSIV	14-09-2020	23-09-2020	Reasons for inefficiency in vaccinating children against measles, targets for eradication, and proposed laws for mandatory vaccination.
17LSIV	14-09-2020	23-09-2020	Vaccines administered to children during COVID-19 lockdown, missed immunizations, and policy to track and trace individuals.
17LSIV	14-09-2020	23-09-2020	Schemes for mitigating anemia and malnourishment among women and children, and shortcomings during COVID-19 pandemic.
17LSIX	18-07-2022	08-08-2022	Increase in anemia among children, alarming levels in certain states, and specific steps proposed.
17LSIX	18-07-2022	08-08-2022	Children missing regular vaccines due to COVID-19 duty, special campaigns for vaccination, and details of campaigns.
17LSIX	18-07-2022	08-08-2022	Steps to control population explosion, enforcement of child norms, and details of proposed measures.
17LSIX	18-07-2022	08-08-2022	COVID-19 infection among children, vaccination status, and details of registered and vaccinated children.
17LSIX	18-07-2022	08-08-2022	Impact of Intensified Diarrhoea Control Fortnight (IDFC) on child deaths, challenges faced, and related policies.
17LSIX	18-07-2022	08-08-2022	Infant mortality rate among Scheduled Tribes, reasons for high rates, and steps to reduce it.
17LSV	29-01-2021	25-03-2021	Children missing vaccination doses, remedial steps, and awareness campaigns.
17LSV	29-01-2021	25-03-2021	Delay in vaccination of children and pregnant women due to lockdown, and proposed studies and campaigns for missed vaccinations.
17LSV	29-01-2021	25-03-2021	Health issues related to bone fractures, steps for ensuring bone health, and measures to increase calcium levels in children.
17LSVI	19-07-2021	12-08-2021	Sharp decline in child immunization during COVID-19 pandemic, number of children vaccinated, and remedial steps.
17LSVI	19-07-2021	12-08-2021	Features of Mission Indradhanush, targets and achievements, and plan to include COVID-19 vaccine for children.
17LSVI	19-07-2021	12-08-2021	Warnings about the third wave of COVID-19, preparedness for health infrastructure, and vaccination for children.
17LSVI	19-07-2021	12-08-2021	Policy to check population growth, proposal for a two-child policy, and timeline for implementation.
17LSVI	19-07-2021	12-08-2021	COVID-19 cases among children, vaccines in trial stage, and expected availability.
17LSVI	19-07-2021	12-08-2021	Compulsory vaccines for children, disruption due to COVID-19, decline in vaccination rates, and alternate routes for vaccine delivery.
17LSVI	19-07-2021	12-08-2021	High child death rate, reasons, and steps taken to reduce it.
17LSVI	19-07-2021	12-08-2021	Free COVID-19 vaccination for all, vaccination for children, and steps to fully vaccinate the population.
17LSVII	29-11-2021	22-12-2021	High prevalence of stunting and wasting among children, steps taken to reduce it, and reasons for stunting without wasting.
17LSVII	29-11-2021	22-12-2021	Introduction of nasal vaccine for children, tests conducted, launch timeline, and steps to protect children from COVID-19.
17LSVII	29-11-2021	22-12-2021	COVID-19 vaccination status, free and paid vaccinations, need for booster doses, and vaccination for children.
17LSVII	29-11-2021	22-12-2021	Child vaccination coverage for various vaccines, trends observed, and reasons for low coverage.
17LSVII	29-11-2021	22-12-2021	Plan to increase COVID-19 vaccination rollout, research on vaccination for children, and details thereof.
17LSVII	29-11-2021	22-12-2021	Approval for vaccination of children below 18 years, and action taken.

17LSVII	29-11-2021	22-12-2021	Roadmap for COVID-19 vaccination for children aged 12-18, clinical trials, and vaccine availability.
17LSVII	29-11-2021	22-12-2021	Nationwide expansion of Pneumococcal Conjugate Vaccine under UIP, and impact on child mortality rate.
17LSVIII	31-01-2022	07-04-2022	Status of anemia among women and children, steps taken, and impact of malnutrition.
17LSVIII	31-01-2022	07-04-2022	Reduction in donations for treatment of children with rare diseases, and measures to ensure sufficient funds.
17LSVIII	31-01-2022	07-04-2022	Setting up of Newborn Care Units, role of ASHAs, and reduction in Infant Mortality Rate.
17LSVIII	31-01-2022	07-04-2022	WHO's concern over anemia in pregnant women, steps taken, and corrective measures.
17LSVIII	31-01-2022	07-04-2022	Number of children diagnosed with Spinal Muscular Atrophy (SMA), awareness measures, affordability of Risdiplam/Evrysdi, and care during the pandemic.
17LSVIII	31-01-2022	07-04-2022	Impact of Omicron variant on children, vaccination targets, guidelines issued, and vaccines in development for children.
17LSVIII	31-01-2022	07-04-2022	Deaths of tribal children due to malnutrition, steps to ensure availability of food and medical facilities, and corrective actions.
17LSVIII	31-01-2022	07-04-2022	Details of dedicated oncology departments in district hospitals, proposal for new oncology department in Idukki, and help for children with cancer.
17LSVIII	31-01-2022	07-04-2022	Newborn deaths due to sepsis caused by antimicrobial resistance, measures to manage resistance, and prevent deaths.
17LSVIII	31-01-2022	07-04-2022	Approval of Corbevax vaccine for children aged 12-14, safety and efficacy data, and peer review status.
17LSVIII	31-01-2022	07-04-2022	Facility-based newborn care units, role of ASHAs, and measures to reduce Infant Mortality Rate (IMR).
17LSX	07-12-2022	23-12-2022	Deaths of children after consuming contaminated cough syrup, steps to ensure quality of pharmaceutical products, and actions against offenders.
17LSX	07-12-2022	23-12-2022	Scheme for timely treatment of children with rare diseases, financial assistance for organ transplants, and upgradation of medical colleges.
17LSX	07-12-2022	23-12-2022	Implementation of Mission Indradhanush 4.0, benefits for children and women, and extent of vaccination conducted.
17LSX	07-12-2022	23-12-2022	Growing cases of anemia among children and women, initiatives to address adolescent anemia, and health clinics for adolescents.
17LSXI	31-01-2023	06-04-2023	Coverage under Mission Indradhanush, diseases for which immunization is provided, and steps for immunizing left-out children.
17LSXI	31-01-2023	06-04-2023	Vaccination programs for children in Tamil Nadu, funds allocated, and vacancies in health centers.
17LSXI	31-01-2023	06-04-2023	Salient features of National Health Mission, funds allocated and utilized, and decline in Maternal Mortality Ratio (MMR).
17LSXI	31-01-2023	06-04-2023	Vaccination coverage among disabled and tribal children, and drivers of vaccine hesitancy and uptake.
17LSXI	31-01-2023	06-04-2023	Steps to tackle rise in anemia among children in Jammu and Kashmir, identification of High Priority Districts, and interventions for reduction.
17LSXI	31-01-2023	06-04-2023	Special campaign for vaccination of children with incomplete cycles, and details of the campaign.
17LSXI	31-01-2023	06-04-2023	Newborn deaths due to sepsis caused by antimicrobial resistance, and steps to manage resistance.
17LSXI	31-01-2023	06-04-2023	Proposal to offer mental health services at PHCs, support for children with mental health issues, and study on rural mental health challenges.
17LSXI	31-01-2023	06-04-2023	Neonatal mortality rate, preventable causes of newborn deaths, and schemes to protect newborns.
17LSXI	31-01-2023	06-04-2023	Main features of Anaemia Mukt Bharat Abhiyan, funds allocated, and steps to address child wasting and stunting.
17LSXI	31-01-2023	06-04-2023	Decrease in maternal mortality rate, number of maternal deaths, and steps to achieve reduction targets.

17LSXI	31-01-2023	06-04-2023	Implementation of Pradhan Mantri Surakshit Matritva Abhiyan in Uttar Pradesh, guidelines issued, and number of beneficiaries.
17LSXI	31-01-2023	06-04-2023	Probe into deaths of children in Gambia and Uzbekistan due to cough syrup, actions taken, and communications received.
17LSXI	31-01-2023	06-04-2023	Improvement in access to menstrual hygiene products for rural women, and number of beneficiaries of antenatal and maternal health care schemes.
17LSXI	31-01-2023	06-04-2023	Health issues among rural women, schemes for improving health, and financial support for ST category women and children.
17LSXII	20-07-2023	11-08-2023	New schemes for the development of rural women and children, and funds allocated for Maharashtra.
17LSXII	20-07-2023	11-08-2023	Increase in Type One Diabetes, measures taken, and issues with insurance coverage for children with Type One Diabetes.
17LSXII	20-07-2023	11-08-2023	Allocation and utilization of funds under Navjaat Shishu Suraksha Karyakram, steps for early detection of diseases, and number of beneficiaries.
17LSXII	20-07-2023	11-08-2023	Details of Corona vaccines procured, distribution of doses, and vaccination coverage for children.
17LSXII	20-07-2023	11-08-2023	Root causes of childhood anemia, promotion of breastfeeding, and access to affordable nutritious food.
17LSXII	20-07-2023	11-08-2023	Status of COVID-19 vaccination drive for children, reasons for non-availability of vaccine slots, and doses administered.
17LSXII	20-07-2023	11-08-2023	Status of Surakshit Matritva Aashwasan (SUMAN) scheme and Janani Shishu Suraksha Karyakram (JSSK) in Uttar Pradesh, and financial support provided.
17LSXII	20-07-2023	11-08-2023	Steps to enforce one or two child norms/policy for population control, and details of the plan.
17LSXII	20-07-2023	11-08-2023	Number of maternal deaths, stillbirths, and newborn deaths, and budgetary expenditure under Pradhan Mantri Surakshit Matritva Abhiyan (PMSMA).
17LSXII	20-07-2023	11-08-2023	Health facilities in tribal districts of Madhya Pradesh, availability of doctors, and decline in Maternal Mortality Rate (MMR) and Infant Mortality Rate (IMR).
17LSXII	20-07-2023	11-08-2023	Increase in cancer cases among children, steps for awareness, prevention, and treatment.
17LSXIV	04-12-2023	22-12-2023	Features and objectives of Kilkari Scheme and Mission Utkarsh, and impact on maternal and child health.
17LSXIV	04-12-2023	22-12-2023	Children missing measles vaccine doses, steps for supplementary immunization, and catch-up vaccination age.
17LSXIV	04-12-2023	22-12-2023	Initiatives to improve maternal and childcare health services, budget allocated, and details for West Bengal.
17LSXIV	04-12-2023	22-12-2023	Pneumonia as a leading cause of death among children, details of cases, and action taken for prevention.
17LSXIV	04-12-2023	22-12-2023	Nutritional deprivation in Bihar, child and maternal mortality rates, and policies to improve health status.
17LSXIV	04-12-2023	22-12-2023	Changes in maternal and infant mortality rates, measures taken, and collaboration with international organizations.
17LSXIV	04-12-2023	22-12-2023	Study on rural mental health challenges, mental health services at PHCs, and support for children with mental health issues.
17LSXV	31-01-2024	10-02-2024	National Health Mission (NHM) launch date, objectives, achievements, and reduction in maternal and child mortality rates.
17LSXV	31-01-2024	10-02-2024	Plans to enhance accessibility and quality of healthcare for pregnant women and young children, and steps to improve maternal and child health outcomes.
17LSXV	31-01-2024	10-02-2024	Health issues among rural women, schemes for improving health, and financial support for SC/ST category women and children.
17LSXV	31-01-2024	10-02-2024	Statistics on Type-I Diabetes patients, welfare measures for children, and financial assistance for costly treatments.
17LSXV	31-01-2024	10-02-2024	Plan expenditure for nutrition of post-delivery women and children, and healthcare for poor old women.
Theme: Mental health			

Lok Sabha session	Session start date	Session end date	Summaries of questions asked
17LSVIII	31-01-2022	07-04-2022	Outreach measures for supporting mental health of housewives, and services availed under Manodarpan.
17LSVIII	31-01-2022	07-04-2022	High prevalence of mental illness, action plan to address it, and financial assistance for affected families.
17LSVIII	31-01-2022	07-04-2022	Increasing mental health patients, access to services under NMHP, and steps to improve mental health facilities.
17LSVIII	31-01-2022	07-04-2022	Increase in patients with mental stress and anxiety, steps to prevent depression, and studies conducted.
17LSXI	31-01-2023	06-04-2023	Rise in mental illness cases post-COVID-19, strategy to tackle the crisis, and shortage of mental health facilities.
17LSXI	31-01-2023	06-04-2023	Proposal to offer mental health services at PHCs, support for children with mental health issues, and study on rural mental health challenges.
17LSXII	20-07-2023	11-08-2023	Report on stress and anxiety among youth, action plan to protect affected individuals, and state-wise details.
17LSXII	20-07-2023	11-08-2023	Impact of lifestyle changes on mental health, number of affected individuals, and steps to protect future population.
17LSXII	20-07-2023	11-08-2023	Improvement in mental health services, accessibility and affordability, and increase in trained professionals.
17LSXII	20-07-2023	11-08-2023	Analysis of mental health patient-to-psychiatrist ratio, mandate for increasing mental health institutions, and steps taken.
17LSXII	20-07-2023	11-08-2023	Access to mental healthcare in rural areas, initiatives to address stigma, and high prevalence of depression among rural women.
17LSXII	20-07-2023	11-08-2023	Access to mental health treatment, steps to address the issue, and development of infrastructure for mental health services.
17LSXII	20-07-2023	11-08-2023	National Mental Health Programme in rural areas, plans for free counseling services, and impact of COVID-19 on mental health.
17LSXIV	04-12-2023	22-12-2023	Access to mental health facilities in rural areas, funds allocated, and infrastructure resistant to natural calamities.
17LSXIV	04-12-2023	22-12-2023	Proposal to set up medical colleges in each district, super specialty hospitals for mental diseases, and duration of medical courses.
17LSXIV	04-12-2023	22-12-2023	Impact of social connection on depression, schemes for medical illnesses, and corrective steps taken.
17LSXIV	04-12-2023	22-12-2023	Study on rural mental health challenges, mental health services at PHCs, and support for children with mental health issues.
17LSXV	31-01-2024	10-02-2024	Access to healthcare services for transgender community, support under National AIDS Control Programme-IV, and mental health policies.
17LSXV	31-01-2024	10-02-2024	Impact of climate disasters on mental health, steps to support vulnerable persons, and special mental health services in vulnerable states.
17LSXV	31-01-2024	10-02-2024	Mental health facilities and services in Meghalaya, impact of COVID-19 on mental health, and shortage of mental health experts in North Eastern Region.
17LSXV	31-01-2024	10-02-2024	Statistics on youth suffering from depression, anxiety, and stress, and action taken to protect affected individuals.
17LSXV	31-01-2024	10-02-2024	Mental health disorders in India, progress in implementing National Mental Health Policy 2014, and steps to increase availability of mental health professionals.
Theme: Non-communicable diseases			
**Lok Sabha session**	**Session start date**	**Session end date**	**Summaries of questions asked**
16LSXI	31-01-2017	12-04-2017	Government exploration of genomics for precision medicine in non-communicable diseases.
16LSXI	31-01-2017	12-04-2017	Approved anti-diabetes drugs in the Indian market.
16LSXI	31-01-2017	12-04-2017	Safe disposal of insulin syringes and pen needles, and government measures.

16LSXI	31-01-2017	12-04-2017	Rise in diabetes cases and government programs for prevention and treatment.
16LSXII	17-07-2017	11-08-2017	Diarrhea cases in the country, reasons, and government measures with allocated funds.
16LSXIII	15-12-2017	05-01-2018	Measures and schemes to control diabetes, risks of lung diseases, and government action plans.
16LSXIV	29-01-2018	06-04-2018	Cases and deaths related to ischemic diseases and government measures.
16LSXIV	29-01-2018	06-04-2018	Rise in vitiligo cases, treatment facilities, and clinical trials.
16LSXV	18-07-2018	10-08-2018	WHO's concern about air pollution and early onset of diabetes, and action plan for neonatal sepsis.
16LSXVI	11-12-2018	08-01-2019	Adverse drug reactions associated with new anti-diabetes medicines (SGLT-2 inhibitors) and government actions.
16LSXVI	11-12-2018	08-01-2019	Observance of World Diabetes Day, targets to control diabetes, and steps for prevention and treatment.
17LSI	17-06-2019	06-08-2019	Adverse drug reactions associated with new anti-diabetes medicines (SGLT-2 inhibitors), and government actions.
17LSI	17-06-2019	06-08-2019	Promotion of biologic medicines for non-communicable diseases, and separate fund allocation.
17LSII	18-11-2019	13-12-2019	Adverse drug reactions associated with new anti-diabetes medicines (SGLT-2 inhibitors), and government actions.
17LSIV	14-09-2020	23-09-2020	Adverse drug reactions associated with new anti-diabetes medicines (SGLT-2 inhibitors), and government actions.
17LSV	29-01-2021	25-03-2021	Observance of World Diabetes Day, increase in diabetes cases, and steps for prevention and treatment.
17LSVIII	31-01-2022	07-04-2022	Number of Type-1 diabetes patients, treatment measures, and financial assistance for insulin therapy.
17LSVIII	31-01-2022	07-04-2022	Upward trend in cancer, diabetes, and HIV cases, and remedial actions taken.
17LSX	07-12-2022	23-12-2022	Progress under National Programme for Prevention and Control of Cancer, Diabetes, Cardiovascular diseases and Stroke (NPPCDCS), and shortage of healthcare staff.
17LSX	07-12-2022	23-12-2022	India's high diabetes patient count, support for diabetic care, and funding for diabetes research.
17LSXI	31-01-2023	06-04-2023	Impact of COVID-19 on diabetic patients, steps to manage and prevent diabetes, and financial burden on poor families.
17LSXI	31-01-2023	06-04-2023	Rise in cardiovascular and lifestyle diseases, estimated number of sufferers, and measures taken to reduce incidence.
17LSXI	31-01-2023	06-04-2023	Number of deaths due to heart attack, causes and remedies, and steps to create awareness about silent heart attacks.
17LSXII	20-07-2023	11-08-2023	Report on the projected increase in diabetes cases, current status, and measures to control the disease.
17LSXII	20-07-2023	11-08-2023	Increase in Type One Diabetes, measures taken, and issues with insurance coverage for children with Type One Diabetes.
17LSXII	20-07-2023	11-08-2023	Survey on diabetes prevalence, steps to provide affordable testing kits, and awareness programs.
17LSXII	20-07-2023	11-08-2023	Discrepancies in cataract surgery coverage, complaints about excessive fees, and welfare measures for Type-1 diabetes patients.
17LSXII	20-07-2023	11-08-2023	Increase in cardiac deaths post-COVID-19, reasons, and schemes to prevent the disease.
17LSXII	20-07-2023	11-08-2023	Diabetes statistics in India, awareness of diabetic status, and plans to make India diabetes-free.
17LSXII	20-07-2023	11-08-2023	High prevalence of non-communicable diseases in Kerala, and consideration of sending a team of medical experts.
17LSXIV	04-12-2023	22-12-2023	Increase in diabetes cases among individuals under forty, reasons identified, and measures to create awareness.
17LSXV	31-01-2024	10-02-2024	Statistics on Type-I Diabetes patients, welfare measures for children, and financial assistance for costly treatments.

Theme: Vaccines and immunization			
**Lok Sabha session**	**Session start date**	**Session end date**	**Summaries of questions asked**
16LSXI	31-01-2017	12-04-2017	Concerns about risks from the measles-rubella vaccine for pregnant women and unborn babies, and government measures to ensure their safety.
16LSXI	31-01-2017	12-04-2017	Differences in immunization programs between government and private hospitals, amendments to the Universal Immunization Programme, and efforts to cover all children.
16LSXI	31-01-2017	12-04-2017	Number of rabies deaths, availability of anti-rabies vaccine, and reasons for any shortages.
16LSXI	31-01-2017	12-04-2017	Introduction of Pneumococcal Conjugate Vaccine in five states and plans for expansion.
16LSXI	31-01-2017	12-04-2017	Phase-II and Phase-III of Mission Indradhanush, funds allocation, and vaccination achievements.
16LSXI	31-01-2017	12-04-2017	Government response to paediatricians recommending vaccines to favor companies.
16LSXI	31-01-2017	12-04-2017	WHO rating of India's vaccine regulatory structure and related details.
16LSXI	31-01-2017	12-04-2017	Adequate IPV vaccine supplies and reported shortages.
16LSXI	31-01-2017	12-04-2017	Government preparedness for Ebola and development of drugs and vaccines.
16LSXII	17-07-2017	11-08-2017	Shortage of vaccines, proposals to restart production in specific labs, and safety norms.
16LSXII	17-07-2017	11-08-2017	Shortage of Inactivated Polio Vaccine (IPV) in government and private hospitals, reasons, and corrective measures.
16LSXIII	15-12-2017	05-01-2018	Strengthening activities of Central Research Institute, Kasauli, and vaccine production details.
16LSXIII	15-12-2017	05-01-2018	Availability and promotion of cancer vaccines.
16LSXIII	15-12-2017	05-01-2018	Introduction of HPV vaccine under the universal immunization program to prevent cervical cancer.
16LSXIII	15-12-2017	05-01-2018	Shortage of Inactivated Polio Vaccine (IPV) and steps to address it.
16LSXIII	15-12-2017	05-01-2018	Availability of essential vaccines in government hospitals and steps to include them.
16LSXIII	15-12-2017	05-01-2018	Review of disease incidence and vaccine availability, and regular vaccination plans.
16LSXIII	15-12-2017	05-01-2018	Approval for clinical trials of dengue vaccine and expected availability.
16LSXIII	15-12-2017	05-01-2018	Mission Indradhanush vaccination programs and protocols for vaccine storage and handling.
16LSXIII	15-12-2017	05-01-2018	Efficacy of Zika virus vaccine in animal trials and protection against different strains.
16LSXIV	29-01-2018	06-04-2018	Deaths due to Pentavalent Vaccine (PV) and government studies.
16LSXIV	29-01-2018	06-04-2018	Rollout of Pneumococcal and Rotavirus vaccinations in selected districts.
16LSXIV	29-01-2018	06-04-2018	Production and shortage of Yellow Fever vaccine and corrective steps.
16LSXIV	29-01-2018	06-04-2018	Study on rise in adverse events following vaccination.
16LSXIV	29-01-2018	06-04-2018	Shortage of Inactivated Polio Vaccine (IPV) and impact on children's immunity.
16LSXIV	29-01-2018	06-04-2018	Hepatitis cases and deaths, government targets, and vaccination coverage.
16LSXIV	29-01-2018	06-04-2018	Post-immunization complications and deaths, and government inquiries.

16LSXIV	29-01-2018	06-04-2018	Decision against introducing cervical cancer vaccine in public health programs.
16LSXIV	29-01-2018	06-04-2018	Launch and impact of Rotavirus vaccine to prevent Rotavirus Diarrhea.
16LSXV	18-07-2018	10-08-2018	Government plans to eliminate measles by 2020, deaths reported, and immunization programs.
16LSXVI	11-12-2018	08-01-2019	Lack of vaccine storage facilities in rural areas, financial and technical support, and use of solar-powered refrigerators.
16LSXVI	11-12-2018	08-01-2019	Inquiry into type 2 polio virus contamination in immunization vials and additional immunization.
16LSXVII	31-01-2019	13-02-2019	Rise in polio vaccine prices, funds sought from international donors, and impact on vaccine availability.
17LSI	17-06-2019	06-08-2019	Acute shortage of rabies injections, reported deaths, and steps to ensure supply.
17LSI	17-06-2019	06-08-2019	Awareness programs for parents about vaccination, addressing vaccine hesitancy, and role of ASHA workers.
17LSI	17-06-2019	06-08-2019	Type-II polio virus infection found in immunization injections, probe details, and additional immunization plans.
17LSI	17-06-2019	06-08-2019	Government-controlled vaccine production units, vaccine shortages, and steps to ensure adequate availability.
17LSI	17-06-2019	06-08-2019	Implementation of Universal Immunization Programme (UIP) in tribal and remote areas, and introduction of pneumonia vaccine.
17LSII	18-11-2019	13-12-2019	Availability of vaccines for Pneumococcal Conjugate, Rotavirus, and Japanese Encephalitis, and steps to address the issue.
17LSII	18-11-2019	13-12-2019	Financial and technical support for vaccine storage in rural areas, and use of solar-powered refrigerators.
17LSII	18-11-2019	13-12-2019	Funds disbursed for rabies vaccination, domestic demand and export control, and inclusion in universal immunization program.
17LSII	18-11-2019	13-12-2019	Steps to eliminate Lymphatic Filariasis, vaccination programs, and awareness measures.
17LSII	18-11-2019	13-12-2019	Cases and deaths due to rabies, shortage of Anti-Rabies Vaccine, and inclusion in universal immunization program.
17LSIII	31-01-2020	23-03-2020	Financial and technical support for vaccine storage, and use of solar-powered refrigerators.
17LSIII	31-01-2020	23-03-2020	Impact of Coronavirus, research on vaccine, and public health policy.
17LSIV	14-09-2020	23-09-2020	Claims of vaccine development for coronavirus, examination of claims, and steps to produce a vaccine.
17LSIV	14-09-2020	23-09-2020	Vaccines being developed for coronavirus, progress of trials, and vaccination schedule for India.
17LSIV	14-09-2020	23-09-2020	Vaccines administered to children during COVID-19 lockdown, missed immunizations, and policy to track and trace individuals.
17LSIV	14-09-2020	23-09-2020	Standard procedure for vaccine research and development, deviations for coronavirus vaccine, and human trials conducted.
17LSIV	14-09-2020	23-09-2020	Development of coronavirus vaccine, other measures to control the pandemic, and improvement of district-level hospitals.
17LSIV	14-09-2020	23-09-2020	Vaccine strategy for COVID-19, vaccine candidates being developed, and international efforts for equitable access.
17LSIV	14-09-2020	23-09-2020	Foreign nations developing coronavirus vaccines, human trials, and talks with Russia and other countries for vaccine procurement.
17LSIV	14-09-2020	23-09-2020	Orders to ICMR for developing a vaccine, status of research programs, and financial assistance provided.
17LSIV	14-09-2020	23-09-2020	COVID-19 cases, deaths, and recoveries, research on vaccine development, and collaboration with international organizations.
17LSIV	14-09-2020	23-09-2020	Uncertainty of COVID-19 vaccine due to virus mutation, present status of research, and timeline for vaccine availability.
17LSIX	18-07-2022	08-08-2022	Susceptibility of vaccines to Omicron sub-variants, risks of infectious diseases, and details of booster doses.

17LSIX	18-07-2022	08-08-2022	Free booster doses of COVID-19 vaccine, increase in uptake, and arrangements for sufficient vaccine doses.
17LSIX	18-07-2022	08-08-2022	Coverage of Mission Indradhanush, diseases covered, and emphasis on immunization in tribal districts.
17LSIX	18-07-2022	08-08-2022	Children missing regular vaccines due to COVID-19 duty, special campaigns for vaccination, and details of campaigns.
17LSIX	18-07-2022	08-08-2022	Reduction of booster dose interval for COVID-19, performance of Andhra Pradesh, and efforts to expedite coverage.
17LSIX	18-07-2022	08-08-2022	Efficacy of COVID-19 vaccines, coverage under Har Ghar Dastak campaign, and details of booster doses administered.
17LSIX	18-07-2022	08-08-2022	Action plan for booster doses, effectiveness, and steps to create awareness.
17LSIX	18-07-2022	08-08-2022	COVID-19 infections after vaccination, state-wise and vaccine-wise details.
17LSIX	18-07-2022	08-08-2022	COVID-19 vaccination progress, plans for booster doses, and details of vaccination for people below 18 years.
17LSV	29-01-2021	25-03-2021	COVID-19 vaccination for people above 60 years and those with comorbidities, and details of vaccination centers.
17LSV	29-01-2021	25-03-2021	Regulatory processes for vaccine approval, details of Covishield and Covaxin approvals, and recommendations of the Subject Experts Committee.
17LSV	29-01-2021	25-03-2021	Approval of foreign-origin COVID vaccines, regulatory requirements, and consultations with vaccine manufacturers.
17LSV	29-01-2021	25-03-2021	Methodology for vaccine distribution, and utilization of vaccine doses.
17LSV	29-01-2021	25-03-2021	Lower vaccination rates among health workers, reasons, and details of arrangements.
17LSV	29-01-2021	25-03-2021	COVID-19 vaccination for the general public, targeted population, and state-wise details.
17LSV	29-01-2021	25-03-2021	Features of COVID-19 vaccine in Assam, districts covered, and steps for remote areas.
17LSV	29-01-2021	25-03-2021	COVID vaccines in the pipeline, trial phases, and details of intranasal vaccine development.
17LSV	29-01-2021	25-03-2021	Mutations of COVID-19 virus, efficacy of vaccines, and steps to check the spread of mutant strains.
17LSV	29-01-2021	25-03-2021	Details of people vaccinated, doses bought and exported, and adverse events following immunization.
17LSV	29-01-2021	25-03-2021	Side effects of COVID-19 vaccines, reasons for lack of guarantee, and steps to compensate affected individuals.
17LSV	29-01-2021	25-03-2021	Issues with vaccination portal in Jabalpur, and remedial measures taken.
17LSV	29-01-2021	25-03-2021	Beneficiaries of COVID vaccination, registration on Co-WIN platform, and documents required.
17LSV	29-01-2021	25-03-2021	Containment and control of COVID-19 cases, vaccination status, and steps to speed up vaccination.
17LSV	29-01-2021	25-03-2021	Daily vaccination rate for COVID-19, plans to increase the rate, and details of people inoculated with the second dose.
17LSV	29-01-2021	25-03-2021	Rural healthcare workers as frontline workers, and provision of free COVID-19 vaccine.
17LSV	29-01-2021	25-03-2021	Cervical cancer cases, proposal to include HPV vaccine, and awareness plans.
17LSV	29-01-2021	25-03-2021	Rollout of the second dose of COVID-19 vaccine, and details of beneficiaries.
17LSV	29-01-2021	25-03-2021	Development of nasal vaccine for COVID-19, and details of vaccination mechanism.
17LSV	29-01-2021	25-03-2021	Approval of vaccines that have undergone phase 3 trials outside India, and use of CSR funds for vaccination.
17LSV	29-01-2021	25-03-2021	Frozen and damaged vaccine doses, details of vaccinated health workers, and funds utilized.

17LSV	29-01-2021	25-03-2021	Expected number of people to be vaccinated in the first phase, and details of vaccine orders.
17LSV	29-01-2021	25-03-2021	Number of doses required for COVID-19 vaccination, and awareness drive to instill confidence.
17LSV	29-01-2021	25-03-2021	Manufacturers of COVID vaccines, and details of phase 3 trial results.
17LSV	29-01-2021	25-03-2021	COVID-19 vaccine operational guidelines, identification of vaccination categories, and database on people with comorbidities
17LSV	29-01-2021	25-03-2021	Number of people administered Covishield/Covaxin, clearance for Pfizer-Biotech vaccine, and indemnity requests.
17LSV	29-01-2021	25-03-2021	Permission for sale of Covishield vaccine in the open market, affordability for vulnerable populations, and inclusion in the list of essential medicines.
17LSV	29-01-2021	25-03-2021	Higher cost of Oxford-AstraZeneca vaccine for India compared to the European Union, reasons for the price difference, and government negotiations for a lower price.
17LSV	29-01-2021	25-03-2021	Agreements with other countries for the supply of COVID-19 vaccines, details of agreements, and impact on domestic vaccine availability.
17LSV	29-01-2021	25-03-2021	Adverse effects of COVID-19 vaccines, inputs from experts, consent issues in vaccine trials, and next phase of vaccination.
17LSV	29-01-2021	25-03-2021	Tying up with multiple vaccine manufacturers, free vaccination for the entire population, and funds disbursed for protective gear.
17LSV	29-01-2021	25-03-2021	People not coming forward for COVID-19 vaccination, adverse reactions, and urgency of vaccination despite declining cases.
17LSV	29-01-2021	25-03-2021	Approval of Bharat Biotech's vaccine without phase 3 trial data, reasons for approval, and review of safety and efficacy data.
17LSV	29-01-2021	25-03-2021	Parameters for approving Covishield and Covaxin, publication of test results, and actions taken.
17LSV	29-01-2021	25-03-2021	Criteria for selecting and procuring COVID-19 vaccines, logistical hurdles, and steps to increase vaccine production.
17LSV	29-01-2021	25-03-2021	Identification of people with comorbidities for COVID-19 vaccination, and steps to tackle vaccine efficacy.
17LSV	29-01-2021	25-03-2021	Deviations in vaccine research and development, outcomes of human trials, and timeline for vaccine availability.
17LSV	29-01-2021	25-03-2021	Regulatory approval for Covishield and Covaxin, phase 3 trials, and issues with informed consent in vaccine trials.
17LSV	29-01-2021	25-03-2021	Consultation with experts for vaccine usage, distribution details, and number of vaccines distributed to states.
17LSV	29-01-2021	25-03-2021	Pan India rollout of COVID-19 vaccination drive, criteria for vaccine distribution, and infrastructure for vaccine movement.
17LSV	29-01-2021	25-03-2021	Doses distributed for COVID-19 vaccination, states where vaccines have not been supplied, and measures for successful vaccination.
17LSV	29-01-2021	25-03-2021	Phased availability of COVID-19 vaccines, schedule and priority for immunization, and sufficiency of cold storage facilities.
17LSV	29-01-2021	25-03-2021	Availability of COVID-19 vaccines for the entire population, pricing details, and number of vaccinations carried out.
17LSVI	19-07-2021	12-08-2021	Data on prisoners receiving COVID-19 vaccination, strategy for complete inoculation, and advisory notifications to states.
17LSVI	19-07-2021	12-08-2021	Financial resources required for vaccinating youth and middle-aged population, issues with universal vaccination, and corrective steps.
17LSVI	19-07-2021	12-08-2021	Proposal to manufacture a nasal vaccine for COVID-19, tests done, and vaccination mechanism.
17LSVI	19-07-2021	12-08-2021	Sharp decline in child immunization during COVID-19 pandemic, number of children vaccinated, and remedial steps.
17LSVI	19-07-2021	12-08-2021	Policy decisions for access to COVID-19 testing, treatment, and vaccines for vulnerable groups, and outcomes of initiatives.
17LSVI	19-07-2021	12-08-2021	Vaccine manufacturing capacity, COVID-19 vaccines produced, and steps to increase production.

17LSVI	19-07-2021	12-08-2021	Complaints about private hospitals selling free COVID-19 vaccines, and actions taken.
17LSVI	19-07-2021	12-08-2021	Types of COVID-19 vaccines available, number of people vaccinated, and measures to make Covaxin available.
17LSVI	19-07-2021	12-08-2021	Shortage of COVID-19 vaccines, idle vaccine manufacturing plant in Chengalpattu, and plans for utilization.
17LSVI	19-07-2021	12-08-2021	Expenditure on COVID-19 vaccination, testing capability, and pace of vaccination in tribal areas.
17LSVI	19-07-2021	12-08-2021	Proportion of individuals vaccinated without CoWIN registration, measures for those without digital access or photo ID.
17LSVI	19-07-2021	12-08-2021	Myths about COVID-19 vaccine causing infertility, awareness measures, and steps to ensure vaccine safety.
17LSVI	19-07-2021	12-08-2021	Target for full COVID-19 vaccination by December 2021, daily doses required, and timeline for 100% vaccination.
17LSVI	19-07-2021	12-08-2021	Administration of COVID-19 vaccines through private hospitals, price details, and booking of vaccination sites.
17LSVI	19-07-2021	12-08-2021	Infectiousness of new coronavirus strains, reasons for surge in cases, and efficacy of vaccines against new strains.
17LSVI	19-07-2021	12-08-2021	Development of COVID-19 vaccines by government organizations, contribution percentages, and profit or royalty details.
17LSVI	19-07-2021	12-08-2021	Administration of Covaxin before phase 3 data publication, issues with trial rules, and use in clinical trial mode.
17LSVI	19-07-2021	12-08-2021	Policy to control modified COVID-19 variants, and steps to enhance vaccination rate.
17LSVI	19-07-2021	12-08-2021	Features of Mission Indradhanush, targets and achievements, and plan to include COVID-19 vaccine for children.
17LSVI	19-07-2021	12-08-2021	Confusion about effectiveness of Covaxin and Covishield, publication of data, and efforts to clear misconceptions.
17LSVI	19-07-2021	12-08-2021	Data on Vaccine Preventable Diseases (VPDs) covered under Universal Immunization Programme, and immunization coverage.
17LSVI	19-07-2021	12-08-2021	Targets and timeline for COVID-19 vaccination, stock of vaccines, and integration with other immunization programs.
17LSVI	19-07-2021	12-08-2021	COVID-19 deaths post-vaccination, WHO inclusion of Covaxin, and travel issues due to non-approval.
17LSVI	19-07-2021	12-08-2021	States still affected by the second wave of COVID-19, steps taken, and vaccination status.
17LSVI	19-07-2021	12-08-2021	COVID-19 vaccines supplied to Tamil Nadu, requests for additional vaccines, and actions taken.
17LSVI	19-07-2021	12-08-2021	Side effects of COVID-19 vaccines, and reasons for lack of guarantee.
17LSVI	19-07-2021	12-08-2021	Features of COVID-19 vaccine in Odisha, number of people vaccinated, and steps for remote areas.
17LSVI	19-07-2021	12-08-2021	Liberalized and Accelerated Phase-3 Strategy of COVID-19 Vaccination, vaccine procurement by states, and demand for medical supplies.
17LSVI	19-07-2021	12-08-2021	Free COVID-19 vaccination for the entire population, target for vaccine doses, and pace of vaccination.
17LSVI	19-07-2021	12-08-2021	Global tenders for vaccine procurement by states, guarantee of supply, and assurance from indigenous manufacturers.
17LSVI	19-07-2021	12-08-2021	Vaccination of the general public, difficulties in vaccine procurement, and criteria for vaccine allocation.
17LSVI	19-07-2021	12-08-2021	COVID-19 vaccines approved for use, approval process, and percentage of rural population vaccinated.
17LSVI	19-07-2021	12-08-2021	Study on deaths related to Adverse Events Following Immunization (AEFI), need for revamping health infrastructure, and plan for the third wave.
17LSVI	19-07-2021	12-08-2021	COVID-19 cases among children, vaccines in trial stage, and expected availability.
17LSVI	19-07-2021	12-08-2021	Review of side effects linked to COVID-19 vaccines, number of deaths confirmed, and expert group for assessments.

17LSVI	19-07-2021	12-08-2021	Sufficiency of cold storage facilities for COVID-19 vaccines, and steps to expand storage capacity.
17LSVI	19-07-2021	12-08-2021	Low vaccination rate despite being a leading vaccine manufacturer, reasons, and steps to increase vaccination.
17LSVI	19-07-2021	12-08-2021	Changes in COVID-19 vaccine policy, criteria for distribution, and export of vaccines.
17LSVI	19-07-2021	12-08-2021	COVID-19 vaccine orders, funds allocated, and roadmap for vaccination.
17LSVI	19-07-2021	12-08-2021	Compulsory vaccines for children, disruption due to COVID-19, decline in vaccination rates, and alternate routes for vaccine delivery.
17LSVI	19-07-2021	12-08-2021	Steps to increase vaccine supply to states, analysis of supply chain issues, and timeline for universal vaccination.
17LSVI	19-07-2021	12-08-2021	Funds allocated for free COVID-19 vaccination, public and private manufacturers, and cost of vaccines.
17LSVI	19-07-2021	12-08-2021	Discussions with pharma companies for indemnity against vaccine side effects, demands from companies, and stance on domestic manufacturers.
17LSVI	19-07-2021	12-08-2021	Steps to minimize congenital disabilities, reasons for high incidence in rural areas, and extension of Rubella vaccination.
17LSVI	19-07-2021	12-08-2021	Assistance from foreign countries during the second wave, details of items received, and agreements for vaccine supply.
17LSVI	19-07-2021	12-08-2021	Distribution of COVID-19 vaccines to States/UTs, details for Telangana, and vaccination statistics.
17LSVI	19-07-2021	12-08-2021	COVID-19 vaccination statistics, vaccine hesitancy, and funds allocated for tackling COVID-19 in Assam and North Eastern States.
17LSVI	19-07-2021	12-08-2021	Vaccine hesitancy, COVID-19 vaccination coverage, and steps to accelerate vaccination.
17LSVI	19-07-2021	12-08-2021	Plan to complete COVID-19 vaccination of all adults by end of 2021, vaccine availability, and funds spent on vaccination.
17LSVI	19-07-2021	12-08-2021	Importance of full COVID-19 vaccination, inclusion of refugees and asylum seekers, and special vaccination sessions for undocumented communities.
17LSVII	29-11-2021	22-12-2021	Export of COVID-19 vaccines, production ramp-up, and impact on domestic supply.
17LSVII	29-11-2021	22-12-2021	Data on vaccines procured by private hospitals, vaccine wastage, and funds spent on procurement.
17LSVII	29-11-2021	22-12-2021	Financial and technical assistance for vaccine storage in rural areas, and promotion of solar-powered refrigerators.
17LSVII	29-11-2021	22-12-2021	Worst and best performing districts for second COVID-19 dose, reasons for reluctance, and steps to encourage vaccination.
17LSVII	29-11-2021	22-12-2021	Launch of new COVID-19 vaccination phase, vaccine wastage, and remedial measures.
17LSVII	29-11-2021	22-12-2021	Number of people fully vaccinated against COVID-19, provision of booster doses, and steps to reduce gap between Covishield doses.
17LSVII	29-11-2021	22-12-2021	Availability of COVID-19 vaccines, vaccination delays in Kerala, and steps to ensure timely second doses.
17LSVII	29-11-2021	22-12-2021	Delay in COVID-19 inoculation drive due to lack of advance agreements with foreign vaccine makers, and rollout of oral anti-COVID-19 drugs.
17LSVII	29-11-2021	22-12-2021	COVID-19 vaccination statistics, vaccines used, export permissions, and vaccine expiry issues.
17LSVII	29-11-2021	22-12-2021	COVID-19 vaccination status in Himachal Pradesh, vaccines provided, and timeline for second doses.
17LSVII	29-11-2021	22-12-2021	Benefits of WHO approval for Covaxin, recognition by countries, and impact on travel restrictions.
17LSVII	29-11-2021	22-12-2021	Identification of fake COVID-19 vaccines, number of patients affected, countermeasures taken, guidelines issued, and doses seized.
17LSVII	29-11-2021	22-12-2021	Free COVID-19 vaccines administered, number of beneficiaries, and state-wise details of first and second doses.

17LSVII	29-11-2021	22-12-2021	Shelf life of COVID-19 vaccines, redistribution of unused vaccines, booster dose preparedness, and steps to ensure vaccine supply.
17LSVII	29-11-2021	22-12-2021	Measures to expedite COVID-19 vaccination, policy for booster doses, studies on necessity and effectiveness, and steps for administering booster doses.
17LSVII	29-11-2021	22-12-2021	Features of COVID-19 vaccine in Odisha, districts covered, and steps for remote areas.
17LSVII	29-11-2021	22-12-2021	Cervical cancer cases and deaths, impact of HPV vaccine, inclusion in Universal Immunization Program, and compulsory smear tests for school girls.
17LSVII	29-11-2021	22-12-2021	Introduction of nasal vaccine for children, tests conducted, launch timeline, and steps to protect children from COVID-19.
17LSVII	29-11-2021	22-12-2021	Achievement of COVID-19 vaccination targets, cumulative coverage, surge in cases post-festival season, and preventive steps taken.
17LSVII	29-11-2021	22-12-2021	COVID-19 vaccine doses provided to Maharashtra, public response, and steps to dispel doubts about vaccination.
17LSVII	29-11-2021	22-12-2021	Dropout of second COVID-19 dose, identification and outreach plan, and health repercussions of missing second dose.
17LSVII	29-11-2021	22-12-2021	Data available with DGCI for Covaxin's EUA, and data sought by WHO for approval.
17LSVII	29-11-2021	22-12-2021	Vaccination of people with disabilities, priority notification, and details thereof.
17LSVII	29-11-2021	22-12-2021	Free COVID-19 vaccines administered, rural-urban and gender-wise breakup, and funds spent.
17LSVII	29-11-2021	22-12-2021	Production capacity of Covishield and Covaxin, approval of other manufacturers, and import permissions.
17LSVII	29-11-2021	22-12-2021	Child vaccination coverage for various vaccines, trends observed, and reasons for low coverage.
17LSVII	29-11-2021	22-12-2021	G-7 countries' vaccine donation, sufficiency for universal need, and steps to enhance production.
17LSVII	29-11-2021	22-12-2021	Roadmap for COVID-19 vaccination for children aged 12-18, clinical trials, and vaccine availability.
17LSVII	29-11-2021	22-12-2021	Nationwide expansion of Pneumococcal Conjugate Vaccine under UIP, and impact on child mortality rate.
17LSVIII	31-01-2022	07-04-2022	Expenditure on COVID-19 vaccination, rise in dengue cases, and plans for dengue treatment.
17LSVIII	31-01-2022	07-04-2022	Sufficiency of cold storage facilities for COVID-19 vaccines, and steps taken for additional storage.
17LSVIII	31-01-2022	07-04-2022	Reduction in public health budget, funding for booster doses, and deaths due to adverse effects of COVID-19 immunization.
17LSVIII	31-01-2022	07-04-2022	Monitoring adverse impact of COVID-19 vaccines on lactating mothers and infants, and recorded effects.
17LSVIII	31-01-2022	07-04-2022	Allocation and utilization of funds for COVID-19 vaccination, and need for additional funds.
17LSVIII	31-01-2022	07-04-2022	Unvaccinated population, utilization of vaccines with extended validity, and steps to forward stored vaccines.
17LSVIII	31-01-2022	07-04-2022	Cases of vector-borne diseases, steps taken, funds allocated, and development of malaria vaccine.
17LSVIII	31-01-2022	07-04-2022	Funds released for COVID-19 management under NHM, requests for vaccine allocation, and financial assistance during lockdown.
17LSVIII	31-01-2022	07-04-2022	Proposals to permit the sale of COVID-19 vaccines in medical shops, and steps taken by the Government.
17LSVIII	31-01-2022	07-04-2022	Preventing illnesses, hospitalizations, and deaths by increasing vaccine coverage for pneumonia and related complications.
17LSVIII	31-01-2022	07-04-2022	Genome sequencing for Omicron variant detection, official data representation, booster doses, and steps taken.
17LSVIII	31-01-2022	07-04-2022	Measures to provide vaccines under Atmanirbhar Bharat Yojana in Maharashtra, vaccination percentage, and timeline for complete vaccination.

17LSVIII	31-01-2022	07-04-2022	Impact of Omicron variant on children, vaccination targets, guidelines issued, and vaccines in development for children.
17LSVIII	31-01-2022	07-04-2022	Guidelines for deeming an individual as fully vaccinated in view of booster vaccinations.
17LSVIII	31-01-2022	07-04-2022	Outreach strategy to promote awareness about COVID-19 vaccination, and expenditure incurred for media outreach programs.
17LSVIII	31-01-2022	07-04-2022	Progress of vaccination of prisoners, number of prisoners contracting COVID-19, and steps to control the spread in jails.
17LSVIII	31-01-2022	07-04-2022	Study on COVID-19 deaths among vaccinated and unvaccinated persons, number of COVID-19 and Omicron patients, and lockdown proposals.
17LSVIII	31-01-2022	07-04-2022	Vaccination drive reaching 160 crore doses, number of doses administered by age group, and target for 100% vaccination.
17LSVIII	31-01-2022	07-04-2022	Administration of Covaxin in the 15-18 age group, approval status, and steps to get approval from other countries.
17LSVIII	31-01-2022	07-04-2022	Pathogenicity of Omicron variant, genomic variations monitoring by INSACOG, and details of Omicron cases and booster shots.
17LSVIII	31-01-2022	07-04-2022	Number of persons infected by Omicron variant, effectiveness of indigenous vaccines, and measures to prevent the spread.
17LSVIII	31-01-2022	07-04-2022	G-7 countries' announcement for providing vaccine doses to developing countries, number of doses received, and action taken.
17LSVIII	31-01-2022	07-04-2022	Financial and technical support for vaccine storage in rural areas, and promotion of solar-powered vaccine refrigerators.
17LSVIII	31-01-2022	07-04-2022	Approval of Corbevax vaccine for children aged 12-14, safety and efficacy data, and peer review status.
17LSVIII	31-01-2022	07-04-2022	Immunization vaccines administered by pediatricians in private clinics, high fees charged, and corrective measures.
17LSVIII	31-01-2022	07-04-2022	Coverage under Har Ghar Dastak door-to-door vaccination campaign, reasons for delay in achieving 100% vaccination, and revised timeline.
17LSVIII	31-01-2022	07-04-2022	Cases of fraud in COVID-19 vaccination and issuance of certificates, investigation of claims, and measures to prevent data tampering.
17LSVIII	31-01-2022	07-04-2022	Study on extending vaccination center hours, proposal for round-the-clock vaccination, and details of the plan.
17LSVIII	31-01-2022	07-04-2022	Decrease in the number of people taking the first dose of COVID-19 vaccination, target for full inoculation, and steps to promote vaccination.
17LSX	07-12-2022	23-12-2022	Proposal to manufacture cervical cancer vaccine, and steps taken in this regard.
17LSX	07-12-2022	23-12-2022	Number of fully vaccinated people, unused vaccines in government stock, and vaccines past their expiry date.
17LSX	07-12-2022	23-12-2022	India's achievement of administering over two billion COVID vaccine doses, challenges faced, and vaccine wastage.
17LSX	07-12-2022	23-12-2022	Vaccines procured from Zydus Cadila, cost per vaccine, and future vaccination plans.
17LSX	07-12-2022	23-12-2022	Diseases and side effects caused by dog bites, deaths due to rabies, and quality testing of vaccines.
17LSXI	31-01-2023	06-04-2023	Coverage under Mission Indradhanush, diseases for which immunization is provided, and steps for immunizing left-out children.
17LSXI	31-01-2023	06-04-2023	Vaccination coverage among disabled and tribal children, and drivers of vaccine hesitancy and uptake.
17LSXI	31-01-2023	06-04-2023	Development and launch of new cervical cancer vaccine, approval status, and production details.
17LSXI	31-01-2023	06-04-2023	Need for robust immunization programs in tribal areas of Gujarat, and plans to ease access to immunization facilities.
17LSXI	31-01-2023	06-04-2023	Introduction of fractional 3rd dose for Inactivated Polio Vaccine (IPV), reasons for additional dose, and communication to states.
17LSXI	31-01-2023	06-04-2023	Mechanism to report COVID-19 vaccine-related Adverse Events Following Immunization (AEFI), and steps to curb under-reporting.

17LSXI	31-01-2023	06-04-2023	Impact of COVID-19 on Universal Immunisation Programme (UIP), measures to ensure immunization, and vaccination coverage.
17LSXI	31-01-2023	06-04-2023	Incidences of instant death due to heart attack post-COVID-19, possible link to vaccines, and studies conducted.
17LSXI	31-01-2023	06-04-2023	Cervical cancer cases detected, penetration of HPV vaccine, and efforts to vaccinate women.
17LSXI	31-01-2023	06-04-2023	COVID-19 statistics, proposal for booster doses, and guidelines for passengers from certain countries.
17LSXI	31-01-2023	06-04-2023	Free COVID vaccination campaign, details of the campaign, and current status of execution.
17LSXI	31-01-2023	06-04-2023	Survey on vaccination coverage under Indradhanush Scheme, impact of COVID-19 on other immunization programs, and funds allocated.
17LSXI	31-01-2023	06-04-2023	Data on women suffering from cervical cancer, awareness programs, and rollout of cervical cancer vaccines.
17LSXI	31-01-2023	06-04-2023	Availability of Phase 2 trial results of COVAXIN, approval for Phase 3 trials, and vaccine efficacy data.
17LSXI	31-01-2023	06-04-2023	Scientific study on effectiveness of COVID vaccines and booster doses, and measures to strengthen health systems.
17LSXI	31-01-2023	06-04-2023	Purchase of COVID-19 booster dose vaccines, quantity purchased, and amount spent.
17LSXI	31-01-2023	06-04-2023	Steps to introduce vaccines for Chikungunya and Dengue, and details of initiatives.
17LSXII	20-07-2023	11-08-2023	Measles cases and deaths, vaccination coverage, and campaigns conducted.
17LSXII	20-07-2023	11-08-2023	Data on first, second, and booster doses of COVID-19 vaccine not administered, state-wise details.
17LSXII	20-07-2023	11-08-2023	States with less than 60% full immunization coverage, special measures to increase coverage, and steps for 100% immunization.
17LSXII	20-07-2023	11-08-2023	Details of Corona vaccines procured, distribution of doses, and vaccination coverage for children.
17LSXII	20-07-2023	11-08-2023	Proposal for HPV vaccine in Universal Immunization Programme (UIP), clinical trials conducted, and publication of outcomes.
17LSXII	20-07-2023	11-08-2023	Status of COVID-19 vaccination drive for children, reasons for non-availability of vaccine slots, and doses administered.
17LSXII	20-07-2023	11-08-2023	Nationwide immunization of Pneumococcal Conjugate Vaccine (PCV), details of vaccines administered, and financial support from WHO.
17LSXII	20-07-2023	11-08-2023	Steps to speed up vaccine development for emerging pathogens, and promotion of vaccines for diseases like polio and measles.
17LSXII	20-07-2023	11-08-2023	Fatalities due to rabies infection from dog bites, steps to improve availability of anti-rabies vaccines, and control of stray dogs.
17LSXIV	04-12-2023	22-12-2023	Children missing measles vaccine doses, steps for supplementary immunization, and catch-up vaccination age.
17LSXIV	04-12-2023	22-12-2023	Linkage between COVID-19 vaccine and heart attacks, review of potential correlation, and ongoing studies.
17LSXIV	04-12-2023	22-12-2023	Increase in dengue fever cases, measures to increase awareness, and development of a dengue vaccine.
17LSXV	31-01-2024	10-02-2024	Financial assistance provided to Odisha for various schemes, number of beneficiaries, and COVID-19 vaccinations.
17LSXV	31-01-2024	10-02-2024	Impact of COVID-19 vaccine on heart arteries, and steps to address the issue.
